# AlphaFold modeling uncovers global structural features of class I and class II fungal hydrophobins

**DOI:** 10.1002/pro.70279

**Published:** 2025-08-25

**Authors:** Li‐Yen Yang, Daniel J. Hicks, Paul S. Russo, Andrew C. McShan

**Affiliations:** ^1^ School of Chemistry and Biochemistry Georgia Institute of Technology Atlanta Georgia USA; ^2^ School of Materials Science and Engineering Georgia Institute of Technology Atlanta Georgia USA

**Keywords:** alphafold, computational biology, foldmason, foldseek, hydrophobins, protein structure, rosetta

## Abstract

Hydrophobins are a family of small fungal proteins that self‐assemble at hydrophobic–hydrophilic interfaces. Hydrophobins not only play crucial roles in filamentous fungal growth and development but also have attracted substantial attention due to their unique material properties. Structural characterization of class I and class II hydrophobins to date has been limited to a handful of proteins. While machine‐learning‐based structure prediction methods have the potential to exponentially expand our ability to define global structure–function relationships of biomolecules, they have not yet been extensively applied to hydrophobins. Here, we apply a suite of bioinformatics tools including Rosetta, AlphaFold, FoldMason, and Foldseek toward analysis, modeling, classification, and global comparison of class I and class II hydrophobins. We first probe the structural and energetic features of experimental class I and class II structures available in the Protein Data Bank. Using previously solved X‐ray and NMR structures, we benchmark the ability of AlphaFold to predict class I and class II hydrophobin folds. We explore the physicochemical properties of more than 7,000 class I and class II hydrophobins in the UniProt database. Then, using AlphaFold models, we classify the structural universe of all known class I and class II hydrophobins into six distinct clades. We also uncover putative non‐canonical features of hydrophobins, including extended N‐terminal tails, five disulfide bonds, polyhydrophobins, and non‐hydrophobin proteins containing hydrophobin‐like folds. Finally, we examine the ability of AlphaFold and Chai‐1 to model hydrophobin membrane binding, conformational changes, and self‐assembly of class I rodlets and class II meshes. Together, our results highlight that AlphaFold not only accurately models and enables the global comparison of features within the hydrophobin protein family but also uncovers new properties that can be further evaluated with experimentation.

## 1 INTRODUCTION

Hydrophobins are small proteins (typically <15 kDa) that play roles in filamentous fungal growth and development (Ball, Kwan, & Sunde, 2020; Wessels, 1997; Wessels, de Vries, Asgeirsdottir, & Schuren, 1991; Wessels, de Vries, Asgeirsdóttir, & Springer, 1991). Hydrophobins have been referred to as nature's Janus particles since they are amphipathic molecules with opposing hydrophobic and hydrophilic surfaces (Karadkar et al., 2023). The amphipathic properties of hydrophobins underpin their ability to transition from soluble to insoluble forms that self‐assemble at hydrophobic–hydrophilic interfaces (i.e., air‐water, air‐oil, air‐membrane, water‐solid, and fungal‐host) (Cheung, 2012; Szilvay et al., 2007; Wang et al., 2005). Hydrophobins are involved in a wide range of cellular processes in fungi, such as the formation of aerial hyphae, spores, and fruiting bodies (Ren et al., 2013; van Wetter et al., 2000; Wessels, 1997), the attachment of hyphae to hydrophobic surfaces (Wösten et al., 1994), and evasion of host immune responses (Aimanianda et al., 2009). For example, soluble hydrophobins secreted at the tip of the submerged hyphae diffuse into the aqueous environment and self‐assemble at the medium‐air interface, which results in a decrease in the water surface tension that allows hyphae to grow in the air (Cai et al., 2021; Wösten et al., 1999). Due to their unique material‐like properties, hydrophobins also have a variety of industrial and medical applications, such as surface functionalization, altering wettability, amphipathic solid films, deicing, emulsification, antifouling, foaming, shape‐shifting materials, and aerogel coating (Khalesi et al., 2015; Rojas‐Osnaya et al., 2024; Stanzione et al., 2022; Szilvay et al., 2007; Zhang et al., 2019). Example applications include hydrophobin‐coated nanoparticles for drug delivery (Valo et al., 2010; Zhao et al., 2016) and the modification of plastic materials for biodegradation (Khatua et al., 2024; Piscitelli et al., 2017; Stanzione et al., 2022).

Hydrophobins are typically classified into two distinct families, class I (PFAM PF01185) and class II (PFAM PF06766), based on solvent solubility, amino acid hydropathy patterns, and cysteine residue spacing within the amino acid sequence (Kershaw & Talbot, 1998; Rojas‐Osnaya et al., 2024; Wösten, 2001). Arguably, the most well‐studied hydrophobins from a functional perspective are the class I EAS from *Neurospora crassa* and the class II HFBI / HFBII molecules from *Trichoderma reesei* (Hakanpää, Szilvay, et al., 2006; Linder et al., 2001; Macindoe et al., 2012; Mackay et al., 2001; Magarkar et al., 2014; Nakari‐Setälä et al., 1996; Nakari‐Setälä et al., 1997). Phylogenetic and structural analyses suggest that the class I family can further be separated into proteins originating from Ascomycota (class IA) and Basidiomycota (class IB) fungal phyla (Gandier et al., 2017; Kenward et al., 2020). A third family termed class III (or intermediate/mixed) has also been proposed, though it is sometimes considered a special case within class I / class II (Jensen et al., 2010). Most hydrophobins contain eight cysteine residues that form four intramolecular disulfide bonds thought to stabilize the tertiary fold (Ren et al., 2013; Wösten, 2001). The cysteine residues/disulfide bonds may (for class II) or may not (for class I) play direct roles in self‐assembly (de Vocht et al., 2000; Kershaw et al., 2005; Sallada et al., 2018). Hydrophobins typically contain an N‐terminal secretion signal peptide (cleaved to furnish the mature hydrophobin), an intrinsically disordered N‐terminal tail, and the core folded hydrophobin domain (Kottmeier et al., 2011). Generally, the core folded domain contains a mix of random coils/loops, short α‐helices, and anti‐parallel β‐sheet secondary structural elements that form around a central β‐barrel stabilized by disulfide bonds (Ball, Pham, et al., 2020; Gandier et al., 2017; Hakanpää et al., 2004; Kwan et al., 2008). The hydrophobin loops are typically referred to with nomenclature defining the intercysteine residues (Rojas‐Osnaya et al., 2024). For example, C7‐C8 defines the loop formed by the residues spanning between Cys 7 and Cys 8 (Kwan et al., 2008; Macindoe et al., 2012; Tanaka et al., 2014).

There have been 12 class I and 9 class II experimental structures solved by either X‐ray crystallography or solution NMR spectroscopy (Supplementary Table S1 – EAS, DewA, MPG1, SC16, SLH4, WI1, PC1, and RodA for class I; HFBI, HFBII, and NC2 for class II). The class I hydrophobin EAS from *Neurospora crassa* contains a four‐stranded β‐barrel core with surrounding β‐strands and dynamic disordered regions where charged residues (i.e., Asp and Lys) and hydrophobic residues (i.e., Ile, Ala, Leu) are localized on opposing surfaces (Kwan et al., 2006; Macindoe et al., 2012; Mackay et al., 2001). Likewise, the class II hydrophobins HFBI and HFBII from *Trichoderma reesei* contain a four‐stranded β‐barrel core flanked by an α‐helix and conformationally labile loops with opposing hydrophilic and hydrophobic surfaces (Hakanpää et al., 2004; Hakanpää, Linder, et al., 2006; Hakanpää, Szilvay, et al., 2006). Some hydrophobins undergo post‐translational modifications, such as glycosylation (de Vocht et al., 1998; Linder et al., 2005). Glycosylation is not a strict requirement for self‐assembly since recombinant non‐glycosylated hydrophobins expressed in *E. coli* can readily self‐assemble and not all hydrophobins are glycosylated (de Vocht et al., 1998; Linder et al., 2005). Many secreted hydrophobins exist as soluble monomeric, dimeric, or tetrameric species when not in direct contact with hydrophobic–hydrophilic interfaces (Kisko et al., 2008; Mackay et al., 2001; Morris et al., 2013; Wang, Graveland‐Bikker, et al., 2004).

Upon contact with a hydrophobic–hydrophilic interface, structural changes have been reported to accompany hydrophobin self‐assembly (de Vocht et al., 1998; de Vocht et al., 2002; Kallio et al., 2007; Pham et al., 2018; Sunde et al., 2008; Wang et al., 2002; Wang, Permentier, et al., 2004). Class I hydrophobins form monolayers of amyloid‐like fibrils (i.e., rodlets) rich in β‐sheet characteristics that undergo amyloid‐specific interactions with Congo red and Thioflavin‐T (de Vries et al., 1993; Kwan et al., 2006; Mackay et al., 2001; Shanmugam et al., 2019). The interaction between the stacked β‐sheet of the amyloid‐like hydrophobin fibrils and the dyes generates unique spectroscopic properties: gold‐green birefringence from Congo red and a fluorescence emission maximum shift to 485 nm from Thioflavin‐T (Biancalana et al., 2009; Howie et al., 2008). Class I hydrophobins have been shown to transition from a right‐hand twisted β‐structure in monomeric forms to a relaxed β‐structure in the assembled amyloid‐like rodlets (de Vocht et al., 2002; Pham et al., 2018). The rodlets have been characterized as unbranched fibrils of 10 nm in width and 35–250 nm in length that spontaneously assemble at hydrophobic–hydrophilic interfaces and then associate laterally to form amphipathic monolayers (Dempsey & Beever, 1979; Macindoe et al., 2012; Terauchi et al., 2020). The disordered N‐terminal tail seems to be dispensable for self‐assembly (Vergunst & Langelaan, 2022). For the class I hydrophobin EAS, the C3‐C4 loop is not required for rodlet formation and surface activity (Kwan et al., 2008). Instead, mutagenesis and biophysical assays, supported by the generation of a chimeric hydrophobin, have identified that the hydrophobic C7‐C8 loop (residues F72‐N76 corresponding to sequence FLIIN for EAS) is responsible for rodlet formation, enabling data‐driven in silico modeling of the cross‐β assembled rodlet structure (Kwan et al., 2006; Kwan et al., 2008; Macindoe et al., 2012).

The architecture of amphipathic monolayers formed by the class II hydrophobins is in stark contrast to the rodlets formed by class I molecules (Lo et al., 2014). Class II assembled monolayers are not amyloid‐like rodlets, but instead form mesh‐like structures with repeating polygonal/hexagonal arrangements of 1 to 2 nm thickness and 20 to 90 nm diameter (Chang et al., 2020; Magarkar et al., 2014; Ren et al., 2014; Szilvay et al., 2007). Structures of HFBI and HFBII in the presence of detergents and polystyrene nanoparticles provide insights into binding to hydrophobic surfaces and multimerization: HFBI and HFBII form dimers via their hydrophobic surfaces, which then laterally interact at hydrophilic interfaces to assemble into monolayers (Hakanpää, Szilvay, et al., 2006; Kallio et al., 2007; Kallio & Rouvinen, 2011). Class II hydrophobins have been suggested to undergo an increase in α‐helical content during transitions from monomeric to the assembled form (Askolin et al., 2006). Deletions of residues in certain loops (i.e., the C4‐C5 or C7‐C8 loops) delay self‐assembly of class II hydrophobins (Gallo et al., 2023; Lienemann et al., 2013; Valsecchi et al., 2019). For both class I and class II hydrophobins, the self‐assembly process may be modulated by pH, temperature, ionic strength, pressure, detergent, acids, or organic solvents (Gravagnuolo et al., 2016; Pham et al., 2018; Wösten & de Vocht, 2000).

The diverse biological functions and materials applications of hydrophobins call for more studies on predicting structure–function relationships that underpin the many features of hydrophobin activity. Previous reports have applied computational tools spanning molecular dynamics simulations, structure modeling, and bioinformatic analysis of sequence/physicochemical properties toward advancing our understanding of hydrophobins (Bouqellah & Farag, 2023; Chang et al., 2020; de Simone et al., 2012; Gandier et al., 2017; Jensen et al., 2010; Mereghetti & Wade, 2011). Machine‐learning‐based tools for structure prediction, such as AlphaFold (Abramson et al., 2024; Agarwal & McShan, 2024; Jumper et al., 2021), have the potential to provide novel insights into (i) hydrophobins where experimental structures are unavailable, and (ii) the global features of all known hydrophobins. To date, the ability of these tools toward global characterization, classification, and discovery of new hydrophobin properties has not yet been thoroughly evaluated. Although the abilities of AlphaFold are already established for many proteins (Abramson et al., 2024; Agarwal & McShan, 2024; Akdel et al., 2022; Jumper et al., 2021), application to hydrophobins is especially important because they are difficult to crystallize.

Here, we use a suite of bioinformatics tools including Rosetta, AlphaFold, Chai‐1, FoldMason, and Foldseek for analysis, structural modeling, classification, and global comparison of class I and class II hydrophobins. We first probe the structural and energetic features of experimental class I and class II hydrophobin structures available in the Protein Data Bank. Using previously solved X‐ray and NMR structures, we benchmark the ability of AlphaFold to predict class I and class II hydrophobin folds across a range of targets. We explore the physicochemical properties of all class I and class II hydrophobins in the UniProt database. Then, using AlphaFold models, we evaluate the structural universe of class I and class II hydrophobins. AlphaFold models also allow us to uncover putative non‐canonical features of hydrophobins, which are compared to the canonical features. Finally, we examine the ability of AlphaFold and Chai‐1 to model self‐assembly of class I rodlets and class II meshes. Together, our results highlight that AlphaFold accurately models both class I and class II hydrophobins with some limitations, and offer working models that provide insights into new putative hydrophobin properties.

## 2 METHODS

### 2.1 Curation of class I and class II hydrophobin sequences and structures

The UniProt database was mined for class I and class II hydrophobins sequences with in‐house scripts combining UniProt's programmatic access and Biopython (UniProt Consortium, 2008). Class I and class II sequences were distinguished with *Family & Domains* search query terms “fungal hydrophobin family” (for class I) and “cerato‐ulmin hydrophobin family” (for class II). Ultimately, a total of 5414 class I and 1442 class II hydrophobin amino acid sequences were obtained in FASTA format. Using UniProt accession numbers, we also mined the RCSB Protein Data Bank (PDB) and AlphaFold Protein Structure Database for all class I and class II hydrophobin atomic structures and models, respectively (Berman et al., 2000; Varadi et al., 2022). From the PDB, we obtained a total of 12 fungal class I and 9 class II structures, either from X‐ray crystallography or solution NMR, in.pdb format (Supplementary Table S1).

### 2.2 Analysis of hydrophobin electrostatics, energetics, and physicochemical properties

PDB files were analyzed in PyMOL v3.0.4. Electrostatic surface potentials were calculated in PyMOL with the Adaptive Poisson–Boltzmann Solver (APBS) Plugin (Jurrus et al., 2018). Structures were prepared with the PDB2PQR method (Dolinsky et al., 2004), APBS maps were calculated with 0.5 grid spacing, and contour scaling in the Connolly surface method ranged from −5 kT/e (red, negative) to +5 kT/e (blue, positive).

Total energies of all 12 fungal class I and 9 class II structures were determined in the *Rosetta* computational modeling suite (Alford et al., 2017). For comparison with other small, globular proteins, analogous calculations were performed on separate SH3 domains (*n* = 13 with PDB IDs 1K76, 1NYF, 1ZLM, 2A08, 2HDA, 2VVK, 3C0C, 3I35, 3UA6, 4JZ4, 5NV1, 7A2J, 7JT9) and acyl carrier proteins (*n* = 8 with PDB IDs 1HY8, 1L0H, 1T8K, 2EHS, 2FAC, 2K92, 2L0Q, 7E42). The REF2015 forcefield of *Rosetta* v2021.16 was used to minimize bond angles/geometry and side‐chain rotamer conformations with Idealize and Relax applications, respectively (Nivón et al., 2013). Per‐residue contributions to the total energy were determined by the Residue Energy Breakdown application (Leman et al., 2020). The following code was used:


idealize_jd2.static.macosclangrelease ‐s *.pdb


relax.static.macosclangrelease ‐s *_0001.pdb ‐ex1 ‐ex2


python3 extract_scores.py


residue_energy_breakdown.static.macosclangrelease ‐s *_0001_0001.pdb ‐out:file:silent energy_breakdown.sc


awk '{print $4, $7, $28, $NF'} energy_breakdown.sc | sort ‐nk3 > sorted_energy_breakdown_totalscore.txt





Solvent‐accessible surface area (SASA) values were determined from the Rosetta idealized/relaxed PDB files using PyMOL with the command:


set dot_solvent, 1; set dot_density, 4; set solvent_radius, 1.4; get_area





The percentage of amino acid types in each sequence was determined using in‐house scripts utilizing Biopython's Bio.PDB PDBParser module.

For characterization of physicochemical properties: sequence length of full‐length class I and class I hydrophobins was obtained from UniProt and then evaluated with Biopython's Bio.Seq module. The total count of cysteine amino acids was determined over full‐length sequences with Biopython's Bio.SeqUtils.ProtParam Protein Analysis module (Wilkins et al., 1999). Isoelectric point was predicted with Biopython's Bio.SeqUtils.IsoelectricPoint module according to Bjellqvist's method (Bjellqvist et al., 1993). The GRAVY (Grand Average of Hydropathy) score was determined according to the Kyte and Doolittle method (Kyte & Doolittle, 1982) with Biopython's Bio.SeqUtils.ProtParam Protein Analysis module.

All scripts are freely available (see Data Availability section).

### 2.3 AlphaFold2 benchmark of class I and class II hydrophobin structures

AlphaFold2 models from the AlphaFold Protein Structure Database were compared with experimental structures from the PDBs of 21 experimental hydrophobin structures (Supplementary Table S1). SWISS‐MODEL's Structure Assessment tool (https://swissmodel.expasy.org/assess) was used to determine Cα root‐mean‐square deviation (Cα RMSD, Å), Cα local distance difference test (lDDT‐Cα), and template modeling score (TM‐score) (Kabsch, 1976; Mariani et al., 2013; Waterhouse et al., 2024; Zhang & Skolnick, 2004). For NMR ensembles, values are reported for the NMR model with the lowest Cα RMSD relative to the AlphaFold2 model.

### 2.4 Structure prediction of class I and class II hydrophobins

Several hydrophobins or proteins containing hydrophobin‐like domains did not have precalculated AlphaFold models available in the AlphaFold Protein Structure Database. In these cases, we generated models with AlphaFold3 using default parameters (Abramson et al., 2024). Random seeds were used, and the top model was chosen based on overall pTM score.

For benchmarking hydrophobin membrane binding and self‐assembly, AlphaFold3 modeling was performed on Georgia Tech's high‐performance cluster as briefly described below.

Example.json file for running AlphaFold3 with 104 different model systems with 8 class I hydrophobin sequences (EAS Δ15) and 50 oleic acid molecules (OLA):


{


 "name": "EAS_detergent_octamer",


 "modelSeeds": [


  99993, 18, 1981, 39, 950, 12, 946, 955, 972, 41, 24, 985, 970, 21, 944, 8, 989, 956, 937, 962,


  19 960 25976 444974 11 378947 53 929 936 49 923 927 910 7 929 16 991 986 4 33,


  959, 10, 22, 17, 31, 969, 962, 25, 9782517, 3, 853, 845, 832, 819, 775, 780, 753, 735, 718, 640,


  598 590 579 550 505 473 462 430 111402 365 350 325 310 284 276 264 252 231,


  223, 207, 195, 182, 19024551, 140, 132, 116, 1032, 91, 77, 66, 60, 44, 36, 28, 15, 6, 2, 1, 5, 13,


  9, 17, 27, 38


 ],


 "sequences": [


  { "protein": { "id": "A", "sequence": "SATTIGPNTCSIDDYKPYCCQSMSGSASLGCVVGVIGSQCGASVKCCKDDVTNTGNSFLIINAANCVA" } },


  { "protein": { "id": "B", "sequence": "SATTIGPNTCSIDDYKPYCCQSMSGSASLGCVVGVIGSQCGASVKCCKDDVTNTGNSFLIINAANCVA" } },


  { "protein": { "id": "C", "sequence": "SATTIGPNTCSIDDYKPYCCQSMSGSASLGCVVGVIGSQCGASVKCCKDDVTNTGNSFLIINAANCVA" } },


  { "protein": { "id": "D", "sequence": "SATTIGPNTCSIDDYKPYCCQSMSGSASLGCVVGVIGSQCGASVKCCKDDVTNTGNSFLIINAANCVA" } },


  { "protein": { "id": "E", "sequence": "SATTIGPNTCSIDDYKPYCCQSMSGSASLGCVVGVIGSQCGASVKCCKDDVTNTGNSFLIINAANCVA" } },


  { "protein": { "id": "F", "sequence": "SATTIGPNTCSIDDYKPYCCQSMSGSASLGCVVGVIGSQCGASVKCCKDDVTNTGNSFLIINAANCVA" } },


  { "protein": { "id": "G", "sequence": "SATTIGPNTCSIDDYKPYCCQSMSGSASLGCVVGVIGSQCGASVKCCKDDVTNTGNSFLIINAANCVA" } },


  { "protein": { "id": "H", "sequence": "SATTIGPNTCSIDDYKPYCCQSMSGSASLGCVVGVIGSQCGASVKCCKDDVTNTGNSFLIINAANCVA" } },


  { "ligand": { "id": "OA", "ccdCodes": ["OLA"] } },


  { "ligand": { "id": "OB", "ccdCodes": ["OLA"] } },


  { "ligand": { "id": "OC", "ccdCodes": ["OLA"] } },


  { "ligand": { "id": "OD", "ccdCodes": ["OLA"] } },


  { "ligand": { "id": "OE", "ccdCodes": ["OLA"] } },


  { "ligand": { "id": "OF", "ccdCodes": ["OLA"] } },


  { "ligand": { "id": "OG", "ccdCodes": ["OLA"] } },


  { "ligand": { "id": "OH", "ccdCodes": ["OLA"] } },


  { "ligand": { "id": "OI", "ccdCodes": ["OLA"] } },


  { "ligand": { "id": "OJ", "ccdCodes": ["OLA"] } },


  { "ligand": { "id": "OK", "ccdCodes": ["OLA"] } },


  { "ligand": { "id": "OL", "ccdCodes": ["OLA"] } },


  { "ligand": { "id": "OM", "ccdCodes": ["OLA"] } },


  { "ligand": { "id": "ON", "ccdCodes": ["OLA"] } },


  { "ligand": { "id": "OO", "ccdCodes": ["OLA"] } },


  { "ligand": { "id": "OP", "ccdCodes": ["OLA"] } },


  { "ligand": { "id": "OQ", "ccdCodes": ["OLA"] } },


  { "ligand": { "id": "OR", "ccdCodes": ["OLA"] } },


  { "ligand": { "id": "OS", "ccdCodes": ["OLA"] } },


  { "ligand": { "id": "OT", "ccdCodes": ["OLA"] } },


  { "ligand": { "id": "OU", "ccdCodes": ["OLA"] } },


  { "ligand": { "id": "OV", "ccdCodes": ["OLA"] } },


  { "ligand": { "id": "OW", "ccdCodes": ["OLA"] } },


  { "ligand": { "id": "OX", "ccdCodes": ["OLA"] } },


  { "ligand": { "id": "OY", "ccdCodes": ["OLA"] } },


  { "ligand": { "id": "OZ", "ccdCodes": ["OLA"] } },


  { "ligand": { "id": "ZA", "ccdCodes": ["OLA"] } },


  { "ligand": { "id": "ZB", "ccdCodes": ["OLA"] } },


  { "ligand": { "id": "ZC", "ccdCodes": ["OLA"] } },


  { "ligand": { "id": "ZD", "ccdCodes": ["OLA"] } },


  { "ligand": { "id": "ZE", "ccdCodes": ["OLA"] } },


  { "ligand": { "id": "ZF", "ccdCodes": ["OLA"] } },


  { "ligand": { "id": "ZG", "ccdCodes": ["OLA"] } },


  { "ligand": { "id": "ZH", "ccdCodes": ["OLA"] } },


  { "ligand": { "id": "ZI", "ccdCodes": ["OLA"] } },


  { "ligand": { "id": "ZJ", "ccdCodes": ["OLA"] } },


  { "ligand": { "id": "PA", "ccdCodes": ["OLA"] } },


  { "ligand": { "id": "PB", "ccdCodes": ["OLA"] } },


  { "ligand": { "id": "PC", "ccdCodes": ["OLA"] } },


  { "ligand": { "id": "PD", "ccdCodes": ["OLA"] } },


  { "ligand": { "id": "PE", "ccdCodes": ["OLA"] } },


  { "ligand": { "id": "PF", "ccdCodes": ["OLA"] } },


  { "ligand": { "id": "PG", "ccdCodes": ["OLA"] } },


  { "ligand": { "id": "PH", "ccdCodes": ["OLA"] } },


  { "ligand": { "id": "PI", "ccdCodes": ["OLA"] } },


  { "ligand": { "id": "PJ", "ccdCodes": ["OLA"] } },


  { "ligand": { "id": "PK", "ccdCodes": ["OLA"] } },


  { "ligand": { "id": "PL", "ccdCodes": ["OLA"] } },


  { "ligand": { "id": "PM", "ccdCodes": ["OLA"] } },


  { "ligand": { "id": "PN", "ccdCodes": ["OLA"] } },


  { "ligand": { "id": "PO", "ccdCodes": ["OLA"] } },


  { "ligand": { "id": "PP", "ccdCodes": ["OLA"] } },


  { "ligand": { "id": "PQ", "ccdCodes": ["OLA"] } },


  { "ligand": { "id": "PR", "ccdCodes": ["OLA"] } },


  { "ligand": { "id": "PS", "ccdCodes": ["OLA"] } },


  { "ligand": { "id": "PT", "ccdCodes": ["OLA"] } },


  { "ligand": { "id": "PU", "ccdCodes": ["OLA"] } },


  { "ligand": { "id": "PV", "ccdCodes": ["OLA"] } },


  { "ligand": { "id": "PW", "ccdCodes": ["OLA"] } },


  { "ligand": { "id": "PX", "ccdCodes": ["OLA"] } },


  { "ligand": { "id": "PY", "ccdCodes": ["OLA"] } },


  { "ligand": { "id": "PZ", "ccdCodes": ["OLA"] } }


 ],


 "dialect": "alphafold3",


 "version": 1


}





Example of the SLURM format submission script:


#!/bin/bash


#SBATCH ‐‐job‐name=af3 # name of the job


#SBATCH ‐‐account=accountname # charge account


#SBATCH ‐‐time=72:00:00 # Time limit hrs:min:sec


#SBATCH ‐o slurm_run_%j_af3_output.txt


#SBATCH ‐e slurm_run_%j_af3_log.txt


#SBATCH ‐‐mem=80GB


#SBATCH ‐q inferno # QOS name


#SBATCH ‐‐gres=gpu:A100:1


# go into the submission directory


cd $SLURM_SUBMIT_DIR


# run AF3


input='./af_input'


output='./af_output'


echo $input


echo $output


# run the af3 structure prediction script


apptainer exec ‐‐bind "$input":/root/af_input ‐‐bind "$output":/root/af_output ‐‐bind /storage/cedar/cedar0/cedarp‐amcshan3‐0/alphafold3_param:/root/models ‐‐bind /storage/cedar/cedar0/cedarp‐amcshan3‐0/alphafold3_database:/root/public_databases ‐‐nv /storage/cedar/cedar0/cedarp‐amcshan3‐0/alphafold3/image.sif python /storage/cedar/cedar0/cedarp‐amcshan3‐0/alphafold3/run_alphafold.py ‐‐json_path=/root/af_input/EAS.json ‐‐model_dir=/root/models ‐‐db_dir=/root/public_databases ‐‐output_dir=/root/af_output





Chai‐1, an AlphaFold3 mimic (Discovery et al., 2024), was also used to model self‐assembly of EAS Δ15 using the Chai‐1 webserver (https://lab.chaidiscovery.com/dashboard). Five copies of EAS Δ15 were input to Chai‐1 together with distance restraints based on the expected amyloid‐like structure of EAS Δ15 (Macindoe et al., 2012):

**Table:** 

Type	Chain 1	Residue index 1	Chain 2	Residue index 2	Distance (Å)
Contact	A	N53	B	N53	4
Contact	B	N53	C	N53	4
Contact	C	N53	D	N53	4
Contact	D	N53	E	N53	4
Contact	A	N53	E	N53	20
Contact	A	L59	B	L59	4
Contact	B	L59	C	L59	4
Contact	C	L59	D	L59	4
Contact	D	L59	E	L59	4
Contact	A	L59	E	L59	20
Contact	A	L59	B	N53	11.5
Contact	B	L59	C	N53	11.5
Contact	C	L59	D	N53	11.5
Contact	D	L59	E	N53	11.5
Contact	A	N53	B	L59	11.5
Contact	B	N53	C	L59	11.5
Contact	C	N53	D	L59	11.5
Contact	D	N53	E	L59	11.5
Contact	A	F58	B	N53	12
Contact	A	I60	B	N53	12
Contact	A	T52	B	L59	12
Contact	A	T54	B	L59	12

### 2.5 Generation of sequence and structure‐based dendrograms

To generate a sequence‐based dendrogram, a multiple sequence alignment was built from all 6,920 hydrophobin sequences using Clustal Omega v1.2.4 (Sievers et al., 2011). Inclusion of full‐length sequences resulted in a noisy alignment due to the disordered N‐terminal region and signal sequence. Thus, amino acids preceding the first cysteine were omitted as described previously (Linder et al., 2005). Next, TrimAI was used to optimize the alignment by removing sequences with excessive gaps, resulting in a total of 6,754 sequences (Capella‐Gutiérrez et al., 2009). Finally, IQ‐TREE 2 was used to construct the sequence tree (Minh et al., 2020). The commands for the pipeline are as follows:


python3 make_fasta_trimcys.py


./clustal_omega ‐i hydrophobin_extra_trimmedCys.fasta ‐o hydrophobin_trimCys.aln ‐v ‐‐outfmt=clu –force


cd trimAl_Linux_x86‐64


./trimal ‐in hydrophobin_trimCys.aln ‐out hydrophobin_trimCys.trim ‐automated1


./iqtree3_intel ‐s hydrophobin_trimCys.trim





The resulting Newick format output was analyzed to generate an unrooted sequence‐based dendrogram in Interactive Tree of Life (iTOL) v7 (https://itol.embl.de/) (Letunic & Bork, 2024).

To generate a structure‐based dendrogram, a total of 6,920 AlphaFold models of fungal class I and class II hydrophobins were passed through the multiple structure alignment tool FoldMason (https://github.com/steineggerlab/foldmason) (Gilchrist et al., 2024). There was no filtering/preprocessing before or after running FoldMason. FoldMason was run in the basic MSA workflow mode as follows:


foldmason easy‐msa *.pdb result.fasta tmpFolder ‐‐report‐mode 1





The resulting Newick format output was analyzed to generate an unrooted structure‐based dendrogram in iTOL v7 (https://itol.embl.de/) (Letunic & Bork, 2024).

### 2.6 Identification of non‐canonical hydrophobins

To identify examples of putative hydrophobins with five disulfide bonds, we used in‐house scripts to filter all hydrophobin sequences containing 10 total cysteines with a sequence length of less than 250 amino acids. We then visualized the corresponding AlphaFold models to check whether all disulfide bonds were predicted to be oxidized as well as their placement relative to canonical hydrophobins. To identify examples of putative hydrophobins with extended N‐terminal tails, we used in‐house scripts to filter all hydrophobin sequences containing at least 70 amino acids before the first Cys residue. We then visualized the corresponding AlphaFold models to check whether a hydrophobin fold was present. To identify examples of polyhydrophobins, we used in‐house scripts to filter all hydrophobin sequences containing “trihydrophobin” or “pentahydrophobins” in the UniProt annotation. We also manually checked AlphaFold models of sequences >250 amino acids in length. We then visualized the corresponding AlphaFold models to check whether multiple hydrophobin folds were present. For comparison of non‐canonical hydrophobins with canonical hydrophobins, sequence alignments were performed with Clustal Omega v1.2.4 and processed with ESPript 3 (Robert & Gouet, 2014; Sievers et al., 2011).

### 2.7 Identification of hydrophobin‐like domains within other proteins

To identify non‐hydrophobin proteins containing hydrophobin‐like domains, class I hydrophobins (DewA‐PDB 2LSH, EAS‐PDB 2FMC, MPG1‐PDB 2 2N4O, PC1‐PDB 6E08, RodA‐PDB 6GCJ, SC16‐PDB 2NBH, SLH4‐PDB 5W0Y, WI1‐PDB 6E9M) and class II hydrophobins (HFBI‐PDB 2FZ6, HFBII‐1R2M, NC2‐4AOG) were used as input to search eight structural databases (AlphaFold/Proteome v4, AlphaFold/Swiss‐Prot v4, AlphaFold/UniProt50 v4, CATH50 v4.3.0, GMGCL 2024, MGnify‐ESM30 v1, PDB100 20,240,101, BFMD 20240623) with Foldseek in 3Di/AA mode with the iterative search option without any taxonomic filters (https://search.foldseek.com/search) (van Kempen et al., 2024). All hits that were canonical hydrophobins were filtered out with in‐house scripts and manual inspection. Cα RMSD values between reference structures and the hydrophobin‐like domains were determined in PyMOL v3.0.4. Sequence alignments were performed with Clustal Omega 1.2.4 and processed with ESPript 3 (Robert & Gouet, 2014; Sievers et al., 2011). MSA coverage heatmaps were obtained using ColabFold v1.5.5: AlphaFold2 using MMseqs2 (Mirdita et al., 2022) at the following link:

https://colab.research.google.com/github/sokrypton/ColabFold/blob/main/AlphaFold2.ipynb.

### 2.8 Prediction of aggregation potential and phase separation potential

The aggregation potential of putative hydrophobins was performed with the AggreProt Predictor webserver v1 (https://loschmidt.chemi.muni.cz/aggreprot/) (Planas‐Iglesias et al., 2024). Canonical or non‐canonical hydrophobin sequences were input into the server and jobs were submitted using default parameters. The potential for hydrophobins to undergo phase separation was determined using the ParSe tool v2 (Ibrahim et al., 2023). Canonical or non‐canonical hydrophobin sequences were input into two different server sites (https://stevewhitten.github.io/Parse_v2_FASTA/ and https://stevewhitten.github.io/Parse_v2_web/) using default parameters.

## 3 RESULTS

### 3.1 Class I hydrophobins exhibit greater structural diversity than class II hydrophobins

**FIGURE 1:**
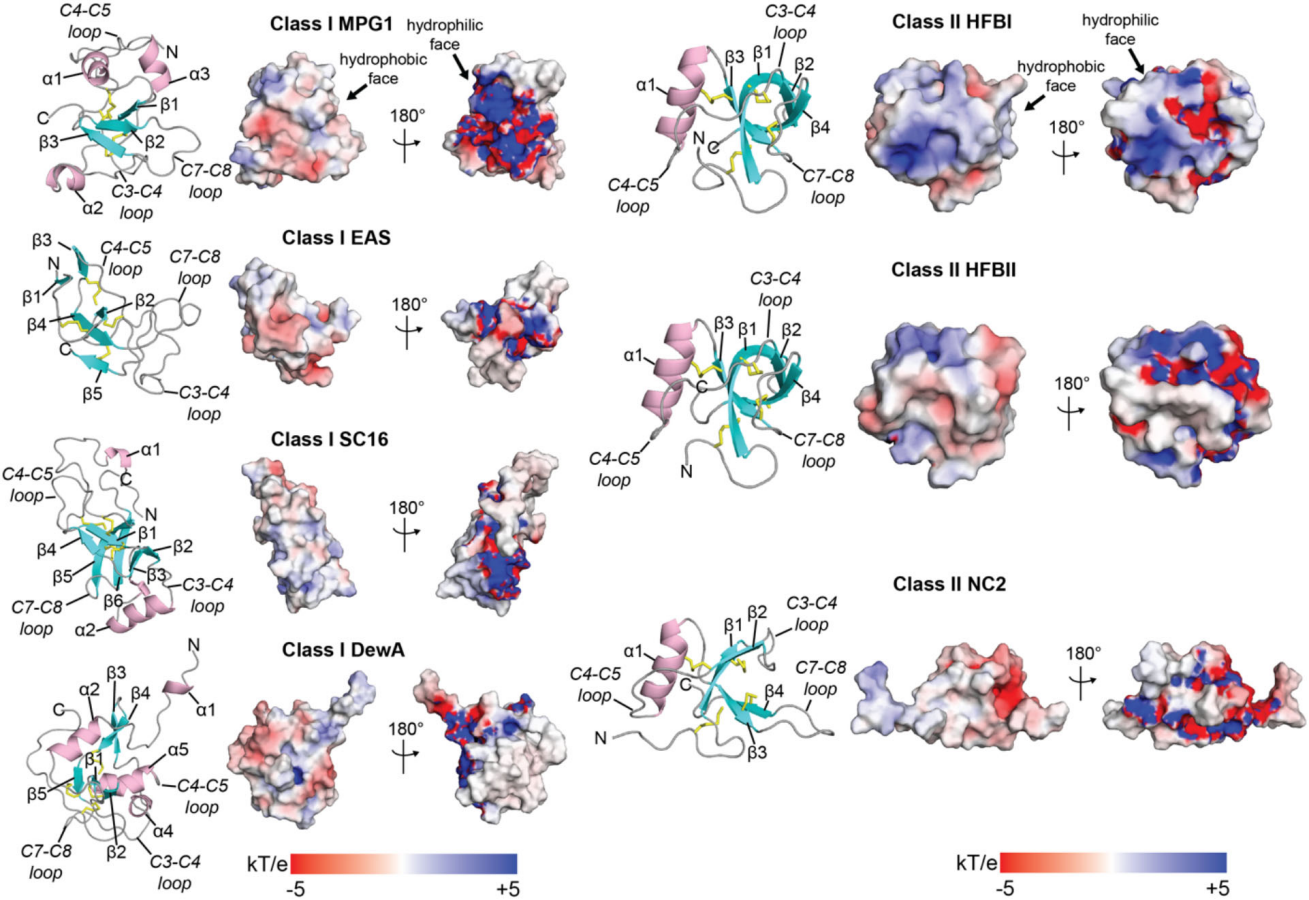
General structural features of class I and class II hydrophobins. Examples of class I (left) and class II (right) hydrophobin structures from the Protein Data Bank. Cartoon representations are shown with β‐strands in cyan, α‐helices in pink, and coils in gray. Disulfide bonds are shown as yellow sticks. Each cartoon is also accompanied by an electrostatic surface visualization calculated with the Adaptive Poisson–Boltzmann Solver (APBS plugin of PyMOL) (Unni et al., 2011). The contour scale for the APBS visualization is −5 kT/e (red, negative) to +5 kT/e (blue, positive). The hydrophobic (white) and hydrophilic (dark blue/dark red) faces of the amphipathic hydrophobin surface are noted. The following structures are shown: MPG1 from *Magnaporthe oryzae* (PDB ID 2N4O‐Class I) (Pham et al., 2016), EAS from *Neurospora crassa* (PDB ID 2FMC‐Class I) (Kwan et al., 2006), SC16 from *Schizophyllum commune* (PDB ID 2NBH‐Class I) (Gandier et al., 2017), DewA from *Aspergillus nidulans* (PDB ID 2LSH‐Class I) (Morris et al., 2013), HFBI from *Trichoderma reesei* (PDB ID 2FZ6‐Class II) (Hakanpää, Szilvay, et al., 2006), HFBII from *Trichoderma reesei* (PDB ID 1R2M‐Class II) (Hakanpää, Szilvay, et al., 2006), and NC2 from *Neurospora crassa* (PDB ID 4AOG‐Class II) (Ren et al., 2014). The N‐terminus (“N”), C‐terminus (“C”), and secondary structure elements (α‐helix, β‐strands, and intercysteine loops) are noted.

As a baseline for defining structure–function relationships, we first compared and contrasted the features of class I and class II fungal hydrophobins with previously determined experimental structures available in the PDB (Supplementary Table S1) (Berman et al., 2000). We visualized the general structural features of eight class I hydrophobins (MPG1, EAS, SC16, DewA, SLH4, PC1, WI1, and RodA) and three class II hydrophobins (HFBI, HFBII, and NC2) (Figure 1, Supplementary Figures S1 and S2) Vergunst et al., 2022. Both class I and class II hydrophobins showed a centralized β‐barrel core composed of anti‐parallel β‐sheets stabilized by four disulfide bonds. Both class I and class II hydrophobins exhibited amphipathic surfaces with a charged hydrophilic face and a mostly neutral hydrophobic face. Electrostatic surface potentials as determined with the APBS method revealed that the precise surface electrostatic potential profile differs from protein to protein (Unni et al., 2011). For example, the hydrophilic face of DewA showed a more localized electrostatic surface potential, whereas it was more evenly distributed for MPG1 (Figure 1). The hydrophobic faces of both PC1 and WI1 were more negatively charged relative to other class I hydrophobins (Supplementary Figure S1). As previously reported (Gandier et al., 2017), class I hydrophobins showed greater structural diversity than class II hydrophobins, as highlighted by differences in the number/arrangement of α‐helices flanking the core domain, the number/arrangement of β‐strands in the core domain, and the length/orientation of the intercysteine loops (C1‐C2, C3‐C4, C4‐C5, C7‐C8). Class II hydrophobins HFBI, HFBII, and NC2 had highly similar folds and electrostatic surface potentials around the central β‐barrel composed of four anti‐parallel β‐strands flanked by a single α‐helix (Figure 1). The hydrophobic face of HFBI and HFBII, which is lined by the C1‐C2, C3‐C4, and C7‐C8 loops, has been shown to provide a surface for hydrophobin oligomerization as well as association with different types of detergents (Supplementary Figure S2). Together, this analysis highlights previous reports that class I hydrophobins exhibit greater structural diversity than class II hydrophobins, and unique features underpin their differences in self‐assembly (Linder et al., 2005; Wösten, 2001).

### 3.2 Van der Waals attractive forces, coulombic electrostatics, and backbone hydrogen bonds stabilize class I and class II hydrophobin structures

**FIGURE 2:**
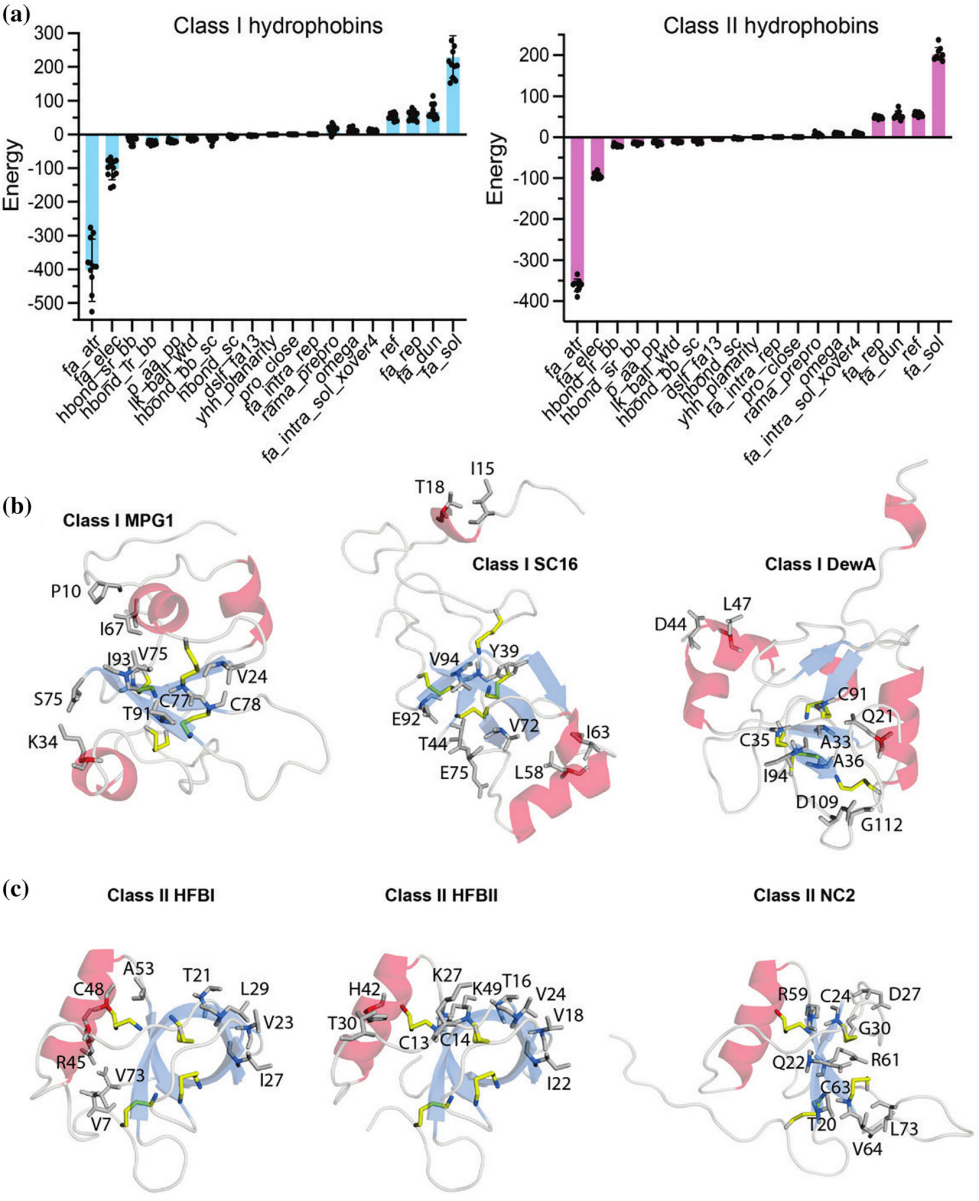
*Rosetta* evaluation of the molecular forces contributing to hydrophobin stability. (a) Histogram of contributions of each *Rosetta* REF2015 score function energy term to the total energy of class I and class II hydrophobins from the Protein Data Bank. Data are mean ± standard deviation where error bars are derived from separate calculations of different hydrophobin structures; *n* = 12 for class I and *n* = 9 for class II (see Supplementary Table S1). (b) and (c) show summaries of the top five molecular interactions (residue‐residue pairs) for each structure: MPG1 from *Magnaporthe oryzae* (PDB ID 2N4O‐Class I) (Pham et al., 2016), SC16 from *Schizophyllum commune* (PDB ID 2NBH‐Class I) (Gandier et al., 2017), DewA from *Aspergillus nidulans* (PDB ID 2LSH‐Class I) (Morris et al., 2013), HFBI from *Trichoderma reesei* (PDB ID 2FZ6‐Class II) (Hakanpää, Szilvay, et al., 2006), HFBII from *Trichoderma reesei* (PDB ID 1R2M‐Class II) (Hakanpää et al., 2004), and NC2 from *Neurospora crassa* (PDB ID 4AOG‐Class II) (Ren et al., 2014). Disulfide bonds are shown as yellow sticks.

To evaluate the types of molecular forces that stabilize class I and class II hydrophobin folds, we idealized bond angles/geometry, performed side‐chain rotamer energy minimization, and then scored each structure within the REF2015 forcefield of the *Rosetta* modeling suite (Alford et al., 2017; Leman et al., 2020). This allowed us to determine which of *Rosetta*'s 19 physics‐ and knowledge‐based energy terms contribute favorably or unfavorably to the total energy of the different classes of hydrophobins. Results were highly self‐consistent within the structures evaluated (Figure 2a). The total *Rosetta* energy values determined from the idealized and relaxed structures were −151.5 ± 47.7 Rosetta energy units (REU) over the 12 class I structures and −142.5 ± 16.8 REU across the 9 class II structures. The REF2015 energy terms that contributed most favorably to hydrophobin structures were Lennard‐Jones attractive forces (fa_atr), Coulombic electrostatic interactions (fa_elec), and short−/long‐range hydrogen bonding within the backbone (hbond_sr_bb and hbond_lr_bb). The REF2015 energy terms that contributed most unfavorably to hydrophobin structures were the Lazaridis–Karplus solvation energy (fa_sol), internal energy of side‐chain rotamers as derived from Dunbrack's statistics (fa_dun), Lennard‐Jones repulsive forces (fa_rep), and reference energies for each amino acid (ref). We also probed whether the residue‐residue interactions that most contributed to total energies were localized to specific regions of the hydrophobin structure. Broadly speaking, visualization of the top five pairs of interactions that contributed most favorably to the total energy reveals highly localized hydrophobic and electrostatic interactions between residues within the disulfide bond stabilized β‐sheet core (Figure 2b,c). Together, these results highlight self‐consistent patterns of Van der Waals attractive forces (i.e., dipole–dipole, dipole‐induced dipole, London dispersion), electrostatic interactions, and backbone hydrogen bonds that contribute to the overall favorable energetics of class I and class II hydrophobin monomeric structures.

We next asked whether the forces that stabilize hydrophobins were similar to those that stabilize other small globular proteins. The primarily β‐sheet SH3 domains and the primarily α‐helical acyl carrier proteins were used as model systems (Farmer et al., 2019; Kurochkina & Guha, 2012). The REF2015 energy terms that contributed most favorably (i.e., fa_atr, fa_elec, hbond_sr_bb, hbond_lr_bb) and least favorably (i.e., fa_sol) to hydrophobin structures were highly consistent with forces contributing to SH3 domain and acyl carrier protein stability (Supplementary Figure S3). Like hydrophobins, electrostatic surface potentials of SH3 domains showed opposing hydrophilic and hydrophobic surfaces, consistent with reports that SH3 ligand binding sites have a hydrophobic surface flanked by charged loops (Supplementary Figure S3) (Booker et al., 1993). In contrast, acyl carrier proteins exhibited a highly charged (mostly negative) surface lacking a major hydrophobic face, consistent with reports that negatively charged acyl carrier proteins interact with positively charged surfaces of cognate enzymes (Supplementary Figure S3) (Barajas et al., 2016). The SASAs and percentage of polar/charged/hydrophobic residues across hydrophobins, SH3 domain, and acyl carrier protein structures were also relatively consistent except for class I hydrophobins that showed a moderate increase in the SASA that could be attributed to the flexible disordered loops. According to this analysis, the forces stabilizing hydrophobins are similar to those underlying stability in other small, globular proteins. The unique features attributed to hydrophobin function are likely linked to a combination of their amphipathic nature, conformational plasticity, and sequence/structure features present in the hydrophobic intercysteine loops.

### 3.3 Physicochemical properties of class I and class II hydrophobins are well delineated

To study the global features of hydrophobins, we evaluated the physicochemical properties of all class I and class II hydrophobins extracted from the UniProt database. As others have done at a smaller scale (Bouqellah & Farag, 2023; Rineau et al., 2017; Zhao et al., 2021), we computationally probed sequence length, cysteine count, isoelectric point, and GRAVY score of each sequence. Most class I and class II hydrophobins had sequence lengths between 100 and 140, with a larger distribution of lengths observed for class I hydrophobins (Supplementary Figure S4a). Cysteine counts of class I and class II hydrophobins were heavily skewed to 8, consistent with four disulfide bonds within the core folded domain (Supplementary Figure S4b). Predicted isoelectric points (pI) were found to be clustered between 4 and 6 for both class I and class II hydrophobins (Supplementary Figure S4c). The range of predicted pI values is consistent with experimental zeta potential measurements (Terauchi et al., 2022; Yang et al., 2019). Given that the intracellular pH of filamentous fungi ranges from 7.4 to 7.7 (Bagar et al., 2009), most cytoplasmic hydrophobins would be negatively charged. Of note, the surface pH along growing hyphae ranges from 5 to 6.3, where hydrophobins could exhibit a less negative charge (Xiong et al., 2022). GRAVY scores, a measure of hydropathy where a higher positive value indicates greater hydrophobicity (Kyte & Doolittle, 1982), were primarily populated in the range from 0 to 1 for both class I and class II hydrophobins (Supplementary Figure S4d). These GRAVY scores highlight the unique hydrophobic properties of hydrophobins relative to the mean GRAVY scores across the proteomes of bacteria, archaea, and eukaryotes that range from −0.5 to −0.3 (Jin et al., 2021). Together, this global analysis shows that the physicochemical properties of class I and class II hydrophobins are well delineated and similar between the different classes.

### 3.4 AlphaFold2 robustly predicts structures of both class I and class II hydrophobins

**FIGURE 3:**
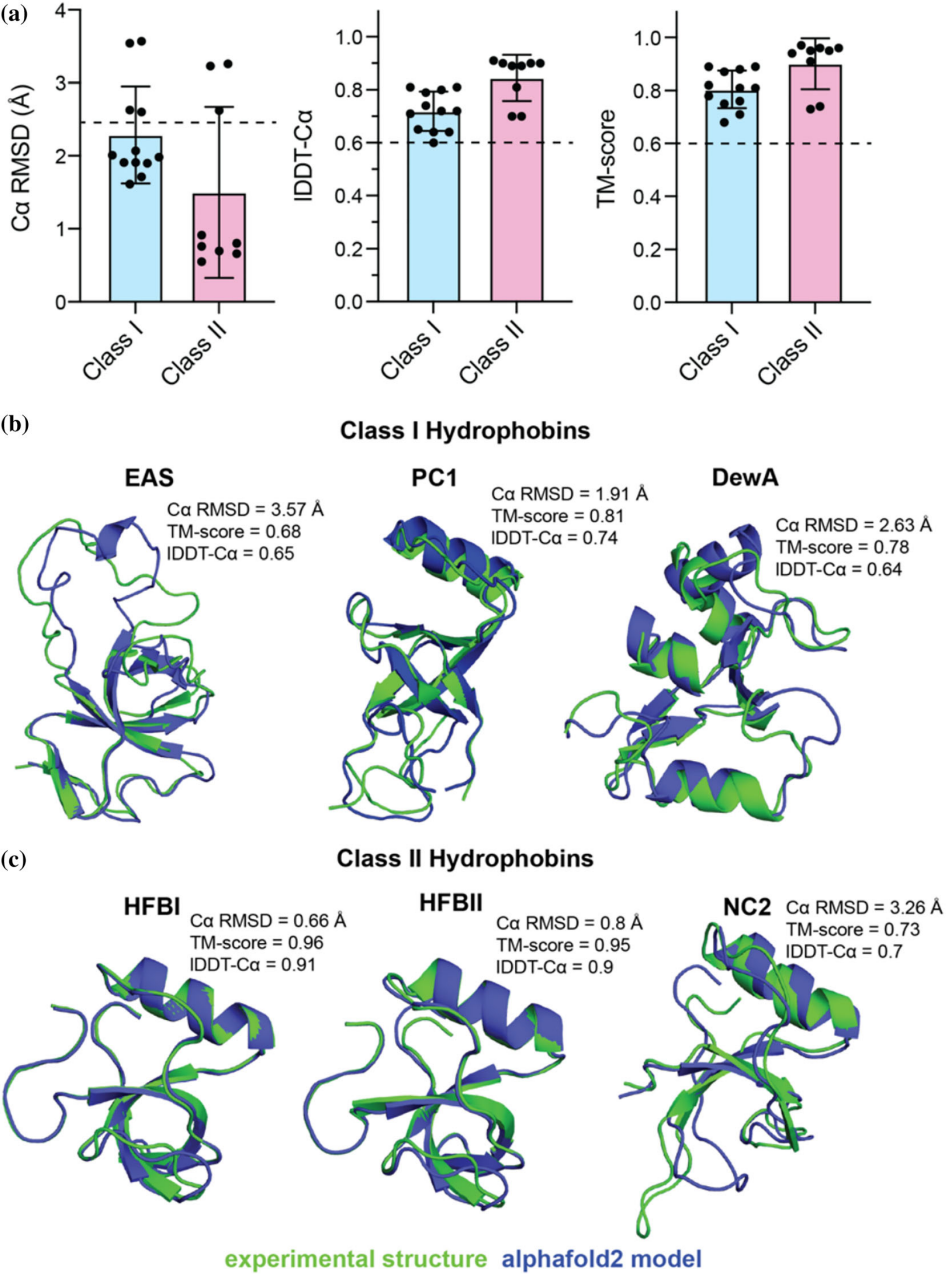
AlphaFold2 accurately predicts structures of class I and class II hydrophobins. (a) Results from benchmarking of AlphaFold2 on 21 class I and class II hydrophobin experimental structures. Root‐mean‐square deviation (Cα RMSD, Å), Local Distance Difference Test (lDDT‐Cα), and Template modeling score (TM‐score) for AlphaFold2 models relative to experimental structures are summarized. Error bars are derived from the mean ± the standard deviation across comparison of all structures within each class (see Supplementary Table S1). The dotted black lines in each graph denote standard cut‐offs that mark a good prediction (Cα RMSD <2.5 Å, lDDT‐Cα >0.6, TM‐score >0.6). (b) Overlay of AlphaFold2 models (blue) versus experimental structure (green) for class I hydrophobins. PDB IDs: 2FMC (EAS from *Neurospora crassa*) (Kwan et al., 2006), 6E98 (PC1 from *Phanerochaete carnosa*) (Kenward et al., 2020), 2LSH (DewA from *Aspergillus nidulans*) (Morris et al., 2013). (c) Overlay of AlphaFold2 models (blue) versus experimental structures (green) for class II hydrophobins. PDB IDs: 2GVM (HFBI from *Trichoderma reesei*) (Hakanpää, Szilvay, et al., 2006), 1R2M (HFBII from *Trichoderma reesei*) (Hakanpää et al., 2004), 4AOG (NC2 from *Neurospora crassa*) (Ren et al., 2014). For each AlphaFold3 model, the Cα RMSD, lDDT‐Cα, and TM‐score values relative to the experiment structure are noted. For clarity, experimental structures derived from solution NMR (PDB IDs 2FMC, 6E98, 2LSH, 4AOG) are shown with only the model with the lowest Cα RMSD value relative to the AlphaFold2 model.

We next benchmarked the ability of AlphaFold2 to predict structures of monomeric class I and class II hydrophobins. Toward quantitative evaluation, we used several structural comparison metrics, such as Cα RMSD, lDDT‐Cα, and TM‐score, to compare AlphaFold2 models with 21 experimental X‐ray or NMR structures (Supplementary Table S1) (Kabsch, 1976; Mariani et al., 2013; Waterhouse et al., 2024; Zhang & Skolnick, 2004). Cα RMSD measures the mean distance between corresponding atoms and represents a global superposition where values <1.5 Å represent the highest quality predictions, values from 1.5 to 2.5 Å represent high‐quality predictions, values between 2.5 to 4 Å represent intermediate‐quality predictions, and values above 4 Å represent failed predictions (Bornot et al., 2009; McPartlon & Xu, 2023). Because RMSD can be insensitive to local errors and may not reliably distinguish partially correct models from incorrect ones, multiple metrics of comparison are often used to quantify the accuracy of predicted models relative to native conformations (Jumper et al., 2021; Kufareva & Abagyan, 2012; Olechnovič et al., 2019). To better assess prediction accuracy, complementary metrics, such as TM‐score and lDDT‐Cα, are often used. TM‐score measures the mean distance between Cα atoms scaled by a length‐dependent distance parameter and represents a global superposition where values above 0.6 represent likely accurate predictions (Xu & Zhang, 2010; Zhang & Skolnick, 2004). lDDT‐Cα measures the mean fraction of preserved all‐atom distance for Cα atoms and presents local comparisons within defined tolerance thresholds where values above 0.6 represent likely accurate predictions (Jumper et al., 2021; Mariani et al., 2013). Within our benchmarking dataset, we observed Cα RMSD values of 2.2 ± 0.6 for class I and 1.4 ± 1.1 for class II, lDDT‐Cα values of 0.71 ± 0.07 for class I and 0.84 ± 0.08 for class II, and TM‐score values of 0.80 ± 0.07 for class I and 0.90 ± 0.09 for class II (Figure 3a). While several hydrophobin AlphaFold models fall outside the Cα RMSD quality threshold, almost all models were within the lDDT‐Cα and TM‐score cut‐off scores for moderate to high‐quality predictions, supporting the proper prediction of the global hydrophobin fold (Figure 3a).

Visual inspections of AlphaFold2 models versus experimental structures for class I hydrophobins and class II hydrophobins highlight the robustness of the structure prediction within the β‐barrel core (Figure 3b,c, Supplementary Figures S5 and S6). In terms of per‐residue predicted local‐distance difference test (pLDDT) confidence scores, AlphaFold2 models of class II hydrophobins generally exhibit higher per‐residue pLDDT values (indicating greater confidence) relative to class I hydrophobins, consistent with the enhanced structural diversity, sequence diversity, and flexibility of class I proteins. There were some notable deficiencies in AlphaFold's ability to model the loop regions for some of the hydrophobins (for example, it does well for PC1, HFBI, and HFBII, but poorly for DewA, EAS, and NC2) (Figure 3b,c). Difficulty in modeling the flexible loops of hydrophobins is an important potential limitation, given that the C7‐C8 loop of class I (Kwan et al., 2006; Kwan et al., 2008; Macindoe et al., 2012) and C4‐C5 / C7‐C8 loops of class II hydrophobins (Gallo et al., 2023; Lienemann et al., 2013; Valsecchi et al., 2019) are important for self‐assembly. Both NMR dynamic measurements and MD simulations suggest these loops are highly flexible and unlikely to adopt a single well‐ordered conformation for monomeric hydrophobins in solution (Kwan et al., 2008; Mackay et al., 2001; Nolle et al., 2023; Vergunst & Langelaan, 2022). Other investigators have observed that AlphaFold assigns lower pLDDT values to disordered regions of proteins with high sequence diversity (Akdel et al., 2022). Together, these results reveal that AlphaFold2 seems to predict structures of the monomeric states of both class I and class II hydrophobins with moderate to high confidence, but may have some limitations in modeling the flexible, functional loops of hydrophobins.

### 3.5 AlphaFold models enable global classification of the structural universe of class I and class II hydrophobins

**FIGURE 4:**
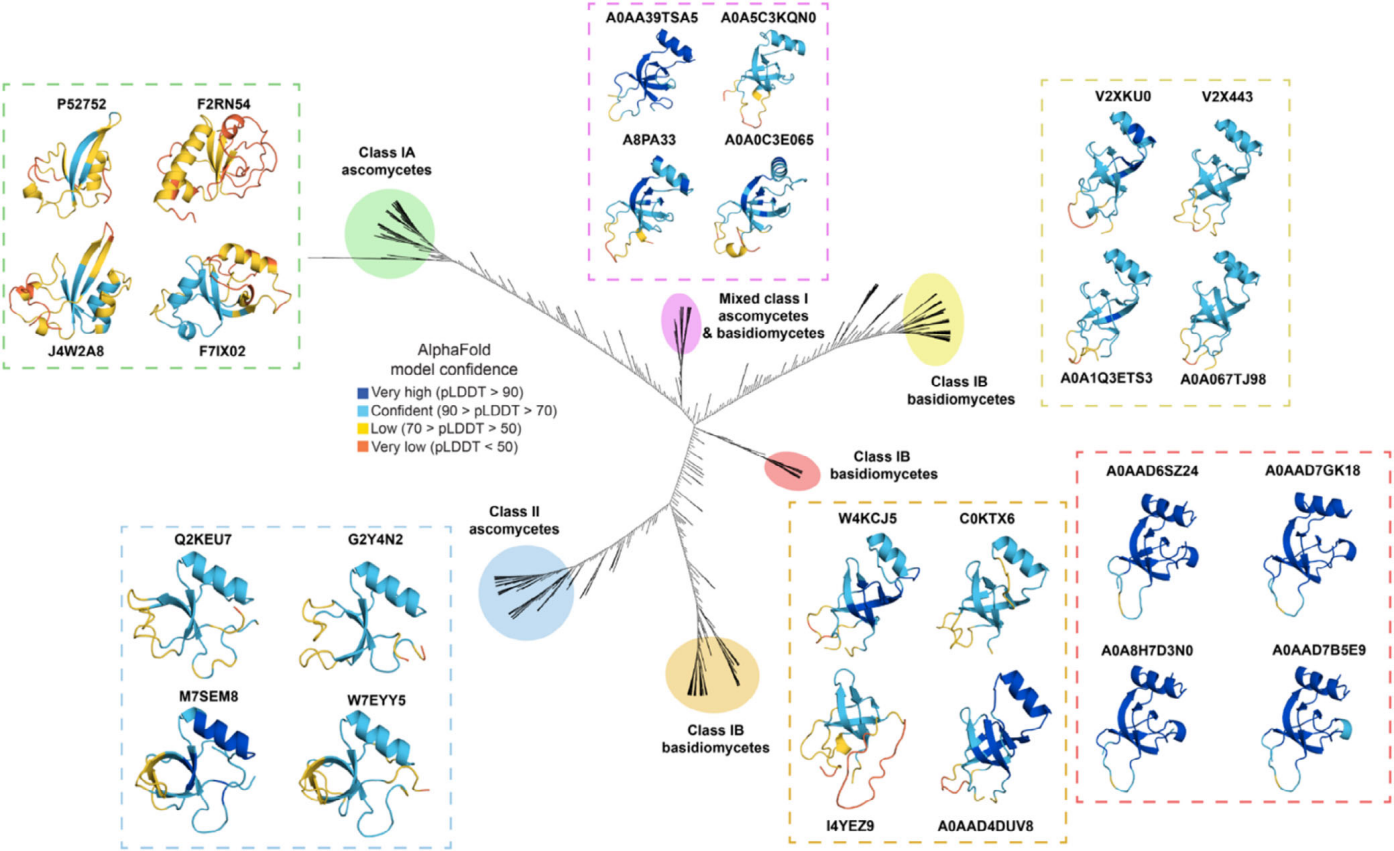
Structure‐based dendrogram of AlphaFold2 models classifies class I and class II hydrophobins into six distinct clades. Global comparison of AlphaFold2 models was performed with multiple structure alignment software FoldMason (Gilchrist et al., 2024). An unrooted structure‐based dendrogram was generated by iTOL, resulting in 6,920 leaves (i.e., structural models) broken up into six main clades highlighted with different colored circles (Letunic & Bork, 2024). Representative AlphaFold2 models are shown for each clade colored per‐residue by pLDDT score. The flexible N‐terminal tails are not shown for clarity. Each structure is labeled with its corresponding UniProt accession number. Class I hydrophobins are subdivided into class IA and class IB based on ascomycetes or basidiomycetes origins, respectively.

To uncover global structural features for class I and class II hydrophobins, we compared AlphaFold models of all class I and class II hydrophobins using the multiple structure alignment tool FoldMason (Gilchrist et al., 2024). We then prepared an unrooted structure‐based dendrogram using the iTOL tool (Letunic & Bork, 2024). The dendrogram highlights the impressive structural diversity of class I hydrophobins with five unique clades showing clear separation in the tree (Figure 4). For example, as previously noted from phylogenetic analysis of hydrophobin sequences (Gandier et al., 2017; Linder et al., 2005; Tanaka et al., 2022), global structural comparisons resulted in the subdivision of class I hydrophobin sequences into those originating from Ascomycota or Basidiomycota fungal phyla (class IA and class IB, respectively) (Figure 4). Class IA differed from Class IB due to subtle differences in the central anti‐parallel β‐sheet “core” structure. Class IB proteins originating from Basidiomycota were further split into three similar but unique clades that exhibit differences in the central β‐barrel structure and intercysteine loops (Figure 4). Structures within intermediate/mixed folds were also identified as a unique clade (Figure 4) (Jensen et al., 2010). In stark contrast, class II hydrophobins were localized into a single clade originating from Ascomycota, consistent with high structural conservation (Figures 1 and 4) (Hakanpää et al., 2004; Ren et al., 2014). To gain insights into how hydrophobin sequence conservation is linked to structure conservation, we generated a sequence‐based dendrogram of all hydrophobins. In contrast to the six well‐defined clades of the structure‐based dendrogram, the sequence‐based dendrogram had many more subclades despite clear disambiguation between class IA, class IB, and class II hydrophobin classes (Figure 4, Supplementary Figure S7). This suggests much greater hydrophobin sequence diversity relative to structural diversity. Sequence alignments showed that class II ascomycetes contained high conservation for the canonical cysteine residues, core residues in the β‐barrel structure, the C3‐C4 loop, and the C7‐C8 loop but limited conservation in the C1‐C2 loop, C4‐C5 loop, and α1 helix (Supplementary Figure S8). Conserved residues in class IA and class IIB hydrophobins were primarily localized to the canonical cysteine residues and core residues in the β‐barrel structure with low conservation in the C3‐C4 loop and C4‐5 loop (Supplementary Figure S8). The C7‐C8 showed some sequence conservation for class IB but not class IA. Together, these results suggest that AlphaFold models can be used toward global classification of class I and class II hydrophobins into six distinct clades that exhibit unique sequence and structure features.

### 3.6 AlphaFold models enable identification of non‐canonical hydrophobin features

**FIGURE 5:**
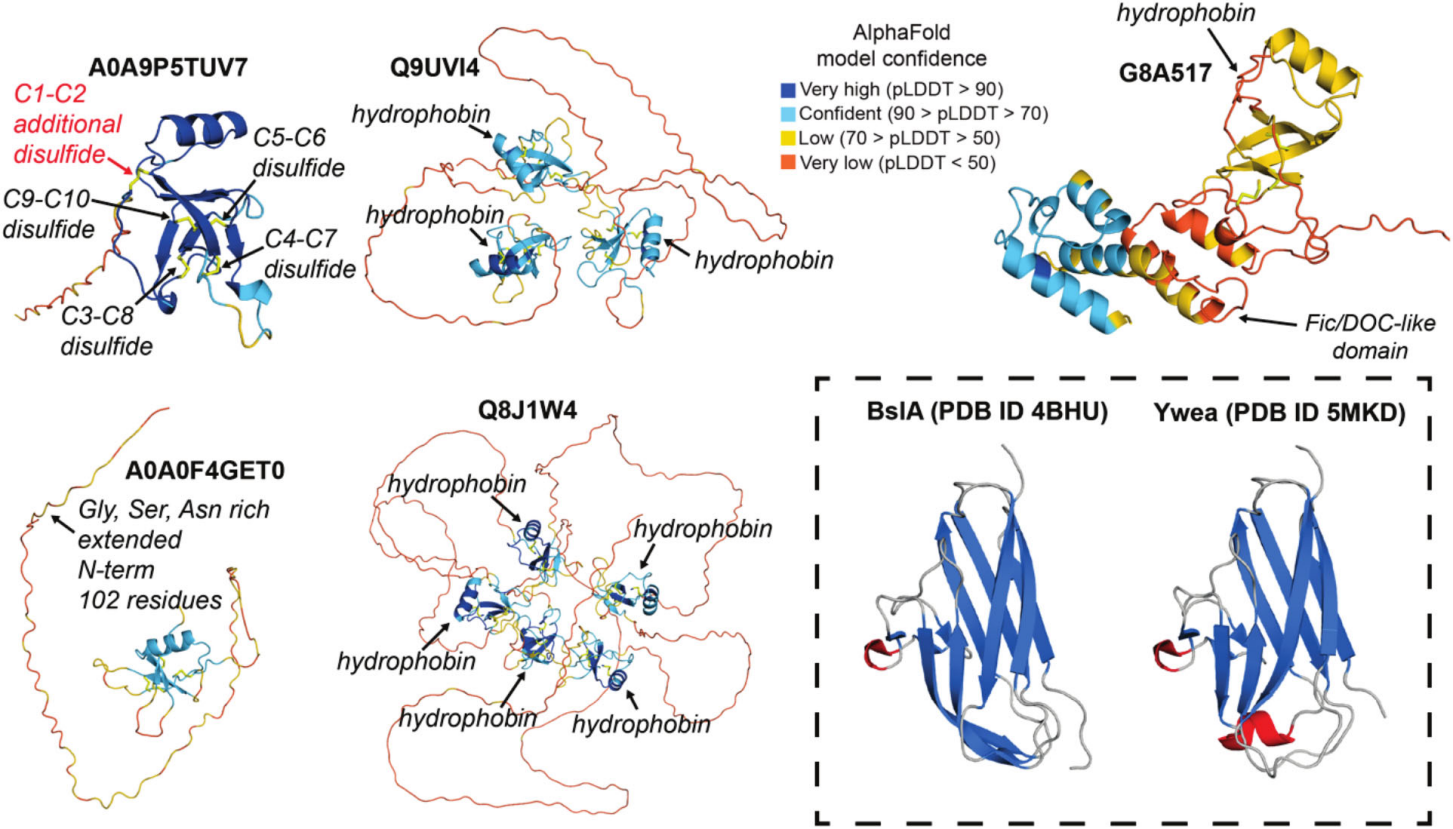
AlphaFold models reveal non‐canonical features of hydrophobins. An AlphaFold3 model of UniProt ID A0A9P5TUV7 (a class I hydrophobin from *Rhodocollybia butyracea* with five disulfide bonds—note that the nomenclature of the Cys pairs is different from the canonical class I hydrophobins). AlphaFold Protein Structure Database accession numbers are: AF‐A0A0F4GET0‐F1 (a class II hydrophobin from *Zymoseptoria brevis* with a Gly, Ser, Asn rich extended disordered N‐terminal domain), AF‐Q9UVI4‐F1 (a class II trihydrophobin TH1 from *Claviceps fusiformis*), AF‐Q8J1W4‐F1 (a class II pentahydrophobin CPPH1 from *Claviceps purpurea*), and AF‐G8A517‐F1 (a class I hydrophobin from *Flammulina velutipes* that contains a Fic/DOC‐like domain). The dotted box highlights Protein Data Bank accession numbers: PDB ID 4BHU (bacterial hydrophobin BslA from *Bacillus subtilis*) (Hobley et al., 2013) and PDB ID 5MKD (bacterial hydrophobin Ywea from *Bacillus subtilis*) (Arnaouteli et al., 2023). AlphaFold models are colored by per‐residue pLDDT score.

With AlphaFold models in hand, we searched for non‐canonical hydrophobin features that could result in unique functional properties. Several putative non‐canonical features are described below and illustrated in Figure 5. First, while canonical hydrophobins contain a cysteine count of 8 resulting in four disulfide bonds (Supplementary Figure S4b), we identified hydrophobins with 10 cysteines that were predicted by AlphaFold to have an additional fifth oxidized disulfide bond. An example is UniProt ID #A0A9P5TUV7, a putative class I hydrophobin from *Rhodocollybia butyracea* (Figure 5). A total of 112 class I and 17 class II hydrophobins with 10 cysteines were identified, suggesting hydrophobins with 10 cysteines are relatively rare. The class I sequences with 10 cysteines were biased toward *Peniophora, Collybiopsis, Armillaria*, and *Talaromyces* fungal genera. The class II sequences with 10 cysteines were biased toward *Tolypocladium, Fusarium, Akanthomyces*, and *Phomopsis* fungal genera. For class II hydrophobins, the new disulfide bond in UniProt ID #A0A8K0J0P6 could be primitively termed the C9‐C10 disulfide since it precedes the canonical C7‐C8 disulfide and is spatially placed adjacent to the core hydrophobin fold (Supplementary Figure S9). The structure and sequence for A0A8K0J0P6 were partially conserved across the canonical class II hydrophobins HFBI, HFBII, and NC2 such that we expect it to be functionally active. For class I hydrophobins, despite the almost perfect spatial overlap of the four canonical disulfide bonds with four of the disulfide bonds for the putative novel hydrophobin, the sequence mapping of Cys connectivity was different (Supplementary Figure S10 – for example, the canonical C1‐C6 disulfide connectivity maps to the new C4‐C7 disulfide connectivity). Given these differences between class I hydrophobins with 8 and 10 cysteines, we predicted the aggregation‐prone sequence of non‐canonical class I hydrophobins with five disulfide bonds relative to canonical class I hydrophobins using the in silico tool AggreProt (Planas‐Iglesias et al., 2024). AggreProt correctly identified the C7‐C8 loop as the likely aggregation site for canonical class I hydrophobins EAS, MPG1, and SC16 (Supplementary Figure S11). For non‐canonical class I hydrophobins A0A9P5TUV7, A0A5E3WUH3, A0A5E3X438, a sequence spanning C7 to C10 was identified (Supplementary Figure S11 – note that here the Cys residue numbering is different as described above). Finally, the non‐canonical class I hydrophobins exhibited an amphipathic nature with one hydrophobin and one hydrophilic surface, suggesting that they are bona fide hydrophobins (Supplementary Figure S11). The ability of these new putative hydrophobins to self‐assemble into functional rodlets remains to be shown experimentally. Thus, taken with a grain of salt, the putative new class I hydrophobins with five disulfide bonds seem to exhibit unique sequence features despite an overall EAS‐like hydrophobin fold. AlphaFold placed the new disulfide bond spatially near the canonical C3‐C4 disulfide (for class I) and the canonical C1‐C6 disulfide (for class II) where it could impact the folding properties, stability, or aggregation features of the hydrophobin β‐barrel core (Figure 5, Supplementary Figures S9–S11).

Second, we identified hydrophobins with an extended, disordered N‐terminal tail (independent of the secretion signal). On average, canonical hydrophobins contain a 15 to 25 residue long signal peptide followed by a few (<25) disordered residues before the beginning of the central β‐barrel core. We defined non‐canonical hydrophobins with extended disordered N‐terminal tails to have at least 70 residues preceding the first Cys in the sequence, which forms the boundary of the β‐barrel core (Supplementary Figure S12a). An example is UniProt ID #A0A0F4GET0, a putative class II hydrophobin from *Zymoseptoria brevis* (Figure 5). Many extended N‐terminal tails were predicted by AlphaFold to be primarily disordered, although low confidence secondary structure elements were modeled within some sequences (Supplementary Figure S12b). A total of 143 class I and 60 class II hydrophobins with extended N‐terminal tails were identified. The class I sequences with extended N‐terminal tails were biased toward *Armillaria, Aspergillus*, and *Rhizoctonia* fungal genera. The class II sequences with extended N‐terminal tails were biased toward *Trichoderma, Cercospora*, and *Ophiocordyceps* fungal genera. Amino acid sequence alignment revealed that, in general, beyond the signal peptide, there is limited conservation of residues within the extended N‐terminal tail for both class I and class II hydrophobins (Supplementary Alignments S1 and S2). However, apart from the extended disordered N‐terminal tail, there was more structural and sequence similarity within the β‐barrel core of the hydrophobin domain, suggesting hydrophobins with extended N‐terminal tails are fully functional proteins (Supplementary Figure S12b, Supplementary Alignments S1 and S2). The biological function of the disordered N‐terminal tail is poorly understood but it does not seem to be strictly required for self‐assembly (Vergunst & Langelaan, 2022). Long, disordered N‐terminal tails could perform regulatory functions analogous to those observed for other proteins: they could regulate conformational changes via allosteric coupling effects (Tee et al., 2020), act as intramolecular entropic bristles limiting undesired protein aggregation (Graña‐Montes et al., 2014; Yu & Sukenik, 2023), or provide binding sites for interactions with modulatory proteins (Převorovský et al., 2011). We found that non‐canonical class II (and to a lesser extent class I) hydrophobins with extended N‐terminal sequences were rich in Gly, Ser, and Asn residues that promote phase separation (Ibrahim et al., 2023) (Supplementary Alignment S1 and S2). The potential for the non‐canonical hydrophobins with extended N‐terminal tails to undergo phase separation was predicted with the *in silico* tool ParSe (Ibrahim et al., 2023), which supported that extended N‐terminal hydrophobin sequences likely contain features for phase separation (Supplementary Figure S13). Taken together, these results allow us to hypothesize that some non‐canonical hydrophobins with extended disordered N‐terminal tails might have the ability to undergo phase separation that promotes or alters their functionality.

Third, we identified polyhydrophobin sequences containing three (trihydrophobin) to five (pentahydrophobin) hydrophobin domains within a single polypeptide chain. Examples include UniProt ID #Q9UVI4, a class II trihydrophobin CFTH1 from *Claviceps fusiformis*, and #Q8J1W4, a class II pentahydrophobin CPPH1 from *Claviceps purpurea* (Figure 5). A total of 12 class I (11 trihydrophobin, 1 pentahydrophobin) and 126 class II (124 trihydrophobin, 2 pentahydrophobin) multidomain hydrophobins were identified. The class I sequences with multidomain hydrophobins were biased toward *Fusarium* fungal genera. The class II sequences with multidomain hydrophobins were biased toward *Fusarium, Trichoderma, Gibberella*, and *Claviceps* fungal genera. As highlighted for CPPH1 from *Claviceps purpurea*, a more in‐depth analysis shows that the individual hydrophobin units of the class II pentahydrophobin were highly conserved in both sequence and structure with canonical monomeric hydrophobins, such as HFBII (Supplementary Figure S14a–d). Thus, we expect that multidomain hydrophobins can self‐assemble, although how the multimerization could influence the assembly process or monolayer structures remains to be seen. These findings are consistent with previous reports that have identified and characterized trihydrophobins and pentahydrophobins, rich in Gly/Asp repeat linkers, as functional self‐assembling hydrophobins from *Claviceps* with potential roles in the formation of aerial hyphae (De Vries et al., 1999; Mey et al., 2003). Future studies should evaluate the potential of fungal proteases to cleave polyhydrophobins into monomeric hydrophobins.

Finally, although not present within our fungal hydrophobin testing set, non‐canonical bacterial hydrophobins, such as BslA and Ywea, have been reported (Arnaouteli et al., 2023; Hobley et al., 2013). BslA and Ywea adopt an immunoglobulin‐like β‐sandwich fold, similar to the amyloid‐forming protein β2‐microglobulin (Wilkinson et al., 2023). AlphaFold was able to predict bacterial hydrophobin folds with moderate to high accuracy within the immunoglobulin‐like core; however, notable differences were observed in the cap region of the bacterial hydrophobins that are reported to be important for assembly (Arnaouteli et al., 2023; Hobley et al., 2013) (Supplementary Figure S15).

Together, these results highlight that AlphaFold models of hydrophobins, together with sequence analysis and *in silico* functional prediction tools, can reveal putative non‐canonical features and have the potential to generate tested hypotheses to guide future *in vitro, in situ*, and *in vitro* experiments to disambiguate their functionalities.

### 3.7 AlphaFold models uncover hydrophobin‐like domains within non‐hydrophobin proteins

We also identified hydrophobin domains within a single polypeptide chain that harbored separate well‐folded globular domains outside of the primary hydrophobin fold. An example is UniProt ID #G8A517, a class I hydrophobin from *Flammulina velutipes* that contains a Fic/DOC‐like domain (Figure 5). Although it remains to be experimentally tested, the presence of the Fic/DOC‐like domain could promote AMPylation of proteins at hydrophobic‐hydrophobic surfaces where the hydrophobin self‐assembles (Veyron et al., 2018). Intrigued by the possibility of non‐hydrophobin domains covalently linked in a single polypeptide chain to hydrophobin‐like domains, we used canonical hydrophobin structures as “bait” proteins within Foldseek, which searches several structural databases (AlphaFold/Proteome, AlphaFold/Swiss‐Prot, AlphaFold/UniProt50, CATH50, GMGCL MGnify‐ESM30, PDB100) to identify non‐hydrophobin proteins that contain domains resembling class I or class II hydrophobins (van Kempen et al., 2024). Many canonical hydrophobins were identified but not considered for analysis since the goal was to identify hydrophobin‐like folds within other proteins. Instead, hits were defined solely as structures that contained separate well‐folded globular domains in addition to a canonical hydrophobin‐like fold.

**FIGURE 6:**
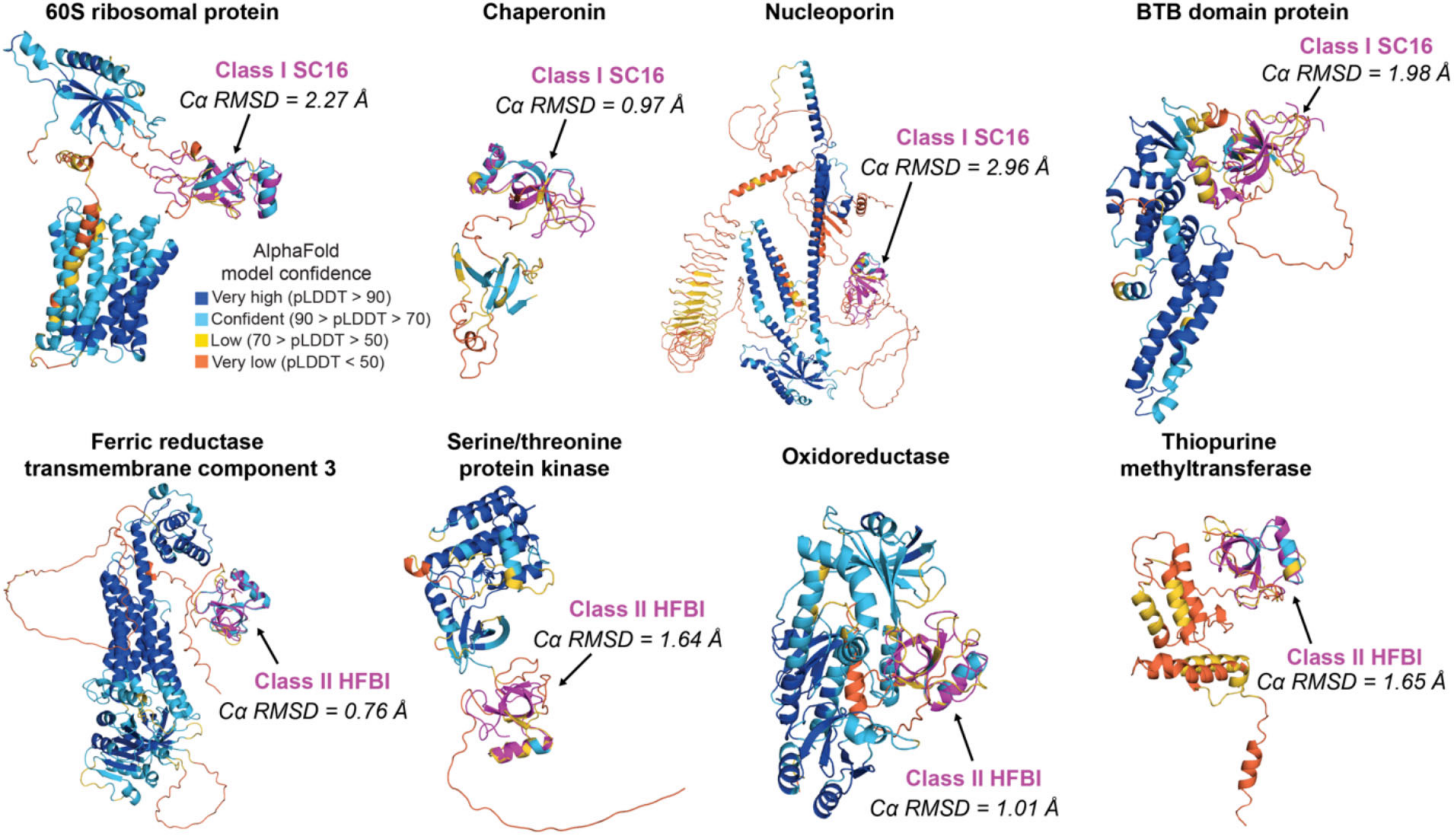
AlphaFold models uncover hydrophobin‐like domains within non‐hydrophobin proteins. Comparison of experimental hydrophobin structures (magenta) overlaid with AlphaFold models of proteins containing hydrophobin‐like domains (colored by pLDDT score). Examples for proteins containing class I hydrophobin‐like domains: AF‐A0A137QB05‐F1 (60S ribosomal protein L27‐A from *Leucoagaricus* sp.), AF‐A0A0J5PMS5‐F1 (Chaperonin from *Aspergillus fumigatus*), AlphaFold3 model of UniProt ID A0A8H5FVP6 (Nucleoporin Nup54 from *Leucoagaricus leucothites*), and AF‐A0A0W0FA47‐F1 (BTB domain‐containing protein from *Moniliophthora roreri*). Examples for proteins containing class II hydrophobin‐like domains: AF‐A0A0A2W2K4‐F1 (Ferric reductase transmembrane component 3 from *Beauveria bassiana*), A0A162IG95‐F1 (serine/threonine protein kinase BUD32 from *Sporothrix insectorum*), AF‐A0A4S9H610‐F1 (2‐polyprenyl‐6‐methoxyphenol hydroxylase‐like oxidoreductase from *Aureobasidium pullulans*), and AF‐A0A2W1CXJ2‐F1 (Thiopurine methyltransferase from *Pyrenophora tritici‐repentis*). Cα RMSD values (Ångströms, Å) each hydrophobin relative to the hydrophobin‐like domain are noted. Reference hydrophobins are colored magenta: SC16 for class I (PDB ID 2NBH) and HFBI (PDB ID 2FZ6) for class II. AlphaFold models are colored by per‐residue pLDDT score.

For class II, 25 total hits were identified using HFBI, HFBII, and NC2 structures as bait (Supplementary Figure S16). For class I, 62 total hits were identified using DewA, EAS, MPG1, PC1, RodA, SC16, SL14, and WI1 structures as bait (Supplementary Figure S16). Notably, most hit proteins were derived from fungal organisms (i.e., *Leucoagaricus* sp. and *Aspergillus* sp.). However, a few hits were derived from non‐fungal organisms (i.e., dinoflagellate symbiotic alga *Zooxanthella*, diatom *Skeletonema*, and plant *Brassica*). Many hits were shared and consistently identified across the similar bait proteins, but there were also unique hits from some bait proteins (Supplementary Figure S16). The hydrophobin‐like domains had Cα RMSD values ranging from 0.5 to 5 Å to the bait class I or class II hydrophobin structures, indicating high to low structural similarity. Examples of hits are described below. Using SC16 as the bait for class I hydrophobin‐like domains, we identified a putative 60S ribosomal protein L27‐A from *Leucoagaricus* sp. (UniProt #A0A137QB05), a putative Chaperonin from *Aspergillus fumigatus* (UniProt #A0A0J5PMS5), a putative Nucleoporin from Nup54 from *Leucoagaricus leucothites* (UniProt #A0A8H5FVP6), and a putative BTB domain‐containing protein from *Moniliophthora roreri* (UniProt #A0A0W0FA47) (Figure 6, Supplementary Figure S17 for other examples). Using HFBI as the bait for class II hydrophobin‐like domains, we identified a putative Ferric reductase transmembrane component 3 from *Beauveria bassiana* (UniProt #A0A0A2W2K4), a putative serine/threonine protein kinase BUD32 from *Sporothrix insectorum* (UniProt #A0A162IG95), a putative 2‐polyprenyl‐6‐methoxyphenol hydroxylase‐like oxidoreductase from *Aureobasidium pullulans* (UniProt #A0A4S9H610), a putative Thiopurine methyltransferase from *Pyrenophora tritici‐repentis* (UniProt #A0A2W1CXJ2) (Figure 6, Supplementary Figure S18 for other examples). In general, the hydrophobin‐like domains from polypeptide chains within other proteins were found to be well conserved in both sequence and structure with monomeric class I and class II hydrophobins. For example, most of the identified hydrophobin‐like domains contained the four intramolecular disulfide bonds and intercysteine loops that resembled canonical hydrophobins, suggesting many of the newly identified non‐canonical hydrophobins might be functional for self‐assembly (Supplementary Figures S19 and S20). One caveat of this analysis is that many of the non‐canonical hydrophobin‐like domains had moderate pLDDT values (50–70), suggesting only some confidence in the predicted hydrophobin fold. We suspect this may be due to limitations in sequence coverage and/or template identity in the multiple sequence alignment (MSA). This is supported by heat‐map representations of the MSA coverage and plots of predicted lDDT per‐residue position for a hydrophobin domain with low average pLDDT (UniProt #A0A162IG95) and high average pLDDT (UniProt #A0A2H1FZK2) (Supplementary Figure S21).

Together, these results suggest a combination of databases containing AlphaFold models (the AlphaFold structure database) and robust structural alignment software (Foldseek) is useful at identifying novel putative proteins with hydrophobin‐like domains. The role of the hydrophobin‐like domain in these putative proteins is unclear and requires experimental testing. We posit that the hydrophobin‐like domains either help localize these proteins at the fungal membrane‐water surface or promote oligomerization.

### 3.8 Limitations in AlphaFold and Chai‐1 modeling of hydrophobin self‐assembly

Another outstanding question is whether AlphaFold3 and other machine‐learning‐based structure prediction software, such as Chai‐1, can generate plausible models of hydrophobin binding to membranes and/or hydrophobin self‐assembly (Abramson et al., 2024; Discovery et al., 2024; Evans et al., 2022; Jumper et al., 2021; Krishna et al., 2024). To address this, we performed AlphaFold3 modeling of class I EAS_Δ15_ and class II HFBII in three different scenarios: (i) 8 hydrophobin copies in the absence of a membrane‐mimetic; (ii) 1 copy of hydrophobin with 50 copies of oleic acid; and (iii) 8 hydrophobin copies with 50 copies of oleic acid. Oleic acid was used to mimic a membrane‐like hydrophobic surface. Relative to the experimental structure of class I EAS_Δ15_ (Kwan et al., 2008; Macindoe et al., 2012), AlphaFold3 predicted conformational changes in the C3‐C4 and C7‐C8 functional loops in the presence of oleic acid or multiple hydrophobin copies (Supplementary Figure S22a–c). Further, the hydrophobic surface of EAS was embedded into the oleic acid membrane, with the hydrophilic surface providing contacts between hydrophobin monomers. Even after running calculations with more than 100 different model seeds, AlphaFold3 was not able to predict the amyloid‐like fibril structure expected of EAS_Δ15_ (Kwan et al., 2008; Macindoe et al., 2012). Relative to the experimental structure of class II HFBII (Hakanpää et al., 2004; Kallio et al., 2007; Linder et al., 2001), AlphaFold3 did not predict conformational changes in the C3‐C4 and C7‐C8 functional loops of HFBII in the presence of oleic acid or multiple hydrophobin copies (Supplementary Figure S23a–c). The hydrophobic surface of HFBII was also embedded into the membrane, with the hydrophilic surface providing contacts between hydrophobin monomers. AlphaFold did not predict the mesh‐like structure expected of HFBII, nor did it predict oligomerization mechanisms as observed in the crystal structure of HFBII in the presence of detergents (Supplementary Figure S23d) (Hakanpää et al., 2004; Kallio et al., 2007; Linder et al., 2001). We also attempted to force the amyloid‐like fibril structure of class I EAS_Δ15_ by performing structure modeling in Chai‐1 using distance restraints between TNT (residues 52 to 54 based on numbering in PDB 2K6A) and FLI (residues 58 to 60 based on numbering in PDB 2K6A) (Kwan et al., 2008; Macindoe et al., 2012). Relative to the experimental structure of EAS_Δ15_ (Kwan et al., 2008; Macindoe et al., 2012), Chai‐1 models with distance restraints showed a massive conformational change in the C3‐C4 and C7‐C8 functional loops with increased β‐sheet content but still did not resemble the expected amyloid‐like fibril structure (Supplementary Figure S24). Taken together, these results highlight that AlphaFold and Chai‐1 models of hydrophobins have the potential to provide insights into binding of hydrophobic surfaces but have difficulty modeling amyloid‐like fibrils and mesh‐like oligomeric states. These limitations of modern structure prediction software have been reported for other systems (Pinheiro et al., 2021).

## 4 DISCUSSION

Here, we apply a suite of computational tools with AlphaFold models at the helm to analyze, model, classify, and compare the global features of fungal class I and class II hydrophobins. To our knowledge, this work represents the most comprehensive application of these bioinformatics tools to date for the exploration of structure–function relationships in hydrophobins. Based on quantitative analysis of molecular interactions within the *Rosetta* REF2015 forcefield, both class I and class II hydrophobins are stabilized by attractive forces (representative of hydrophobic interactions), electrostatics (representative of dipole interactions and charged residue interactions), and backbone hydrogen bonding; these forces also stabilize other small, globular proteins (Figure 2, Supplementary Figure S3). The consistency between the results is striking given the sequence and structural diversity within the class I hydrophobin family and differences between class I and class II folds. Nonetheless, in each case, the residue‐residue pairs defining the total energy are highly located within the core β‐sheet. These findings set a baseline for future *Rosetta* analysis that could be useful for defining how mutations destabilize monomeric hydrophobin states (i.e., ∆∆G of mutation via *Rosetta*'s *cartesian_ddg* application) (Park et al., 2016) or using computational site‐directed mutagenesis to engineer tunable features into hydrophobins (i.e., *Rosetta*'s mutagenesis protocol, Rosetta design) (Thieker et al., 2022).

While AlphaFold has revolutionized the structure prediction field, it is not assumed that it will perform well at every modeling task. Benchmarking of different families of proteins is essential to understand the strengths and weaknesses of AlphaFold and its algorithmic cousins (Agarwal & McShan, 2024; Akdel et al., 2022; Chakravarty & Porter, 2022). Our benchmark shows that AlphaFold is robust at predicting structures of monomeric class I and class II hydrophobins but may have some limitations in modeling the functional intercysteine loops (Figure 3). It may be possible to combine AlphaFold modeling with *Rosetta* relax protocols or all‐atom MD simulations to evaluate the conformational landscapes of loops with lower pLDDT values. AlphaFold's training procedure involved a self‐distillation set together with known structures from the PDB (maximum release date of 30 April 2018) (Jumper et al., 2021). Furthermore, AlphaFold was not trained on structures solved by NMR methods (Abramson et al., 2024; Jumper et al., 2021). This matters here because many hydrophobin structures in the PDB were determined by NMR (Supplementary Table S1). Since many hydrophobin structures evaluated in our benchmark were either released after the training or are solution NMR structures, our results suggest that AlphaFold has not simply learned to predict the same structures it was trained on but is a bona fide predictor of monomeric class I and class II hydrophobin folds. As new hydrophobin structures are determined, these conclusions should be further evaluated.

Classification of hydrophobins is important because each subclass exhibits unique functional features (i.e., rodlet formation vs. mesh formation), and comparisons between classes can provide information on evolutionary mechanisms (Ren et al., 2013; Sunde et al., 2008; Wösten, 2001; Wösten & de Vocht, 2000). Class I and class II hydrophobins have been historically analyzed and classified by sequence‐based phylogenetic analysis (Gandier et al., 2017; Jensen et al., 2010; Linder et al., 2005; Whiteford & Spanu, 2002). Here, a combination of AlphaFold2 modeling and multiple structure alignment at scale by FoldMason (Gilchrist et al., 2024) allowed us to construct a detailed unrooted dendrogram of hydrophobins based on structural comparison. Six distinct clades were identified: one for class IA ascomycetes, one for class II ascomycetes, one mixed clade for ascomycetes and basidiomycetes, and three separate clades for class IB basidiomycetes (Figure 4). Together, these clades represent new insights into global structural features of class I and class II fungal hydrophobins structural universe. Representative structures highlight an interesting dichotomy: while the hydrophobin core and loops are quite diverse, the disulfide bonded β‐sheet core is an underlying commonality where subtle differences could fine‐tune protein stability and dynamics. We expect that upon identification of new hydrophobin sequences, AlphaFold modeling, and together with FoldMason, can provide a robust, structure‐based classification scheme. Finally, structure‐based and sequence‐based dendrograms can provide useful information on how hydrophobins have evolved in fungi. For example, it has been proposed that class II hydrophobins evolved independently of class I hydrophobins in a case of convergent evolution (Whiteford & Spanu, 2002). Class IA hydrophobins and some clades of class IB hydrophobins showed a very similar core β‐barrel/α‐helix fold typical of hydrophobins despite being in separate evolutionary clades with distinct sequence features (Figure 4, Supplementary Figures S7 and S8). The strong functional selection for maintaining a specific architecture, even if sequences diverge widely, implies that similar structures have evolved independently in distant fungal groups to perform similar roles, which is a hallmark of convergent evolution.

Through analysis of AlphaFold models and the application of protein structure alignment at scale with Foldseek (van Kempen et al., 2024), we also identified relatively rare non‐canonical features of hydrophobins: hydrophobins with extended N‐terminal tails, hydrophobins with five disulfide bonds, polyhydrophobins, and non‐hydrophobin proteins containing hydrophobin‐like folds (Figures 5 and 6). While some of these features are supported by previous experiments (i.e., trihydrophobins and pentahydrophobins) (De Vries et al., 1999; Mey et al., 2003), many of these putative features or hypothetical proteins remain to be tested. Our data allow us to posit the following: extended N‐terminal tails may be involved in phase separation (Supplementary Figure S13); the additional disulfide bond could impart increased stability into the hydrophobin fold (Supplementary Figures S9 and S10); and the possibility exists that the presence of hydrophobin‐like folds within diverse families of fungal proteins could influence their biological function or activity in meaningful ways (Supplementary Figures S17 and S18). In particular, the identification and characterization of hydrophobin domains in a single polypeptide chain with non‐hydrophobin domains could inform the design of many types of bifunctional proteins for a myriad of applications. The emergence of new copies of hydrophobin folds within other proteins in some cases could arise from gene duplication events that have been proposed in many hydrophobin‐containing fungal species (Karlsson et al., 2007; Kubicek et al., 2008; Mgbeahuruike et al., 2013).

Several important areas of structure–function relationships of hydrophobins have not been probed here but should be addressed in future work. The first is that AlphaFold and Chai modeling of hydrophobin self‐assembly and membrane binding seems to be a major limitation (Supplementary Figures S22–S24). In addition, hydrophobins are highly dynamic proteins that sample conformationally diverse ensembles in solution (Macindoe et al., 2012; Mackay et al., 2001; Vergunst & Langelaan, 2022). The “static” structures analyzed and modeled here provide useful insights but may not represent the full picture. Several groups are working on adapting AlphaFold toward prediction of conformational ensembles by clustering or subsampling of the multiple sequence alignments (Bowman, 2024; Monteiro da Silva et al., 2024; Sala et al., 2023; Wayment‐Steele et al., 2024). Once these algorithms are refined and benchmarked to address a wide range of systems, it will be interesting to see them applied to class I and class II hydrophobins. The modeling is expected to be improved by the direct integration of structure prediction tools with experimental measurements of dynamics through the use of NMR spectroscopy, neutron spin echo spectroscopy, or small angle neutron scattering (Chinnam et al., 2023; Huang & Montelione, 2024; Stingaciu, 2022). For future studies, several approaches that have helped for other amyloid‐like assemblies may prove useful (Ragonis‐Bachar et al., 2024; Wojciechowska et al., 2024), (i) performing thousands of different predictions with randomized seeds followed by sorting based on ipTM score (Abramson et al., 2024), (ii) performing predictions with varying chain copy numbers, (iii) including different types of lipids in the prediction to generate an artificial hydrophobic–hydrophilic interface (Kallio et al., 2007), and (iv) providing explicit residue‐residue restraints from experimental mutagenesis (Gallo et al., 2023; Kwan et al., 2006; Kwan et al., 2008; Lienemann et al., 2013; Valsecchi et al., 2019). Conducted against a backdrop of steadily improving computational tools and with these limitations in mind, AlphaFold seems ready to uncover new biology, provide new testable hypotheses, and aid in the engineering of proteins.

## AUTHOR CONTRIBUTIONS

**Li‐Yen Yang:** Investigation. **Daniel J. Hicks:** Conceptualization; investigation. **Paul S. Russo:** Writing – review and editing; supervision. **Andrew C. McShan:** Conceptualization; investigation; writing – original draft; writing – review and editing; methodology; formal analysis; supervision; visualization.

## CONFLICT OF INTEREST STATEMENT

The authors declare that there are no competing interests.

## Supporting information


**Data S1.** Supporting Information.

## Data Availability

All AlphaFold models, scripts used to generate the data, and source data are freely provided on GitHub at https://github.com/mcshanlab/Yang_hydrophobins_2025.
