# Supplementary material for: AlphaFold modeling uncovers global structural features of class I and class II fungal hydrophobins
Source: Protein Sci. 2025 Aug 25;34(9):e70279. doi: 10.1002/pro.70279 (PMC12375992; doi:10.1002/pro.70279)

**Supplementary Material**

**Supplementary Table 1. Summary of fungal class I and class II hydrophobins and bacterial hydrophobin structures in the Protein Data Bank**

| Name | Species | Class | PDB ID | Type | Notes | Reference |
| --- | --- | --- | --- | --- | --- | --- |
| HFBII | *T. reesei* | Class II | 1R2M | X-ray |  | ^42^ |
| HFBII | *T. reesei* | Class II | 2B97 | X-ray | high res | ^46^ |
| EAS | *N. crassa* | Class I | 2FMC | NMR |  | ^45^ |
| HFBI | *T. reesei* | Class II | 2FZ6 | X-ray |  | ^27^ |
| HFBI | *T. reesei* | Class II | 2GVM | X-ray | w/ LDAO detergent | ^27^ |
| EAS | *N. crassa* | Class I | 2K6A | NMR | truncation mutant | ^159^ |
| EAS | *N. crassa* | Class I | 2LFN | NMR |  | ^28^ |
| DewA | *A. nidulans* | Class I | 2LSH | NMR |  | ^49^ |
| MPG1 | *M. oryzae* | Class I | 2N4O | NMR |  | ^158^ |
| SC16/HYD1 | *S. commune* | Class I | 2NBH | NMR |  | ^34^ |
| HFBII | *T. reesei* | Class II | 2PL6 | X-ray | w/ heptyl-β-d-thioglycoside detergent | ^57^ |
| HFBII | *T. reesei* | Class II | 2PL7 | X-ray | w/ heptyl-β-d-thioglycoside detergent | ^57^ |
| HFBII | *T. reesei* | Class II | 3QQT | X-ray | in presence of polystyrene nanospheres | ^70^ |
| NC2 | *N. crassa* | Class II | 4AOG | NMR |  | ^67^ |
| NC2 | *N. crassa* | Class II | 4BWH | NMR |  | ^67^ |
| SLH4 | *S. lacrymans* | Class I | 5W0Y | NMR |  | ^160^ |
| WI1 | *W. ichthyophaga* | Class I | 6E9M | NMR |  | ^35^ |
| PC1 | *P. carnosa* | Class I | 6E98 | NMR |  | ^35^ |
| RodA | *A. fumigatus* | Class I | 6GCJ | NMR |  | ^72^ |
| SC16/HYD1 | *S. commune* | Class I | 7S7S | X-ray | P21212 space group | ^65^ |
| SC16/HYD1 | *S. commune* | Class I | 7S86 | X-ray | C2221 space group | ^65^ |
| BslA | *B. subtilis* | Bacterial | 4BHU | X-ray |  | ^135^ |
| Ywea | *B. subtilis* | Bacterial | 5MKD | X-ray |  | ^136^ |

**
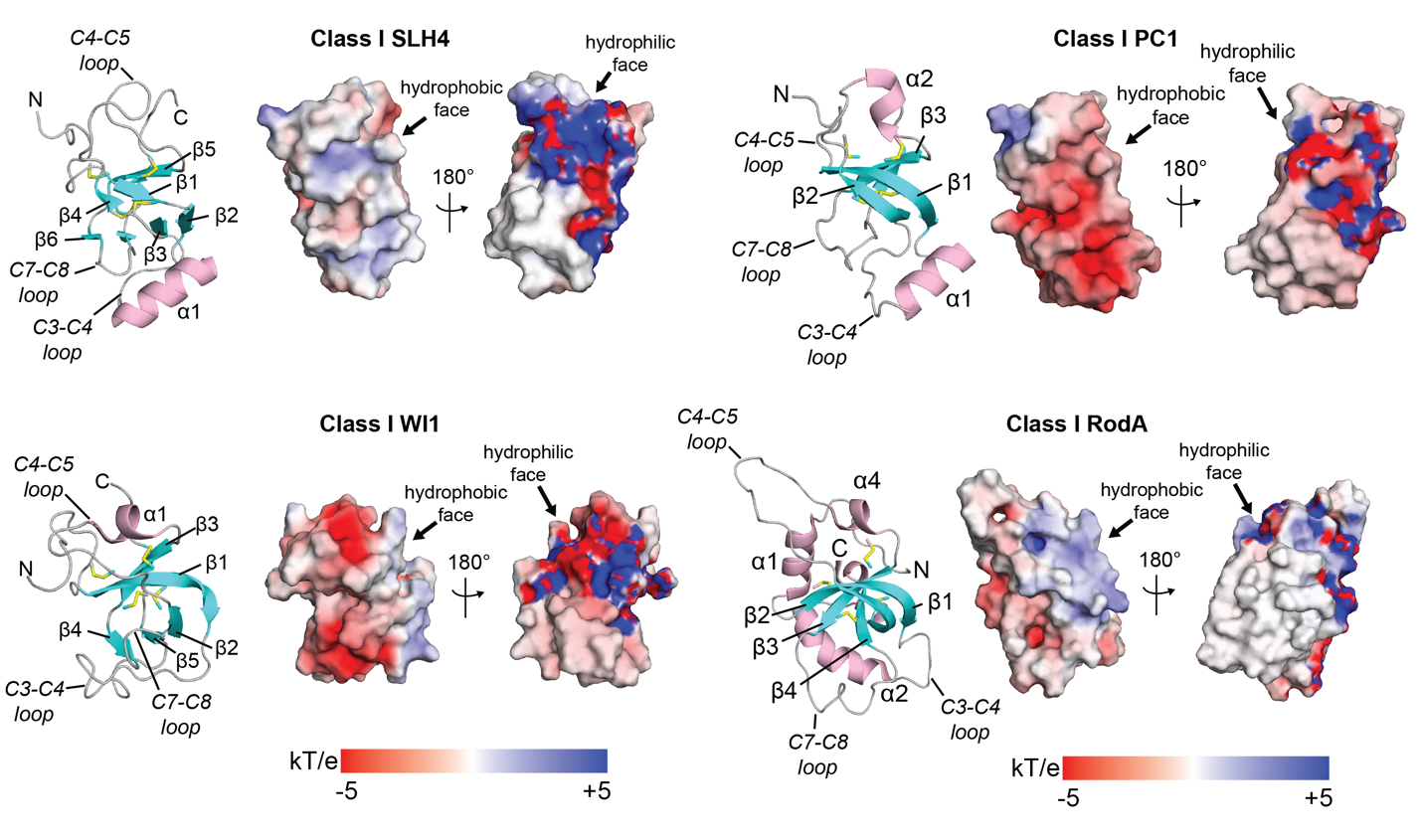
**

**Supplementary Figure 1.** **Extended general structural features of class I hydrophobins.** Additional examples of class I hydrophobin structures from the Protein Data Bank. Cartoon representations are shown with β-strands in cyan, α-helices in pink, and coils in gray. Disulfide bonds are shown as yellow sticks. Each cartoon is also accompanied by an electrostatic surface visualization calculated with the Adaptive Poisson-Boltzmann Solver (APBS plugin of PyMOL) ^110^. The contour scale for the APBS visualization is -5 kT/e (red, negative) to +5 kT/e (blue, positive). The hydrophobic (white) and hydrophilic (dark blue/dark red) faces of the amphipathic hydrophobin surface are noted. The following structures are shown: SLH4 from *Serpula lacrymans* (PDB ID 5W0Y) ^160^, WI1 from *Wallemia ichthyophaga* (PDB ID 6E9M) ^35^, PC1 from *Phanerochaete carnosa* (PDB ID 6E98) ^35^, and RodA from *Aspergillus fumigatus* (PDB ID 6GCJ) ^72^. The N-terminus (“N”), C-terminus (“C”), and secondary structure elements (α-helix, β-strands, and intercysteine loops) are noted.

**
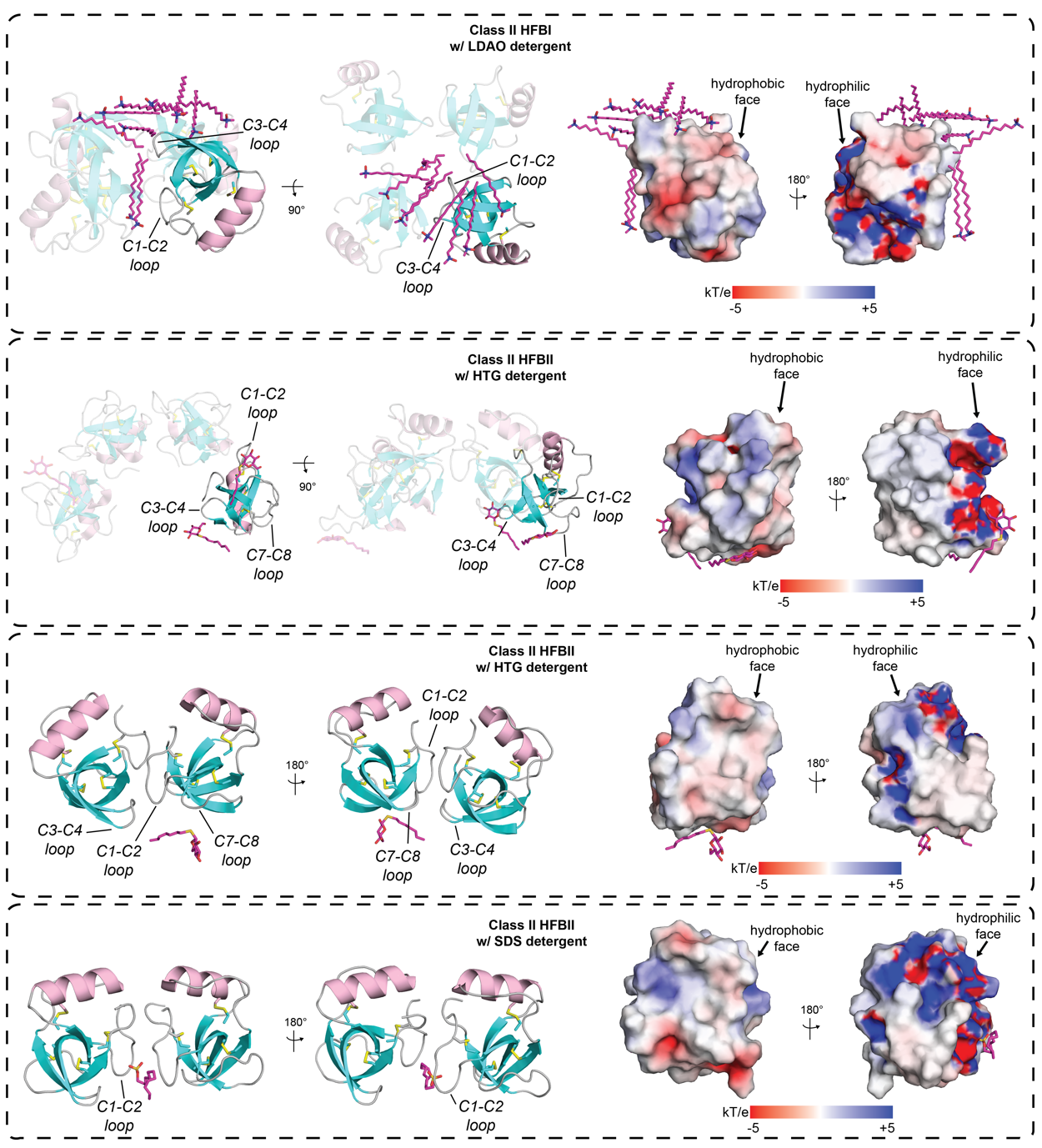
**

**Supplementary Figure 2.** **Extended general structural features of class II hydrophobins.** (A) Tetramer of class II HFBI from *T. reesei* in the presence of lauryldimethylamine-N-oxide (LDAO) detergent (PDB ID 2GVM) ^27^. LDAO detergent is shown in magenta sticks. (B) Tetramer of class II hydrophobin HFBII from *T. reesei* in the presence of heptyl-β-d-thioglycoside (HTG) detergent (PDB ID 2PL6) ^57^. HTG detergent is shown in magenta sticks. (C) Dimer of class II hydrophobin HFBII from *T. reesei* in the presence of heptyl-β-d-thioglycoside (HTG) detergent (PDB ID 2PL7) ^57^. HTG detergent is shown in magenta sticks. (D) Dimer of class II hydrophobin HFBII from *T. reesei* in presence of polystyrene nanospheres containing sodium dodecyl sulfate (SDS) detergent (PDB ID 3QQT) ^70^. SDS detergent is shown in magenta sticks. In panels A to D, each cartoon is also accompanied by an electrostatic surface visualization calculated with the Adaptive Poisson-Boltzmann Solver (APBS plugin of PyMOL) ^110^. The contour scale for the APBS visualization is -5 kT/e (red, negative) to +5 kT/e (blue, positive). The hydrophobic (white) and hydrophilic (dark blue/dark red) faces of the amphipathic hydrophobin surface are noted.

**
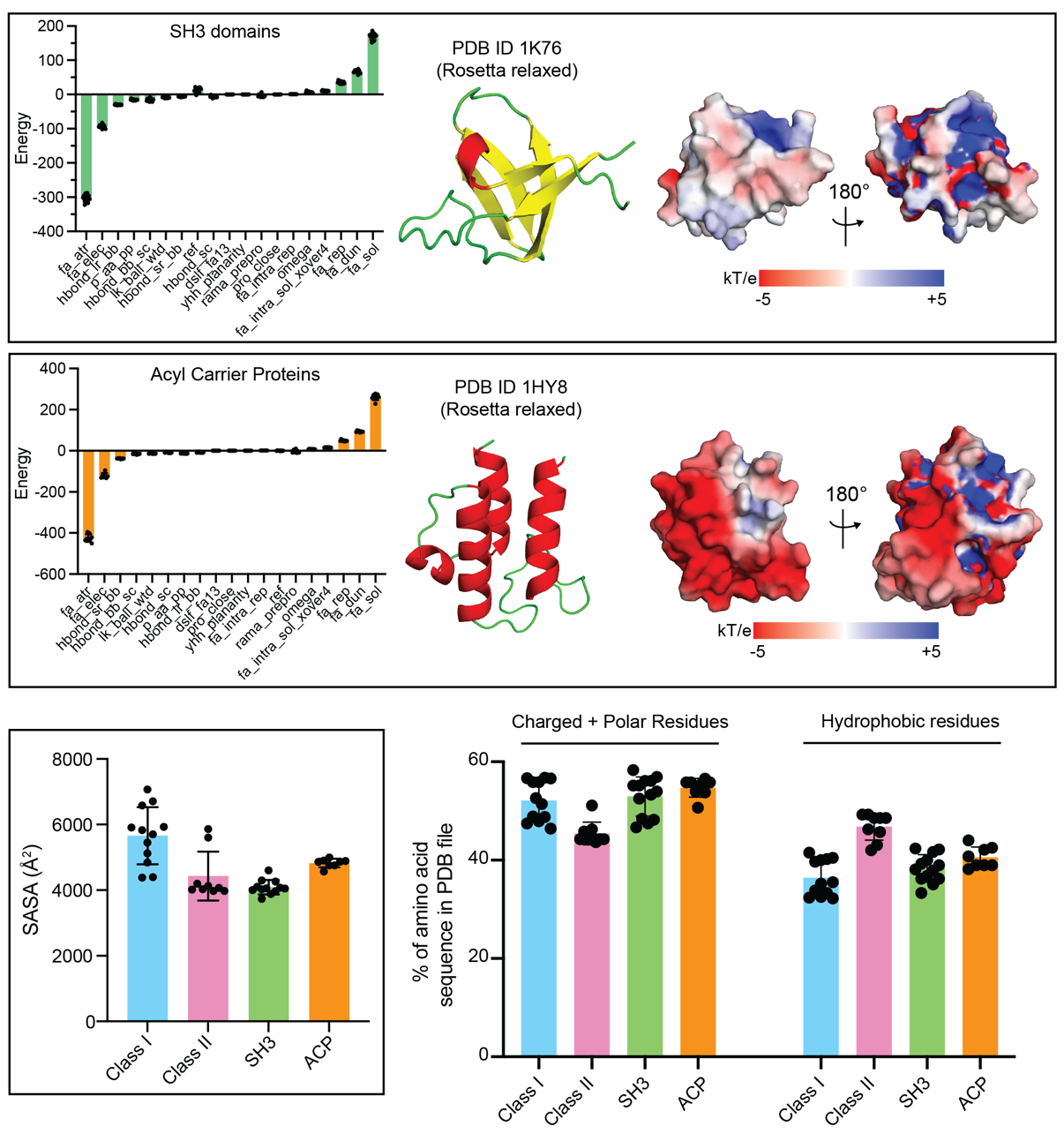
**

**Supplementary Figure 3.** ***Rosetta* evaluation of the molecular forces contributing to stability of small globular proteins.** Histogram of contributions of each *Rosetta* REF2015 score function energy term to the total energy of SH3 domains (top) and acyl carrier proteins (middle) from the Protein Data Bank. Representative structures of an SH3 domain (PDB ID 1K76) and Acyl Carrer Protein (PDB ID 1HY8) are shown. Each cartoon is also accompanied by an electrostatic surface visualization calculated with the Adaptive Poisson-Boltzmann Solver (APBS plugin of PyMOL) ^110^. The contour scale for the APBS visualization is -5 kT/e (red, negative) to +5 kT/e (blue, positive). Bottom left: Solvent-accessible surface area (SASA) determined in PyMOL for each PDB file across the different class of small globular proteins. Bottom right: The percentage of polar/charged amino acid residues and hydrophobic residues (as a percentage relative to total number of amino acids) for each PDB file across the different class of small globular proteins. Data are mean ± standard deviation where error bars are derived from separate calculations of different structures; n = 13 for SH3 domains (PDB IDs 1K76, 1NYF, 1ZLM, 2A08, 2HDA, 2VVK, 3C0C, 3I35, 3UA6, 4JZ4, 5NV1, 7A2J, 7JT9) and n = 8 for Acyl Carrier Proteins (PDB IDs 1HY8, 1L0H, 1T8K, 2EHS, 2FAC, 2K92, 2L0Q, 7E42).


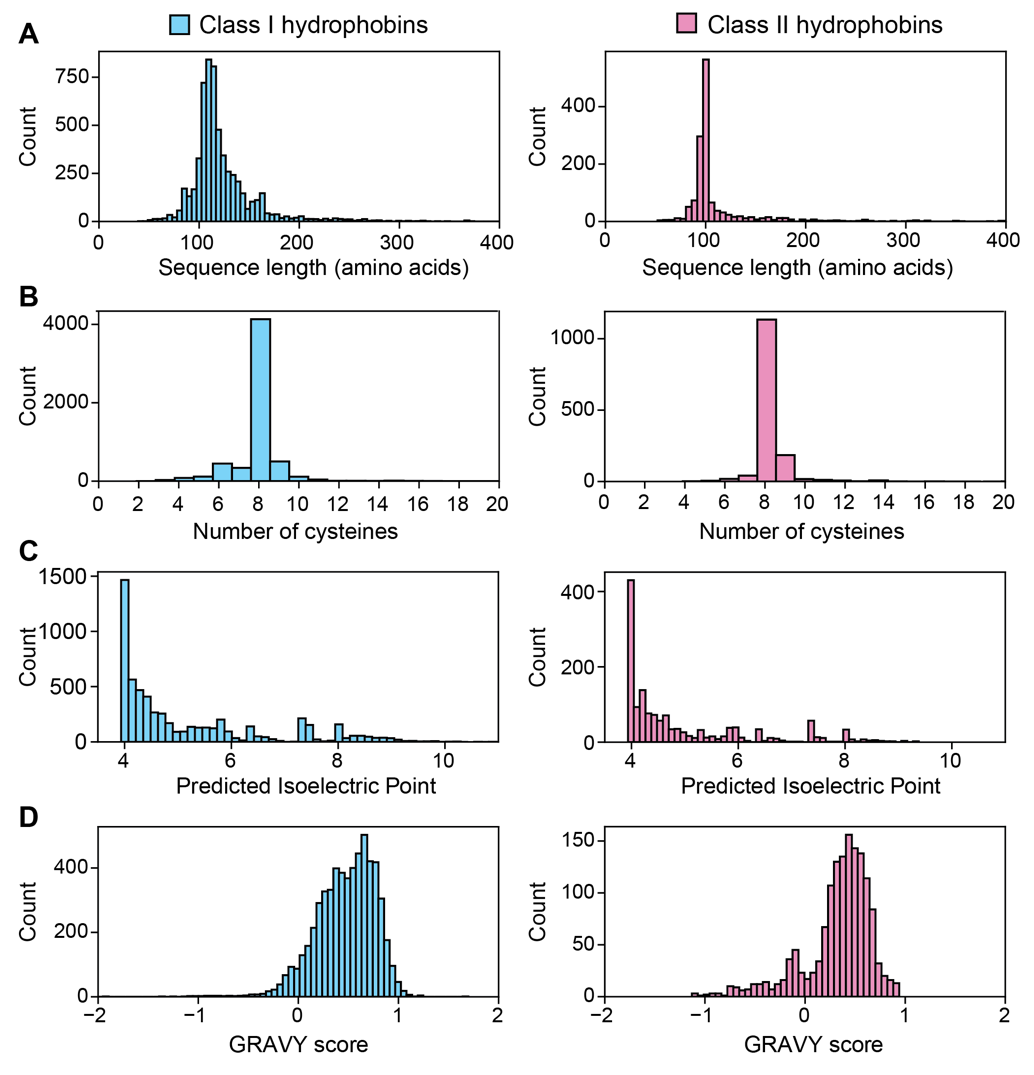


**Supplementary Figure 4.** **Global analysis of physicochemical properties of class I and class II hydrophobins.** Binned histograms of (A) total sequence length (number of amino acids), (B) number of cysteine residues, (C) predicted isoelectric point (pI), and (D) Grand Average of Hydropathy (GRAVY) score ^94^ for all 7,321 fungal class I and class II hydrophobins in the UniProt database.


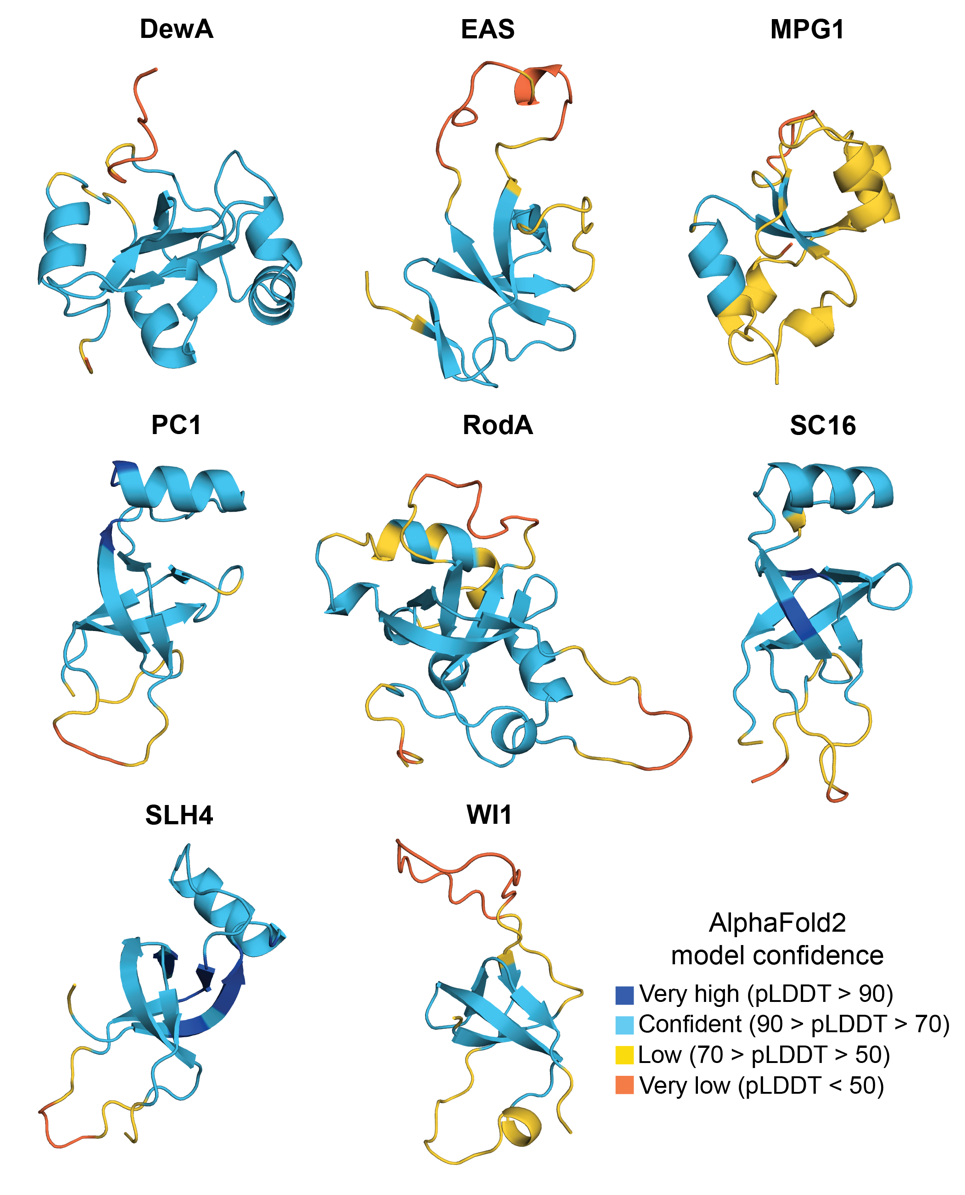


**Supplementary Figure 5.** **Per-residue AlphaFold2 model confidence for class I hydrophobins.** AlphaFold2 models of class I hydrophobins with available experimental structures (experimental structures not shown here, see Figure 1 for examples of overlays). The AlphaFold2 models are colored by per-residue predicted local-distance difference test (pLDDT) score. The flexible N-terminal tails are not shown. AlphaFold Protein Structure Database accession numbers are: AF-P52750-F1-v4 (DewA from *Aspergillus nidulans*), AF-Q04571-F1-v4 (EAS from *Neurospora crassa*), AF-P52751-F1-v4 (MPG1 from *Magnaporthe oryzae*), AF-K5VRK4-F1-v4 (PC1 from *Phanerochaete carnosa*), AF-B0Y4B1-F1-v4 (RodA from *Aspergillus fumigatus*), AF-D8QCG9-F1-v4 (SC16 from *Schizophyllum commune*), AF-F8NJA2-F1-v4 (SLH4 from *Serpula lacrymans*), and AF-R9A9N7-F1-v4 (WI1 from *Wallemia ichthyophaga*).


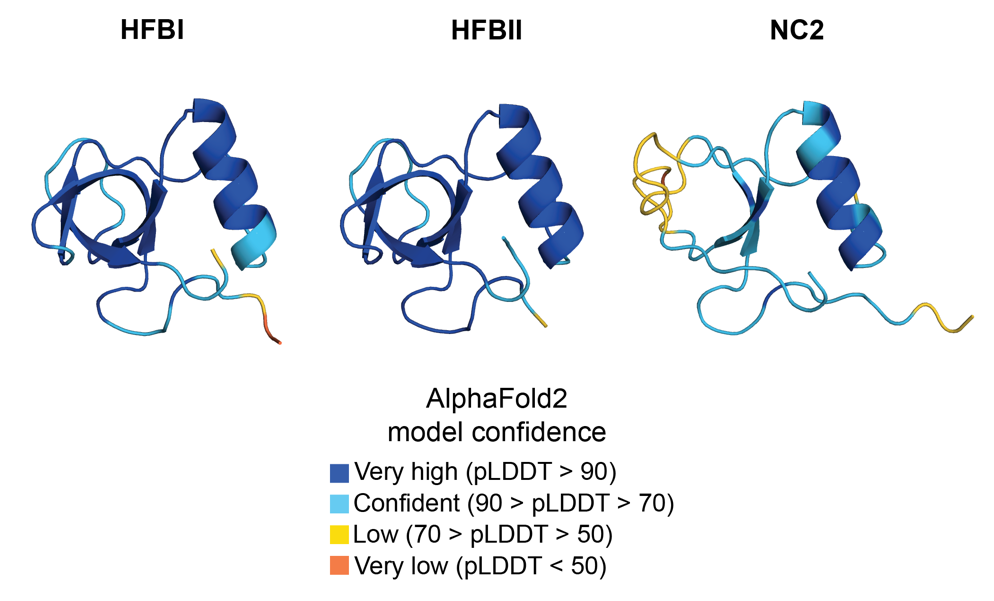


**Supplementary Figure 6.** **Per-residue AlphaFold2 model confidence for class II hydrophobins.** AlphaFold2 models of class II hydrophobins with available experimental structures (experimental structures not shown here, see Figure 1 for examples of overlays). The AlphaFold2 models are colored by per-residue predicted local-distance difference test (pLDDT) score. The flexible N-terminal tails are not shown. AlphaFold Protein Structure Database accession numbers are: AF-P52754-F1-v4 (HFBI from *Trichoderma reesei*), AF-P79073-F1-v4 (HFBII from *Trichoderma reesei*), and AF-Q7S3P5-F1-v4 (NC2 from *Neurospora crassa*).


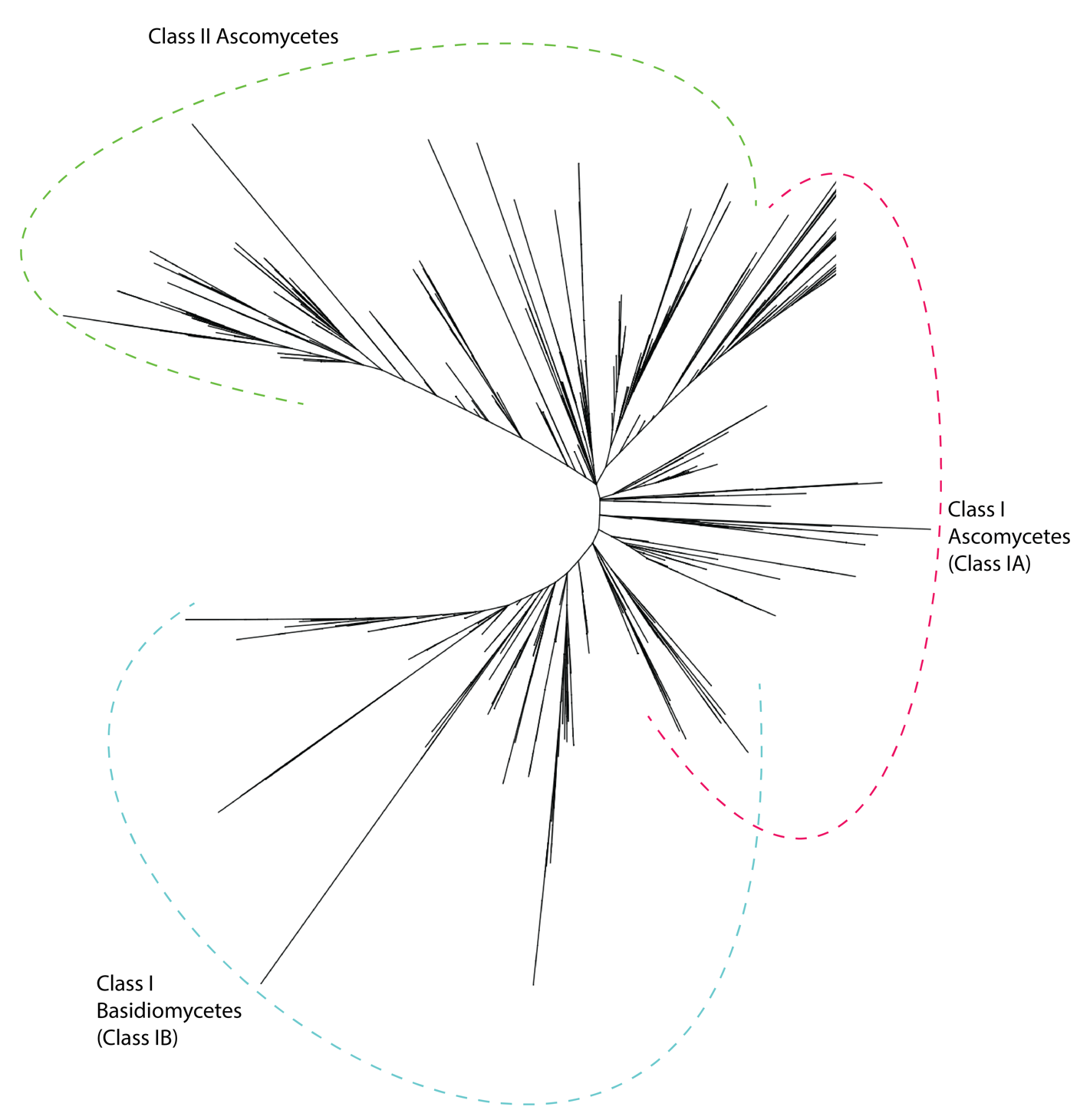


**Supplementary Figure 7.** **Sequence-based dendrogram of class I and class II hydrophobins.**

An unrooted sequence-based dendrogram generated by the combination of Clustal Omega, TrimAI, and iTOL (see Methods for details) resulting with 6,754 leaves (i.e., sequences) ^103^. Each section of the tree is labeled with the corresponding hydrophobin class.


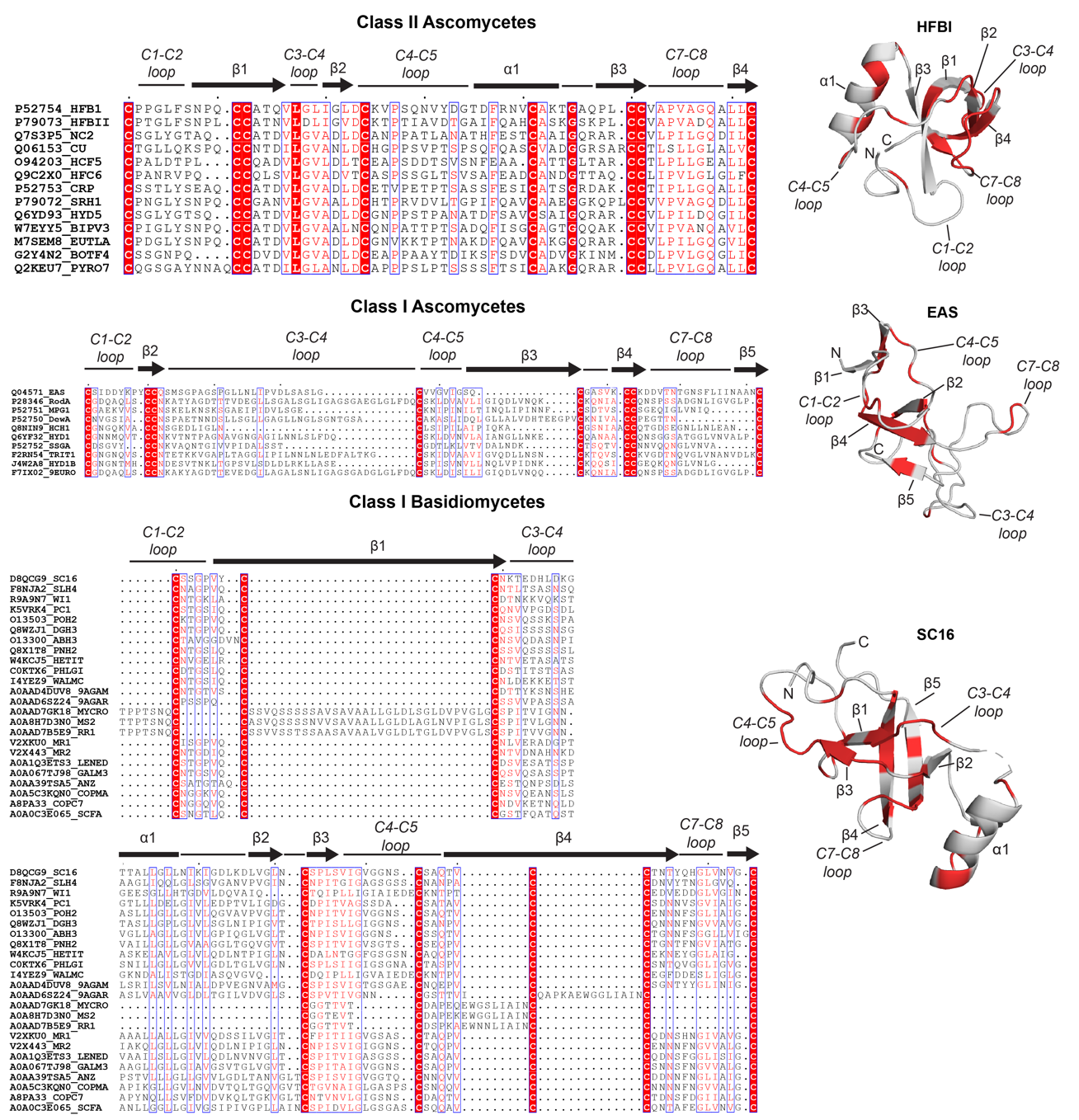


**Supplementary Figure 8.** **Relationship between sequence conservation and structural elements for canonical class I and class II hydrophobins.**

Left: sequence alignment between different hydrophobin classes performed with Clustal Omega 1.2.4 and processed with ESPript 3. Above each sequence is a cartoon of the secondary structure elements of a representative class member (HFBI, EAS, SC16). Right: Experimental structures of representative class members with conserved or partially conserved residues colored red. Residues lacking conservation are shown in gray. The following structures are shown: Class IA EAS from *Neurospora crassa* (PDB ID 2FMC) ^45^, Class IB SC16 from *Schizophyllum commune* (PDB ID 2NBH) ^34^, and Class I HFBI from *Trichoderma reesei* (PDB ID 2FZ6) ^27^.


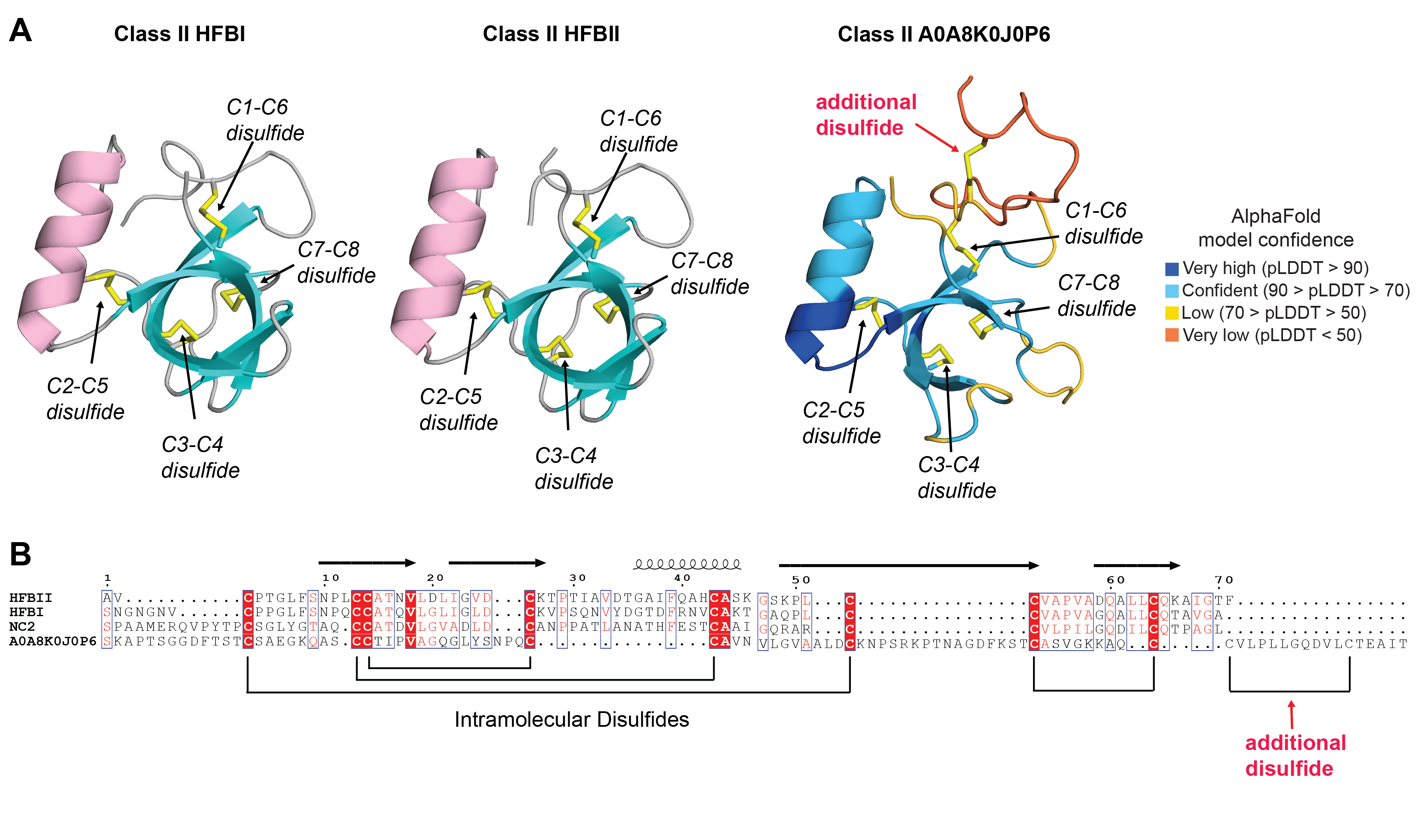


**Supplementary Figure 9.** **Example of a putative class II hydrophobin with five disulfide bonds.** From left to right: Reference class II hydrophobins with four disulfide bonds: HFBI from *Trichoderma reesei* (PDB ID 2FZ6 - Class II) ^27^ and HFBII from *Trichoderma reesei* (PDB ID 1R2M - Class II) ^27^. AlphaFold model of an example putative class II hydrophobin with five disulfide bonds: UniProt ID A0A8K0J0P6 (from *Claviceps africana*). The canonical disulfide bonds are label with their respective Cys residue numbers (i.e., C1-C6 – a disulfide bond between the first and sixth Cys residue in the sequence). The “new” putative disulfide bond is denoted with red arrow. The AlphaFold model is colored by per-residue predicted local-distance difference test (pLDDT) score. (B) Sequence alignment of HFBI, HFBII, and A0A8K0J0P6 performed with Clustal Omega 1.2.4 and processed with ESPript 3. The disulfide bond connectives, including the new putative disulfide bond (i.e., C9-C10), are noted.


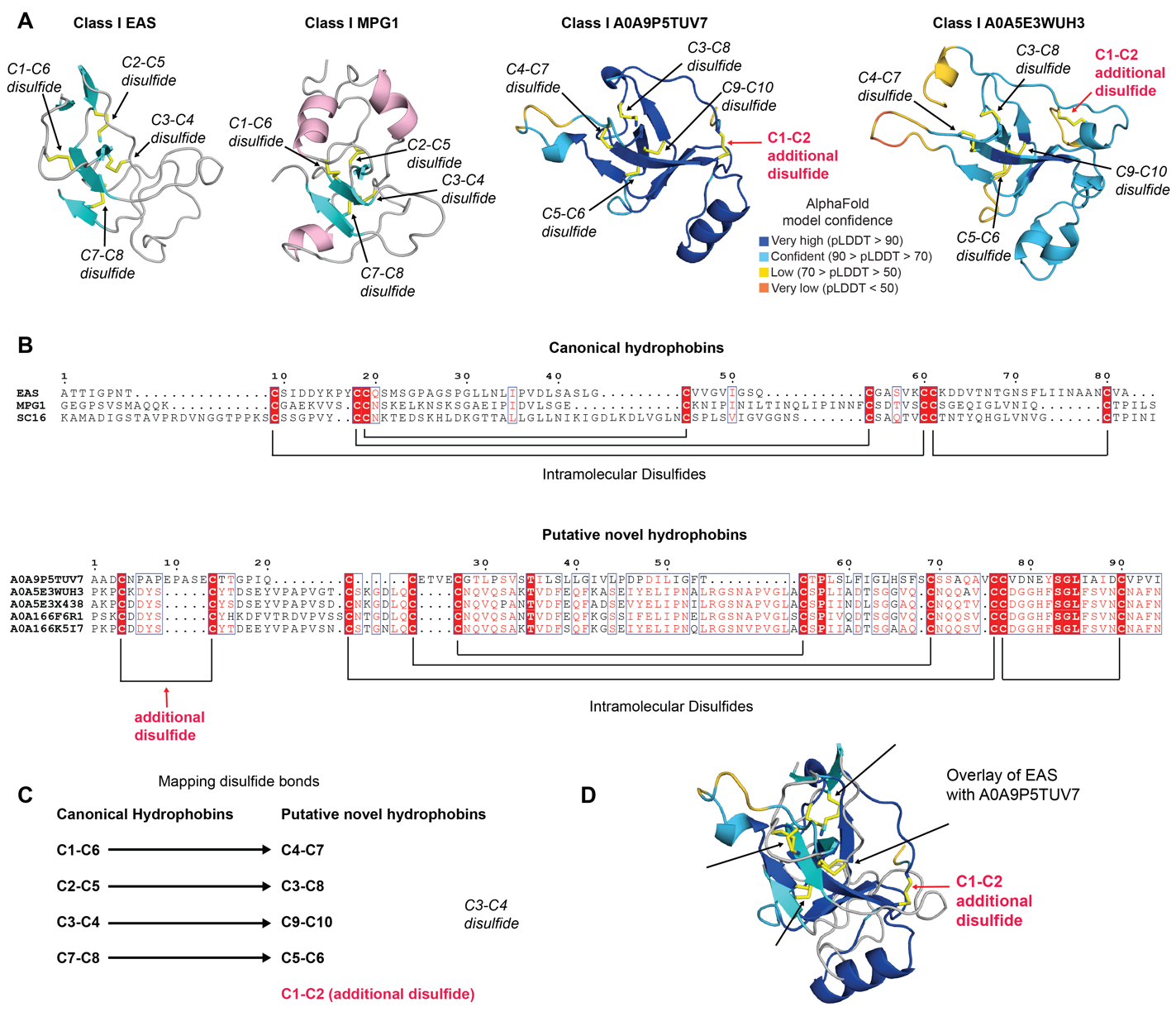


**Supplementary Figure 10.** **Examples of putative class I hydrophobins with five disulfide bonds.** (A) From left to right: Reference class I hydrophobins with four disulfide bonds: MPG1 from *Magnaporthe oryzae* (PDB ID 2N4O - Class I) ^158^ and EAS from *Neurospora crassa* (PDB ID 2FMC - Class I) ^45^. AlphaFold models of example putative class I hydrophobins with five disulfide bonds: UniProt ID A0A9P5TUV7 (from *Rhodocollybia butyracea*) and UniProt ID A0A5E3WUH3 (from *Peniophora sp.* CBMAI 1063). On the left: the canonical disulfide bonds are label with their respective Cys residue numbers (i.e., C1-C6 – a disulfide bond between the first and sixth Cys residue in the sequence). On the right: the unique disulfide bonding nomenclature for the putative novel hydrophobins are provided; the “new” putative disulfide bond is denoted with red arrow. The AlphaFold models are colored by per-residue predicted local-distance difference test (pLDDT) score. (B) Sequence alignment of EAS, MPG1, SC16 and several representative putative hydrophobins with five disulfide bonds performed with Clustal Omega 1.2.4 and processed with ESPript 3. The connectivity for the putative disulfide bonds is noted. (C) Mapping of canonical hydrophobin disulfide bonds with the putative novel hydrophobins. (D) Overlay of EAS with A0A9P5TUV7 structures showing spatial overlap of disulfide bonds.


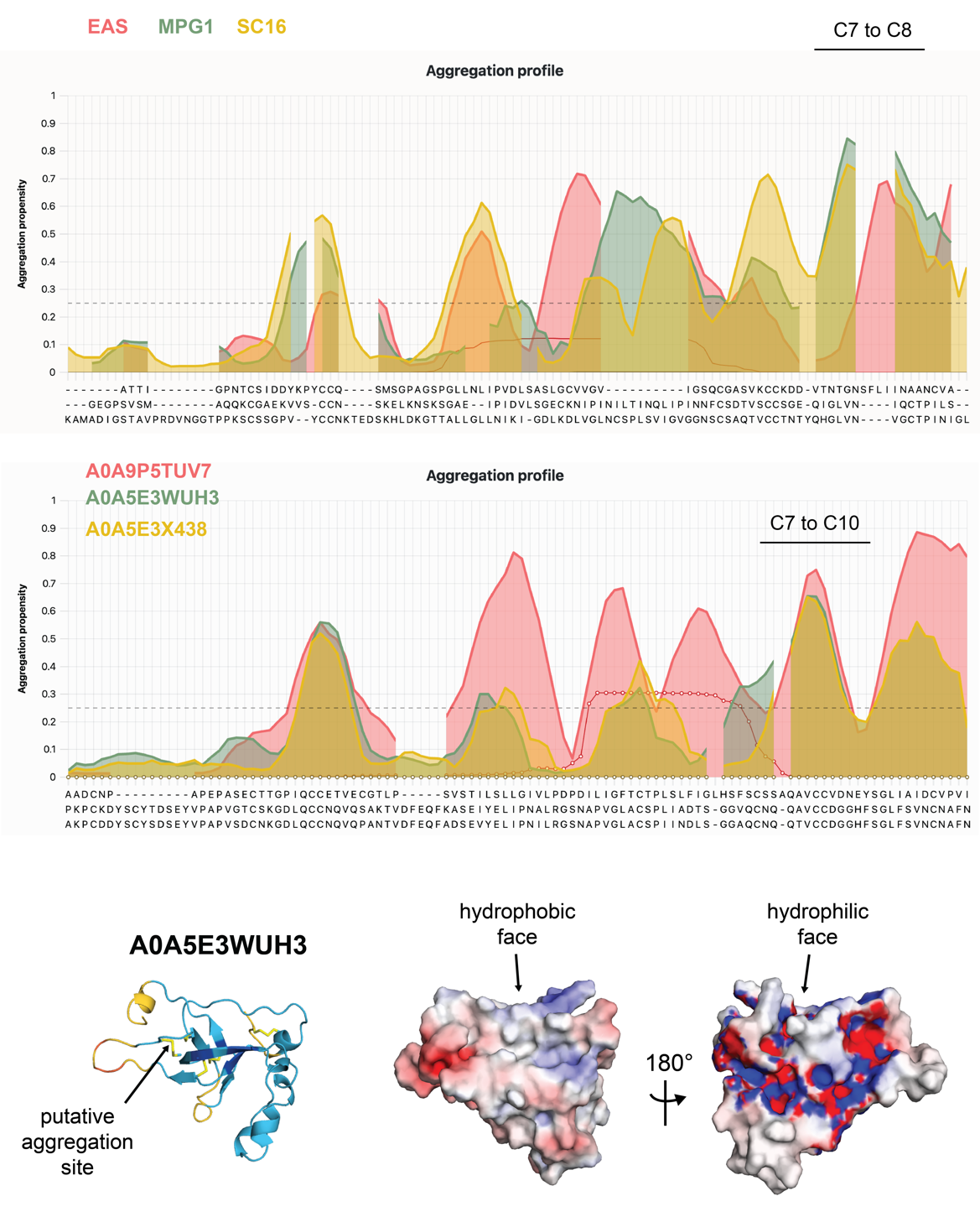


**Supplementary Figure 11.** ***In silico* prediction of aggregation sites for putative class I hydrophobins with five disulfide bonds.** Prediction of residues involved in aggregation for canonical hydrophobics (top) and non-canonical hydrophobins with five disulfide bonds (bottom) by the AggreProt webserver v1 ^108^. Regions with the largest aggregation propensity that is consistent across the three pairs of proteins are highlighted. The bottom shows an example AlphaFold model of A0A5EWUH3 accompanied by an electrostatic surface visualization calculated with the Adaptive Poisson-Boltzmann Solver (APBS plugin of PyMOL) ^110^. The contour scale for the APBS visualization is -5 kT/e (red, negative) to +5 kT/e (blue, positive). The hydrophobic (white) and hydrophilic (dark blue/dark red) faces of the amphipathic hydrophobin surface are noted. The location of the putative aggregation surface is noted.


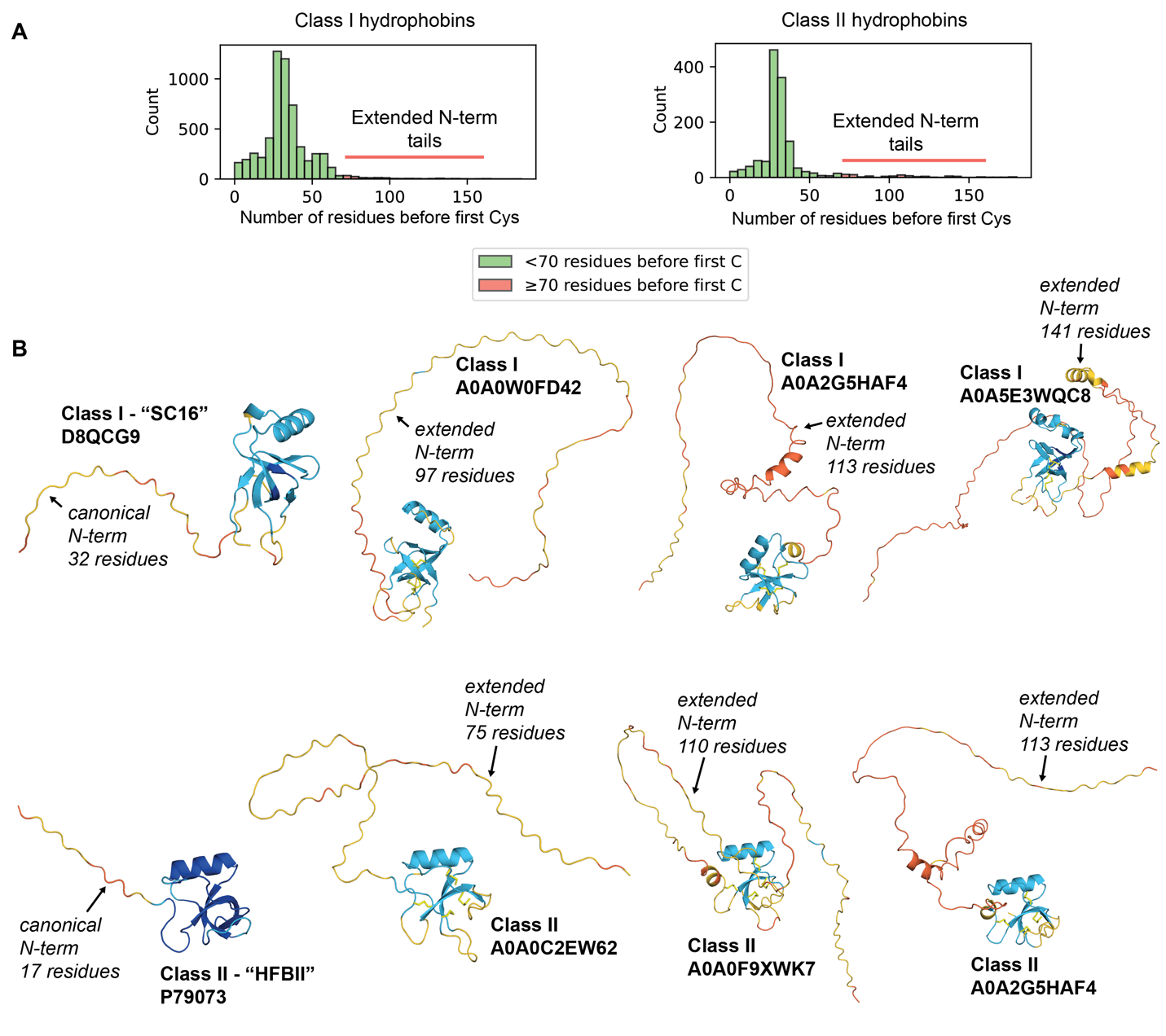
**Supplementary Figure 12.** **Examples of putative class I and class II hydrophobins with extended disordered N-terminal tails.** (A) Binned histograms of the number of residues occurring before the first Cys in the sequence vs count of sequences for class I and class II hydrophobins. The orange bars highlight sequences with > 70 residues before the first Cys. These sequences are considered hydrophobins with extended N-terminal domains. (B) AlphaFold models of canonical class I (SC16) and class II (HFBII) hydrophobins with number of residues in the canonical N-terminal tails noted. AlphaFold models are also shown for non-canonical class I and class II hydrophobins with extended disordered N-terminal tails; the associated UniProt ID accession numbers are noted. For class I: A0A0W0FD42 from *Moniliophthora roreri*, A0A2G5HAF4 from *Cercospora beticolam*, and A0A5E3WQC8 from *Peniophora sp*. For class II: A0A0C2EW62 from *Sporothrix brasiliensis*, A0A0F9XWK7 from *Trichoderma harzianum*, and A0A2G5HAF4 from *Cercospora beticola*. AlphaFold models are colored by per residue pLDDT score.

**
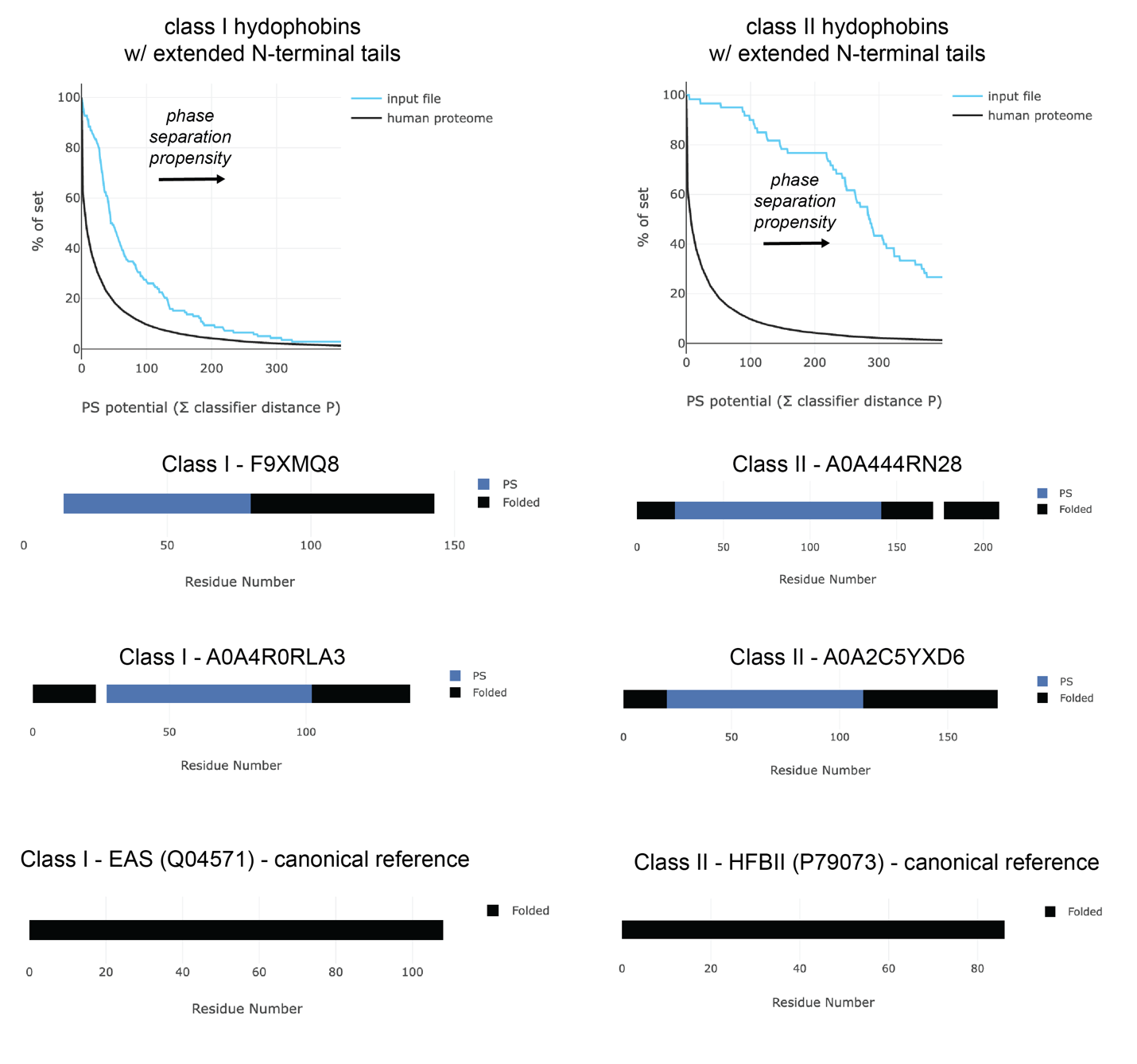
**

**Supplementary Figure 13.** **Examples of phase separation potential of putative class I and class II hydrophobins with extended disordered N-terminal tails.** Top: Distribution of phase separation (PS) potentials calculated by ParSe v2 from input sequences (<https://stevewhitten.github.io/Parse_v2_FASTA/>). ParSe v2 calculates the classifier distance sum of windows labeled P, which are used as a numerical score to estimate the PS potential. The y-axis is the percent of proteins in a set with a PS potential equal to or greater than the value indicated by the x-axis; the human proteome is included as a reference comparison dataset. Bottom: Cartoon schematic of residues predicted by ParSe v2 to undergo phase separation (PS) or be a folded domain (i.e., the hydrophobin fold or signal peptide) obtained from <https://stevewhitten.github.io/Parse_v2_web/>.


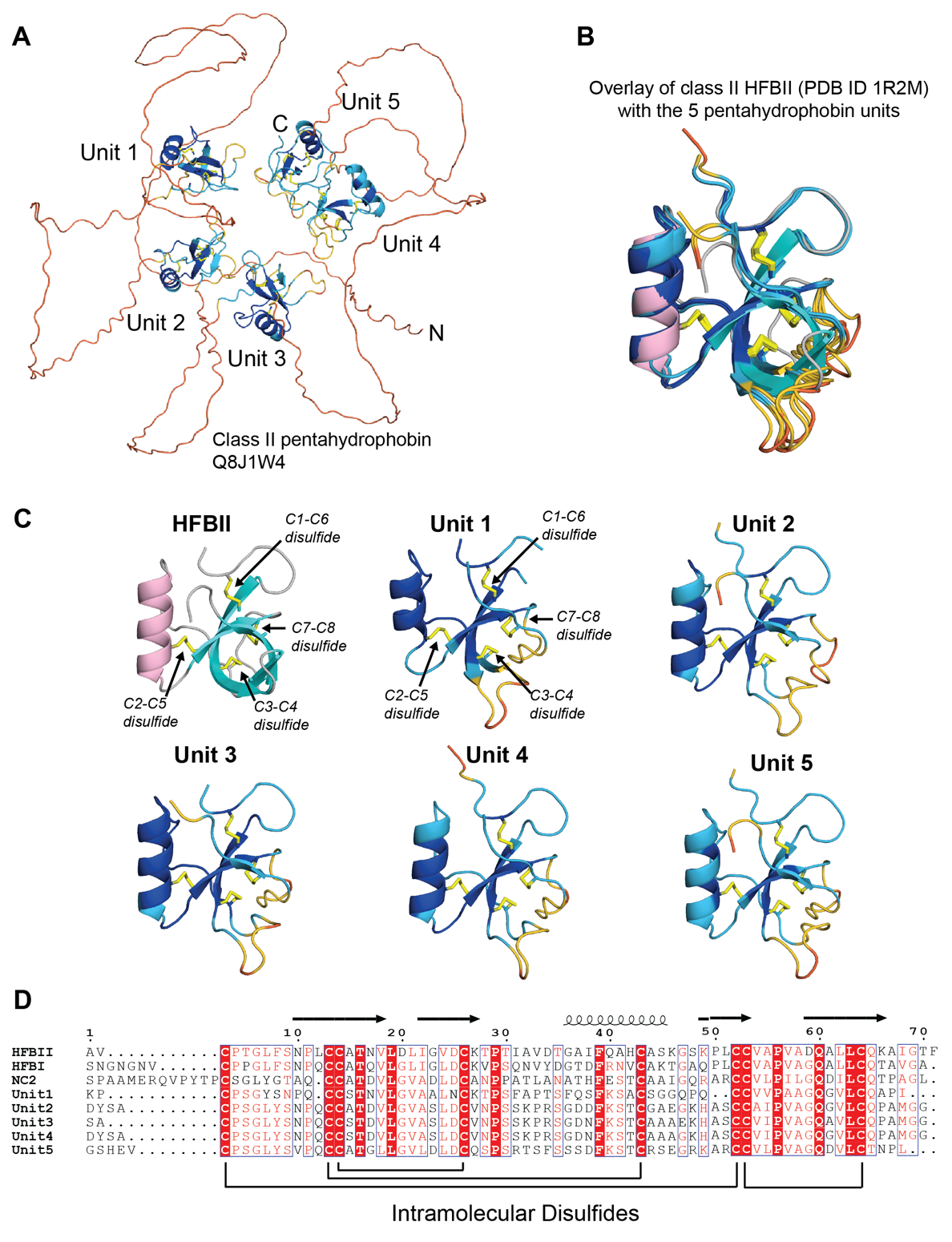


**Supplementary Figure 14.** **The hydrophobin units from class II pentahydrophobins are highly conserved in both sequence and structure with monomeric class I hydrophobins.** (A) AlphaFold model of UniProt #Q8J1W4-F1, a class II pentahydrophobin CPPH1 from *Claviceps purpurea*, colored by per-residue predicted local-distance difference test (pLDDT) score. (B) Overlay of the individual units of the pentahydrophobin with HFBII from *Trichoderma reesei* (PDB ID 1R2M - Class II) ^27^. (C) Comparison of HFBII with individual units of the pentahydrophobin. Sequence alignment of HFBI, HFBII, NC2, and the individual units of the pentahydrophobin performed with Clustal Omega 1.2.4 and processed with ESPript 3. The disulfide bond connectives are noted.


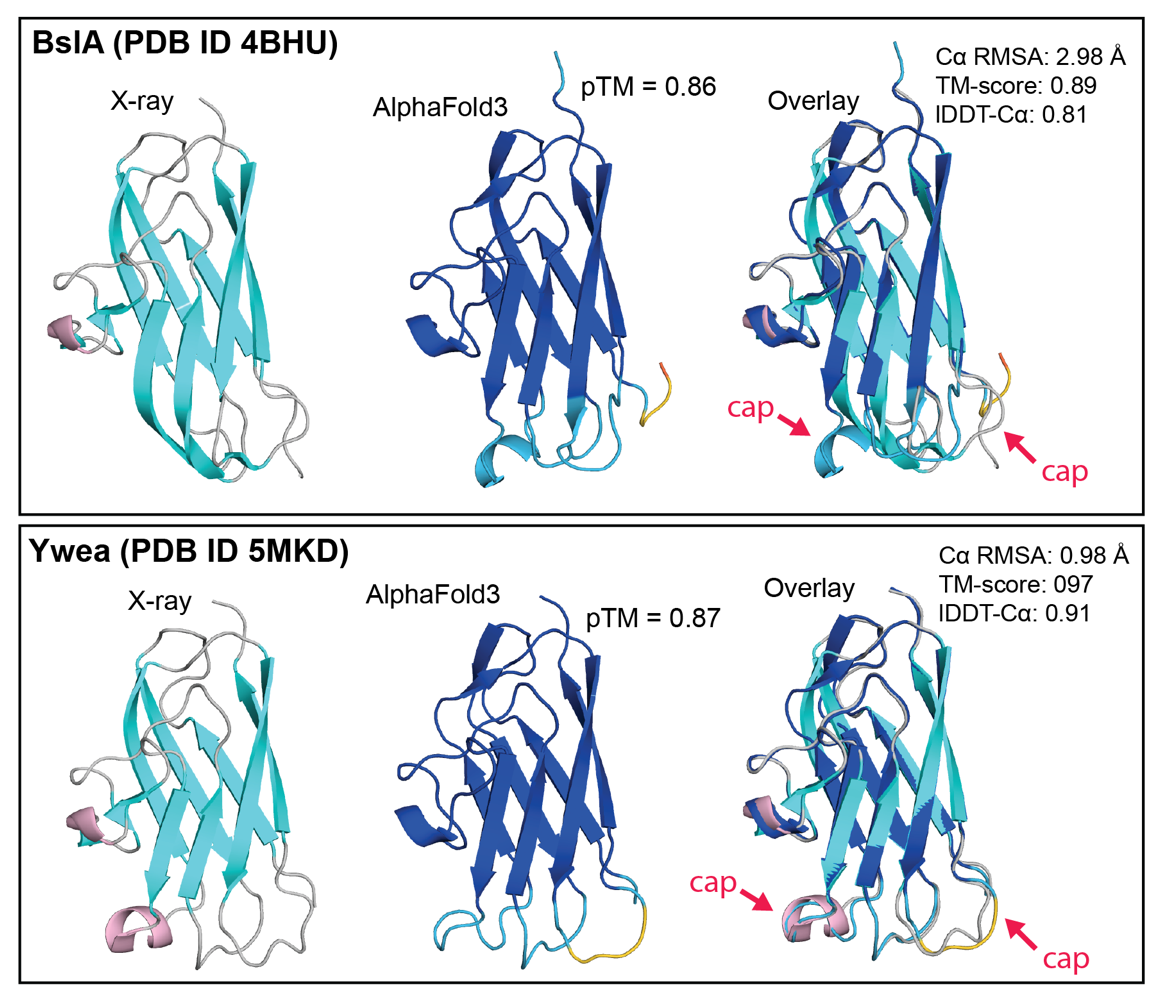


**Supplementary Figure 15.** **AlphaFold predicts the structures of bacterial hydrophobins with moderate to high confidence except for in the “cap” region.** Left: X-ray structures of bacterial hydrophobins: PDB ID 4BHU (bacterial hydrophobin BslA from *Bacillus subtilis*) ^135^ and PDB ID 5MKD (bacterial hydrophobin Ywea from *Bacillus subtilis*) ^136^. Middle: AlphaFold3 models of bacterial hydrophobins colored by per residue pLDDT score with the pMT scores. Right: Overlay of X-ray structures and AlphaFold3 models with corresponding structural comparison metrics.


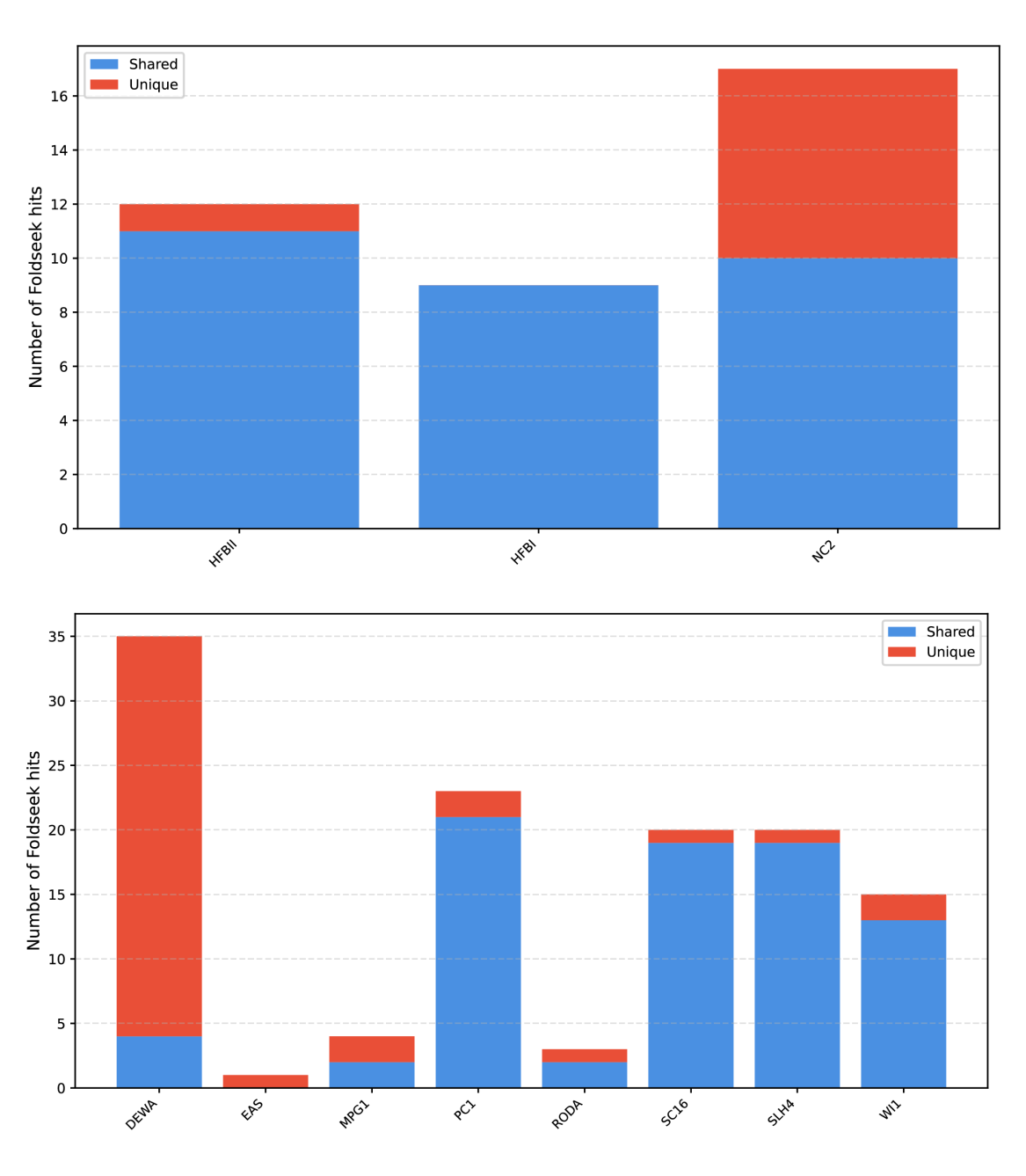


**Supplementary Figure 16.** **Summary of** **Foldseek hits using different “bait” hydrophobin structures.** Hits are considered any polypeptide containing a hydrophoin-like domain that is not a canonical hydrophobin. The number of shared and unique Foldseek hits from different hydrophobin bait proteins are shown as a function of each bait protein.


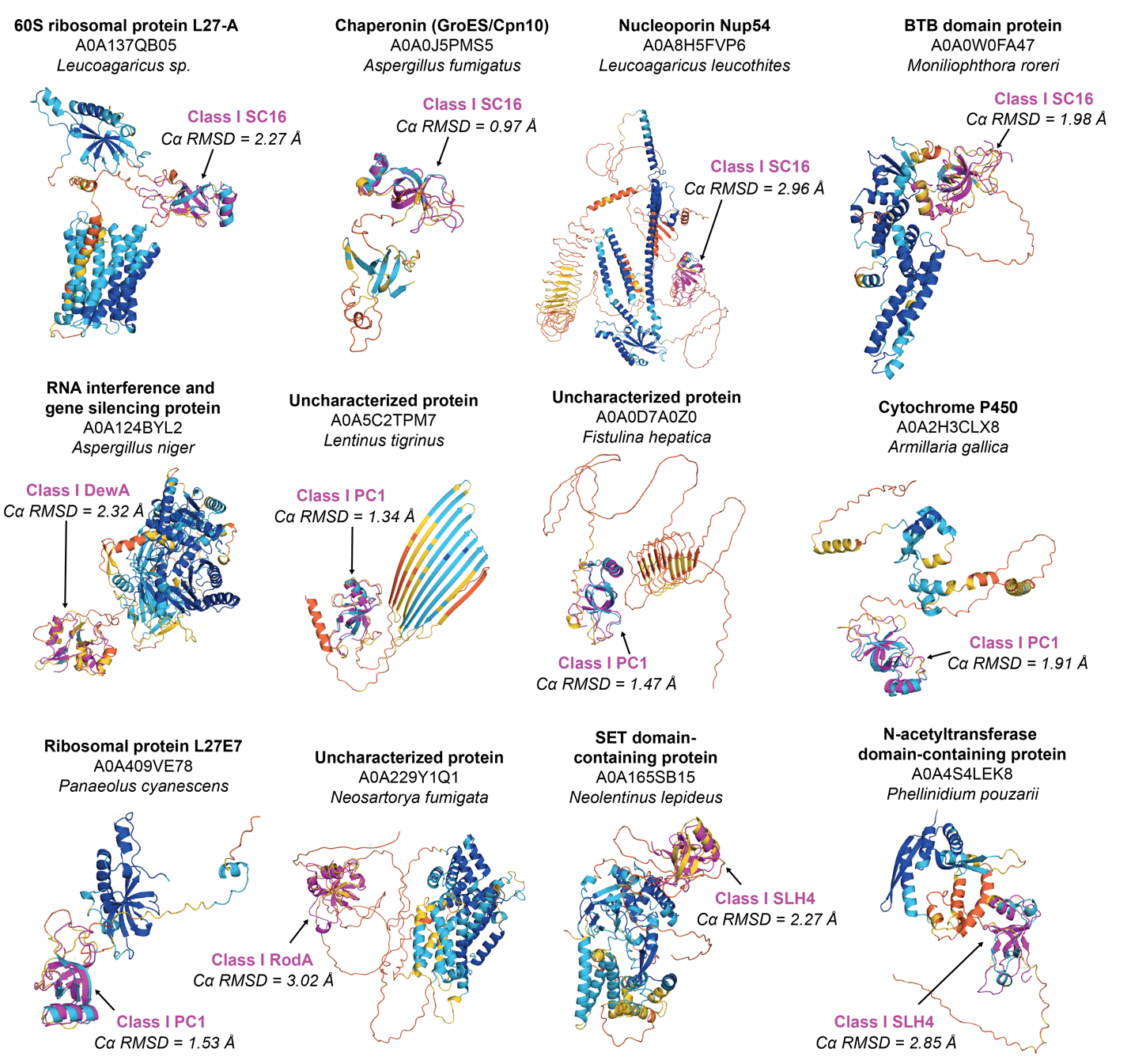


**Supplementary Figure 17.** **Extended examples of** **Foldseek hits for class I hydrophobin bait proteins.** Comparison of experimental hydrophobin structures (magenta) overlaid with AlphaFold models of proteins containing hydrophobin-like domains (colored by pLDDT score). Cα RMSD values (Ångströms, Å) each hydrophobin relative to the hydrophobin-like domain are noted. AlphaFold models are colored by per residue pLDDT score.


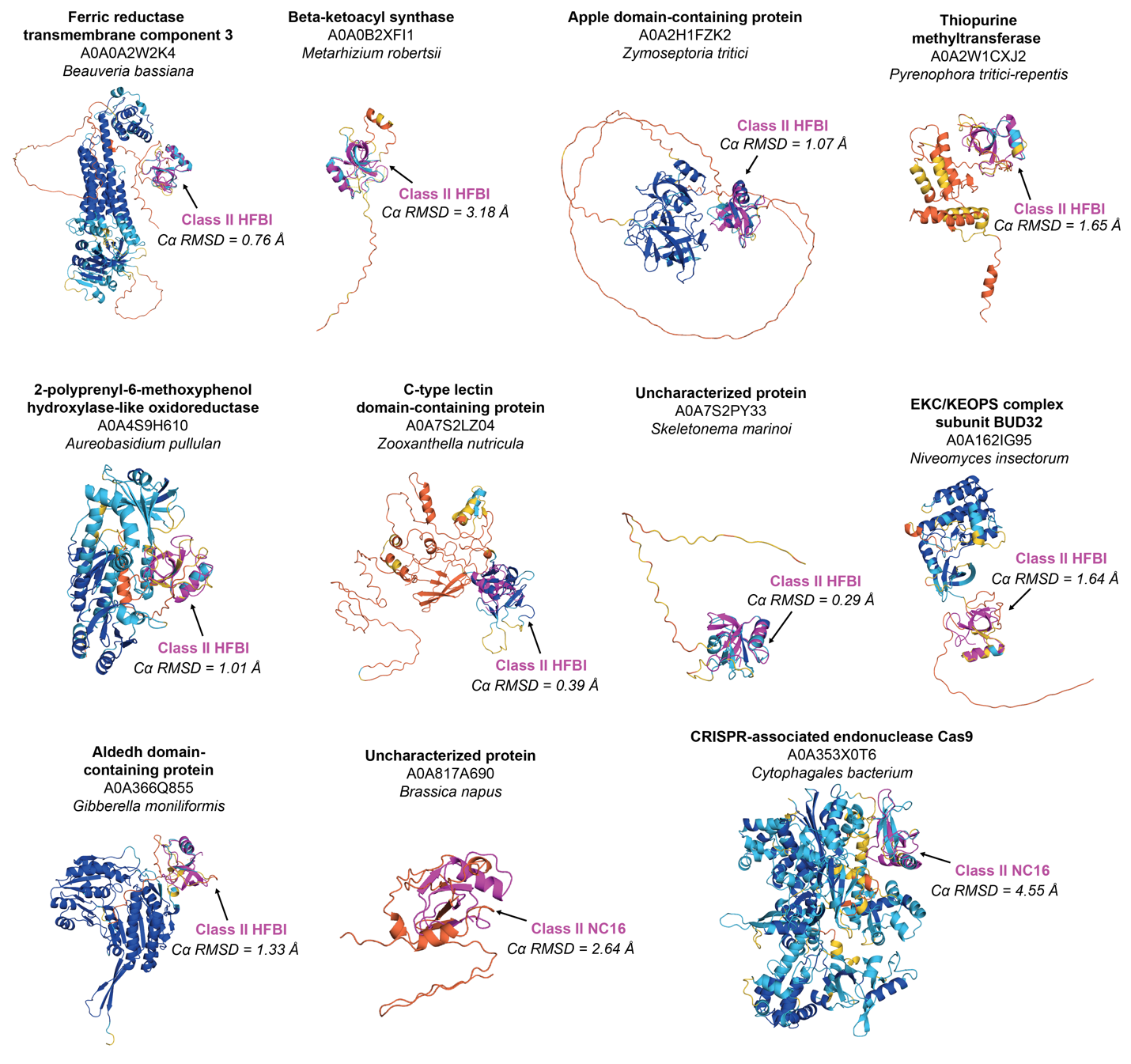


**Supplementary Figure 18.** **Extended examples of** **Foldseek hits for class II hydrophobin bait proteins.** Comparison of experimental hydrophobin structures (magenta) overlaid with AlphaFold models of proteins containing hydrophobin-like domains (colored by pLDDT score). Cα RMSD values (Ångströms, Å) each hydrophobin relative to the hydrophobin-like domain are noted. AlphaFold models are colored by per residue pLDDT score.


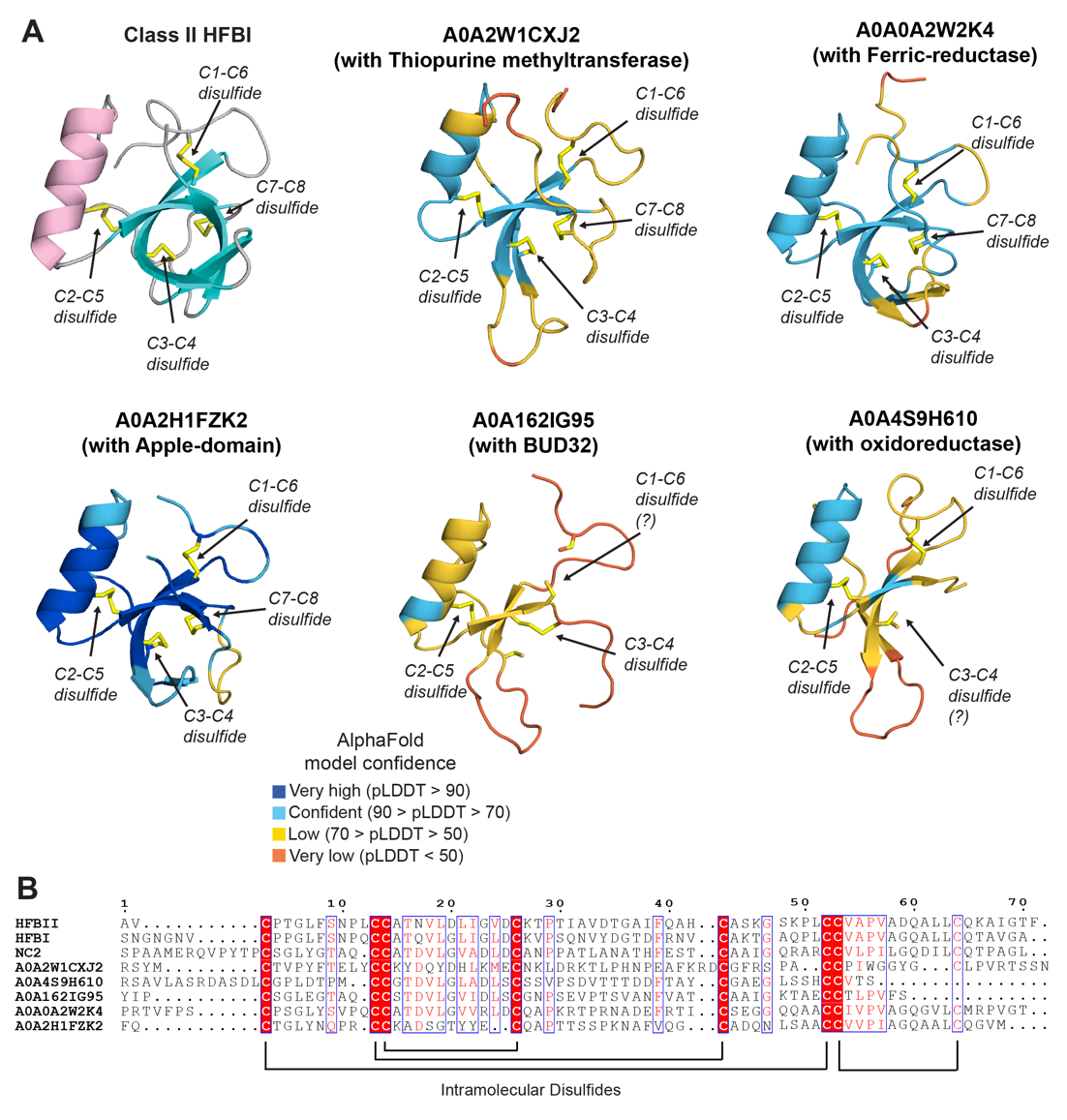


**Supplementary Figure 19.** **The hydrophobin-like domains from polypeptide chains within other proteins are conserved in both sequence and structure with monomeric class II hydrophobins.** (A) Comparison of the experimental reference structure for a canonical class II hydrophobin, HFBI (PDB ID 2FZ6), with AlphaFold models of the hydrophobin-like domains from polypeptide chains containing other domains. The UniProt ID and the type of other protein domain are noted. AlphaFold models are colored by per residue pLDDT score. (B) Sequence alignment of HFBI, HFBII, NC2, and the hydrophobin-like domains from polypeptide chains within other proteins performed with Clustal Omega 1.2.4 and processed with ESPript 3. The disulfide bond connectives are noted. Note that some of the hydrophobin-like domains do not have contain all canonical disulfide bonds, but many do.


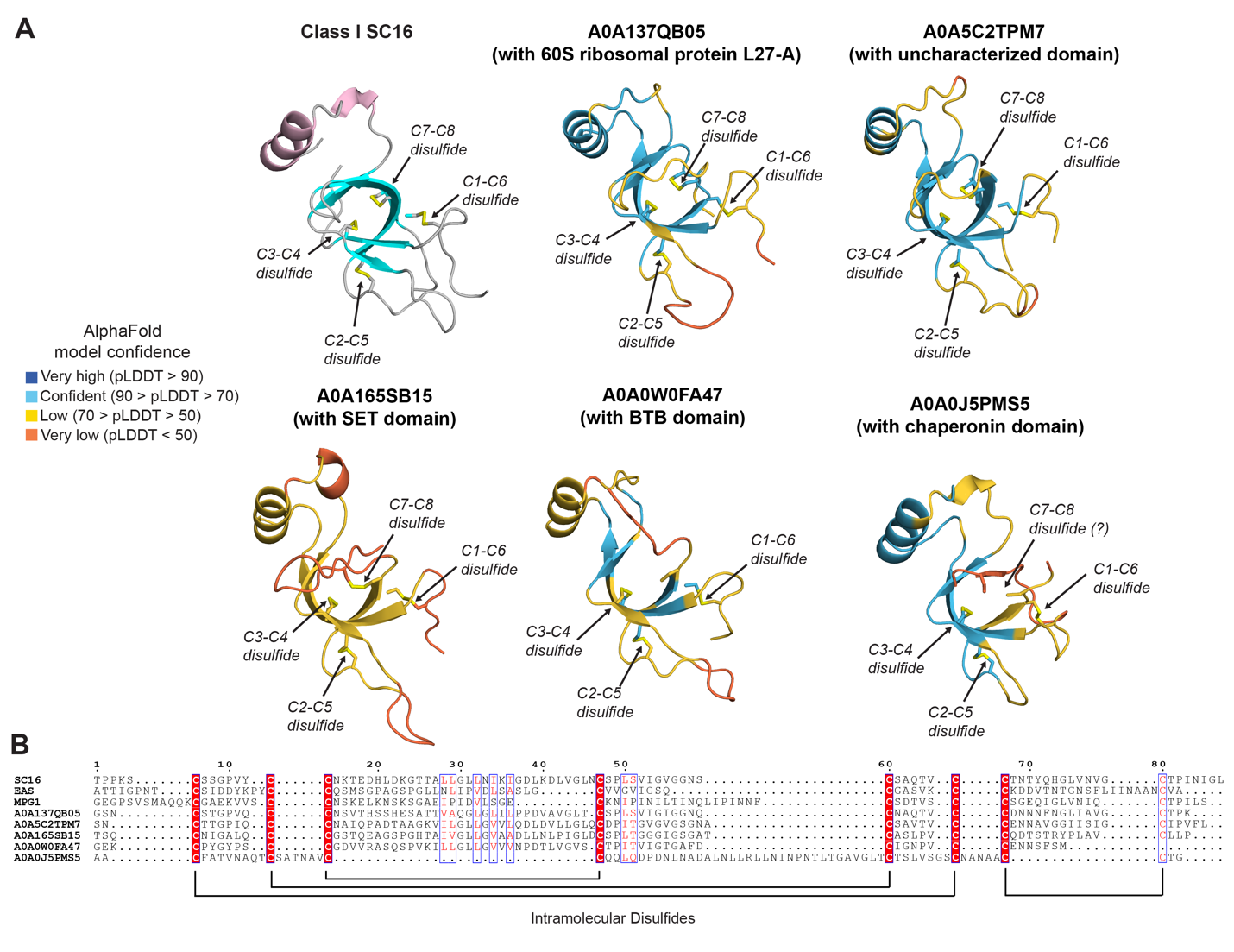


**Supplementary Figure 20.** **The hydrophobin-like domains from polypeptide chains within other proteins are conserved in both sequence and structure with monomeric class I hydrophobins.** (A) Comparison of the experimental reference structure for a canonical class I hydrophobin, SC16 (PDB ID 2NBH), with AlphaFold models of the hydrophobin-like domains from polypeptide chains containing other domains. The UniProt ID and the type of other protein domain are noted. AlphaFold models are colored by per residue pLDDT score. (B) Sequence alignment of SC16, EAS, MPG1, and the hydrophobin-like domains from polypeptide chains within other proteins performed with Clustal Omega 1.2.4 and processed with ESPript 3. The disulfide bond connectives are noted. Note that some of the hydrophobin-like domains do not have contain all canonical disulfide bonds, but many do. Many of these hydrophobin-like domains also contain hydrophobin residues in the C7-C8 loop that could play roles in self-assembly.


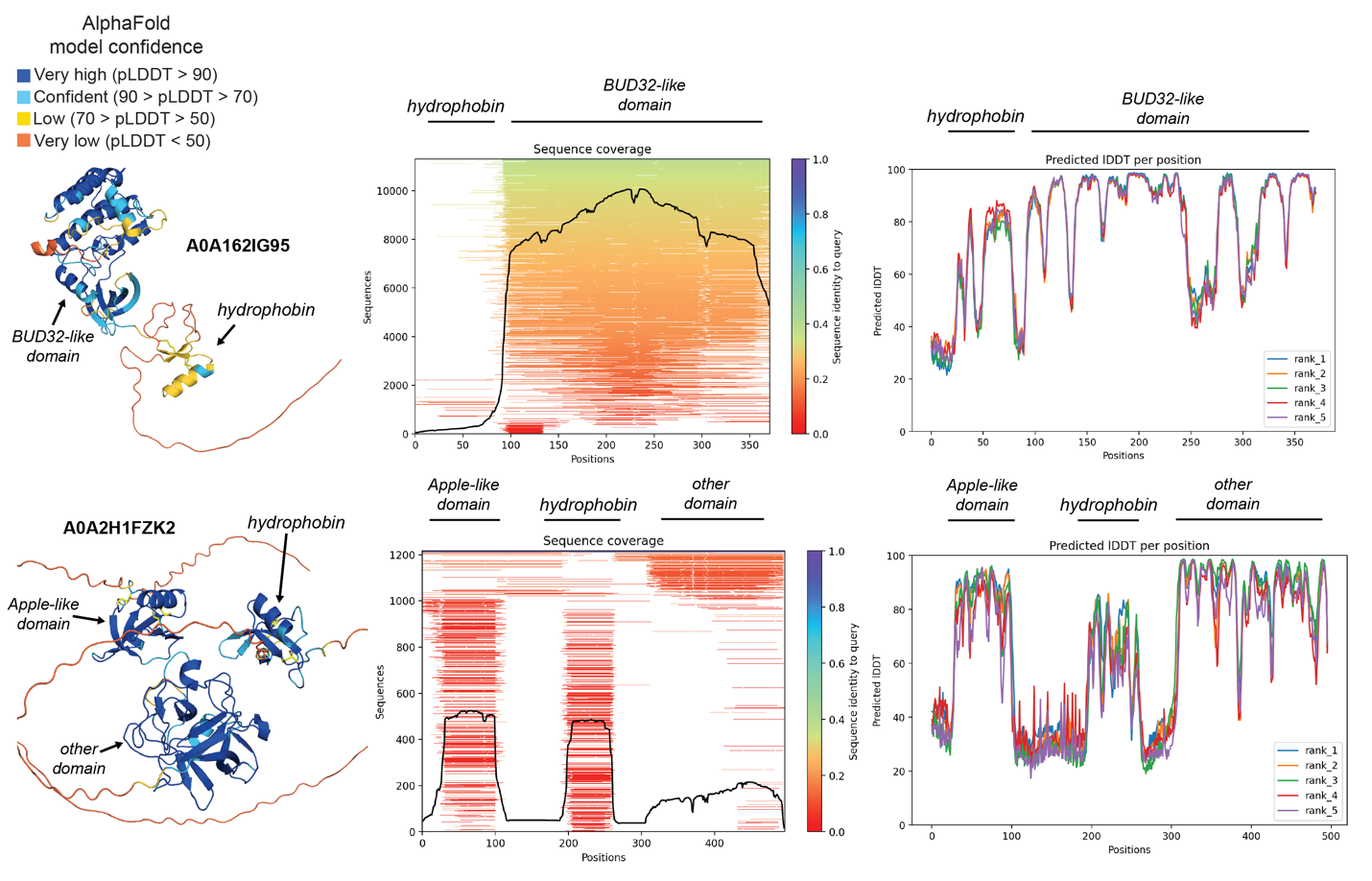


**Supplementary Figure 21.** **MSA coverage and pLDDT scores for two hydrophobin domains within a single polypeptide chain containing non-hydrophobin domains.** AlphaFold models, heat-map representation of the MSA coverage, and predicted lDDT per position are shown for a hydrophobin domain with low pLDDT (top, UniProt #A0A162IG95) and high pLDDT (bottom, UniProt #A0A2H1FZK2). The middle graphs are the heat-map representation of the MSA where homologous sequences mapped to the input sequence. The color scale indicates the sequence identity score (low in red, high in blue). The hit sequences are ordered from highest identity to lowest identity. The white regions are not well covered. The black line denotes the relative coverage of the sequence with respect to the total number of aligned sequences as a function of input sequence position.

**
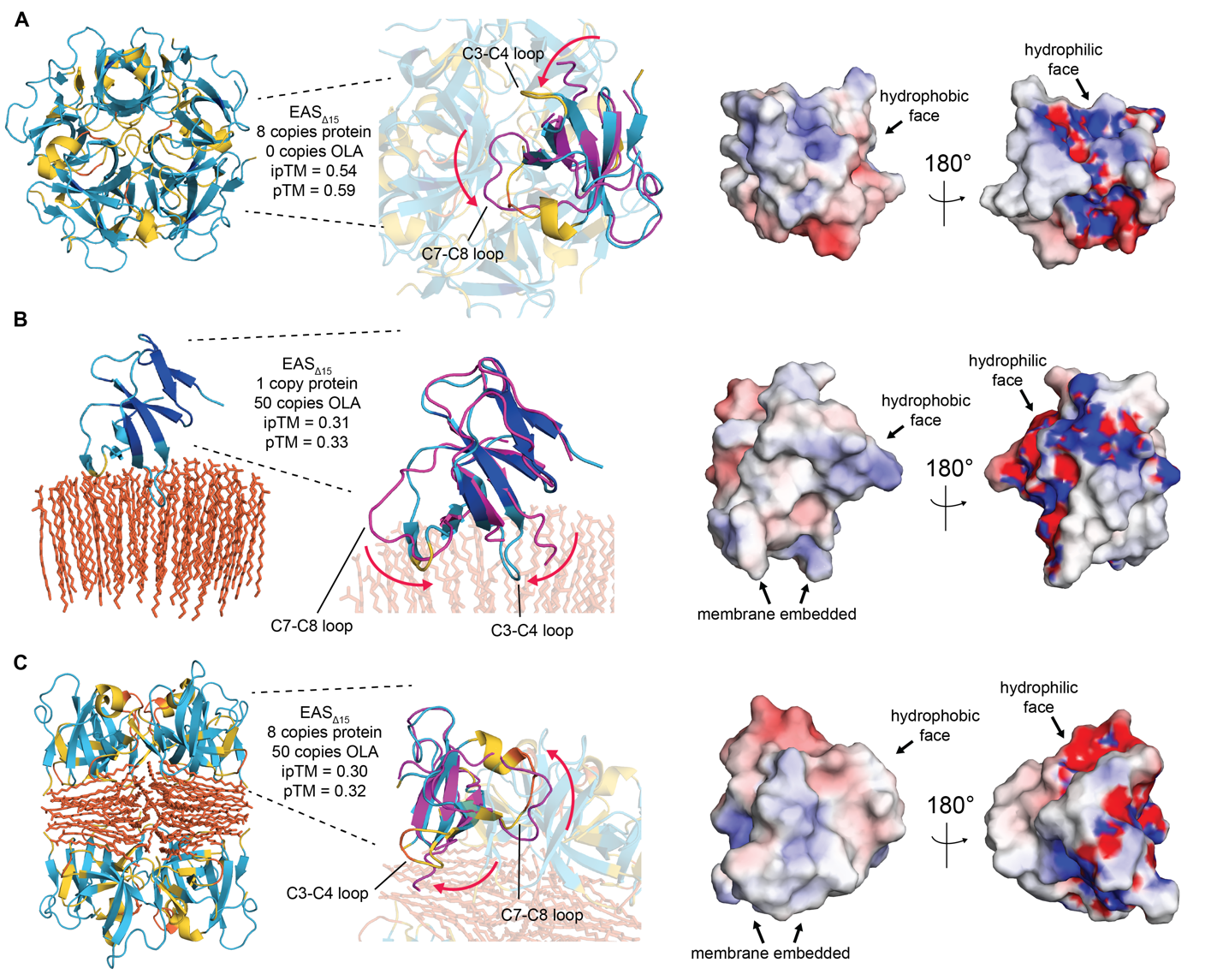
Supplementary Figure 22.** **Representative attempts at AlphaFold3 modeling of class I hydrophobin membrane binding and self-assembly.** (A) Eight copies of EAS Δ15 in the absence of oleic acid. (B) 1 copy of EAS Δ15 and 50 copies of oleic acid. (C) 8 copies of EAS Δ15 and 50 copies of oleic acid. Runs were performed on the AlphaFold3 on a high performance cluster with 104 different model seeds where the top ranked structure (based on pTM) is shown. The top ranked structure is shown but there were not major differences in the output structures. The location of the C3-C4 and C7-C8 functional loops are noted with red arrows noting conformational changes between AlphaFold models and the experimental structure. In panels A to C, examples of overlays with EAS Δ15 from PDB ID 2K6A (in magenta) are shown. AlphaFold models are accompanied by an electrostatic surface visualization calculated with the Adaptive Poisson-Boltzmann Solver (APBS plugin of PyMOL) ^110^. The contour scale for the APBS visualization is -5 kT/e (red, negative) to +5 kT/e (blue, positive). The hydrophobic (white) and hydrophilic (dark blue/dark red) faces of the amphipathic hydrophobin surface are noted. The location of the surfaces undergoing membrane embedding are noted.


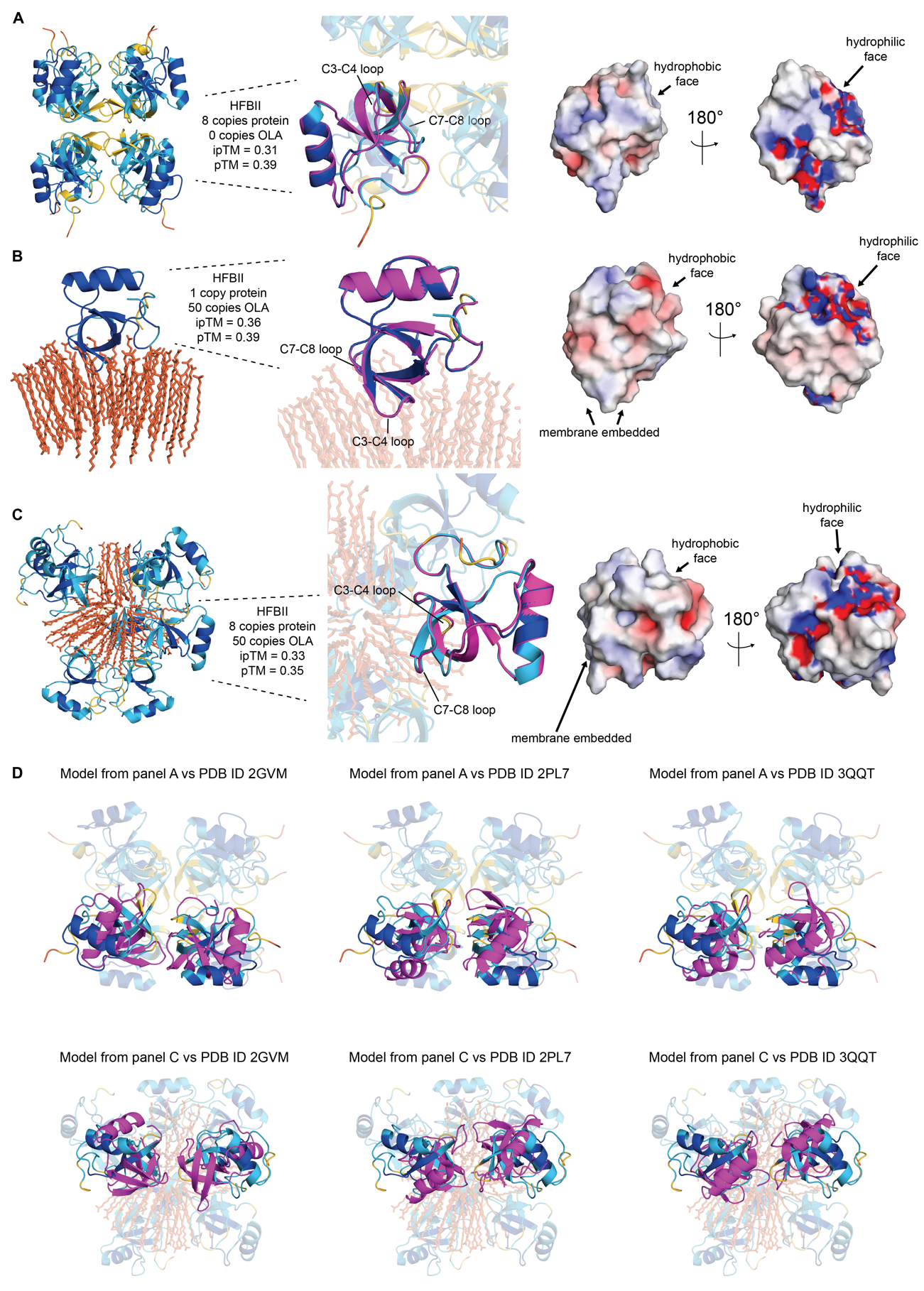


**Supplementary Figure 23.** **Representative attempts at AlphaFold3 modeling of class II hydrophobin detergent binding and self-assembly.** (A) Eight copies of HFBII in the absence of oleic acid. (B) 1 copy of HFBII and 50 copies of oleic acid. (C) 8 copies of HFBII and 50 copies of oleic acid. Runs were performed on the AlphaFold3 on a high performance cluster with 104 different model seeds where the top ranked structure (based on pTM) is shown. The top ranked structure (ipTM score) is shown but there were not major differences in the output structures. The location of the C3-C4 and C7-C8 functional loops are noted with no significant conformational changes between AlphaFold models and the experimental structure occurring. In panels A to C, examples of overlays with HFBII from PDB ID 2PL7 (in magenta) are shown. AlphaFold models are accompanied by an electrostatic surface visualization calculated with the Adaptive Poisson-Boltzmann Solver (APBS plugin of PyMOL) ^110^. The contour scale for the APBS visualization is -5 kT/e (red, negative) to +5 kT/e (blue, positive). The hydrophobic (white) and hydrophilic (dark blue/dark red) faces of the amphipathic hydrophobin surface are noted. The location of the surfaces undergoing membrane embedding are noted. (D) Overlay of AlphaFold3 models of self-assembled hydrophobins with experimental structure of HFBII in the presence of detergents (magenta cartoon, PDB IDs are mentioned for each).


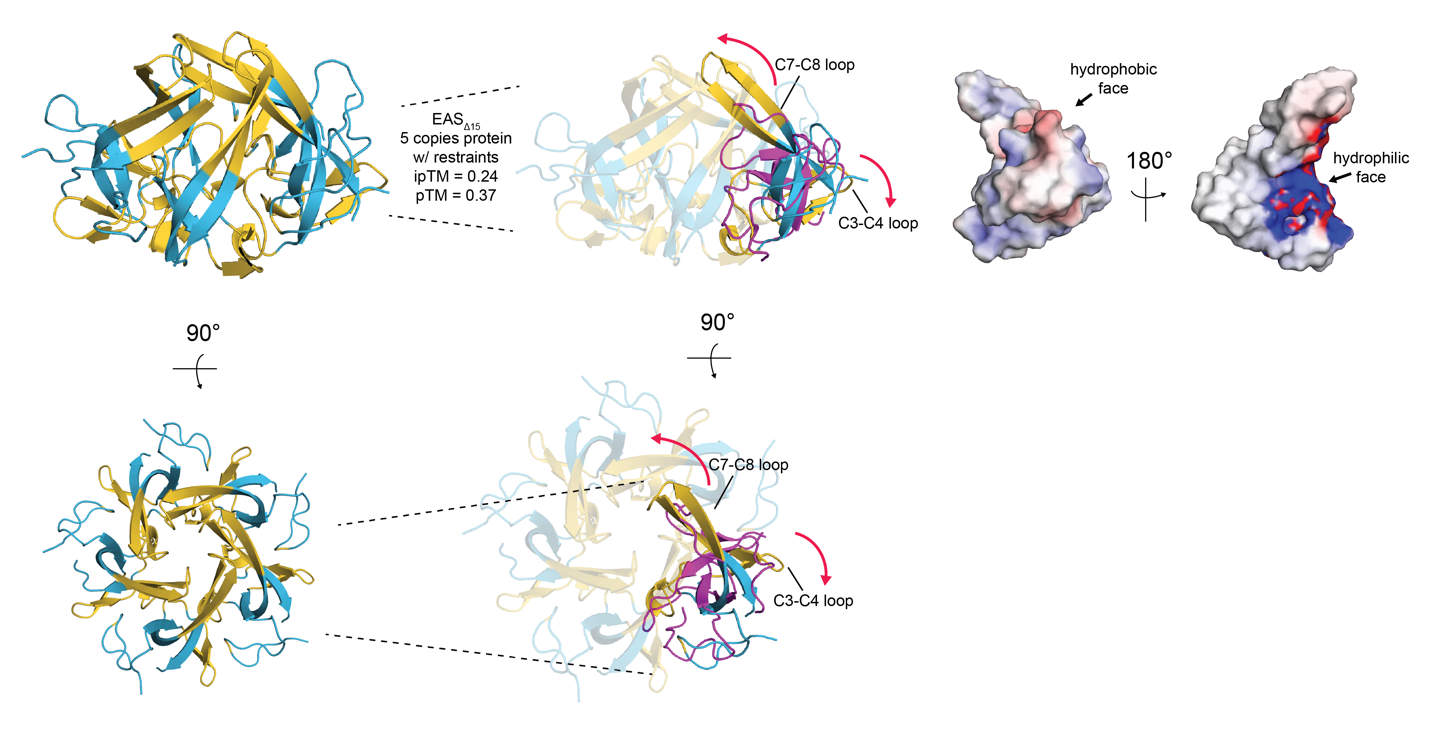


**Supplementary Figure 24.** **Representative attempts at Chai-1 modeling of class I hydrophobin self-assembly using restraints.** Five copies of EAS Δ15 in the absence of oleic acid with restraints between TNT (residues 52 to 54 based on numbering in PDB 2K6A) and FLI (residues 58 to 60 based on numbering in PDB 2K6A) provided as defined in the Methods section. Runs were performed on the Chai-1 webserver where the top ranked structure (based on pTM) is shown. The location of the C3-C4 and C7-C8 functional loops are noted with red arrows noting conformational changes between Chai-1 models and the experimental structure. Example overlay shown with EAS Δ15 from PDB ID 2K6A (in magenta). Chai-1 model is accompanied by an electrostatic surface visualization calculated with the Adaptive Poisson-Boltzmann Solver (APBS plugin of PyMOL) ^110^. The contour scale for the APBS visualization is -5 kT/e (red, negative) to +5 kT/e (blue, positive). The hydrophobic (white) and hydrophilic (dark blue/dark red) faces of the amphipathic hydrophobin surface are noted.

**Supplementary Alignment 1.** **Sequence alignment of class I hydrophobins with extended N-terminal tails.** Performed with Clustal Omega 1.2.4 and processed with ESPript 3.

**
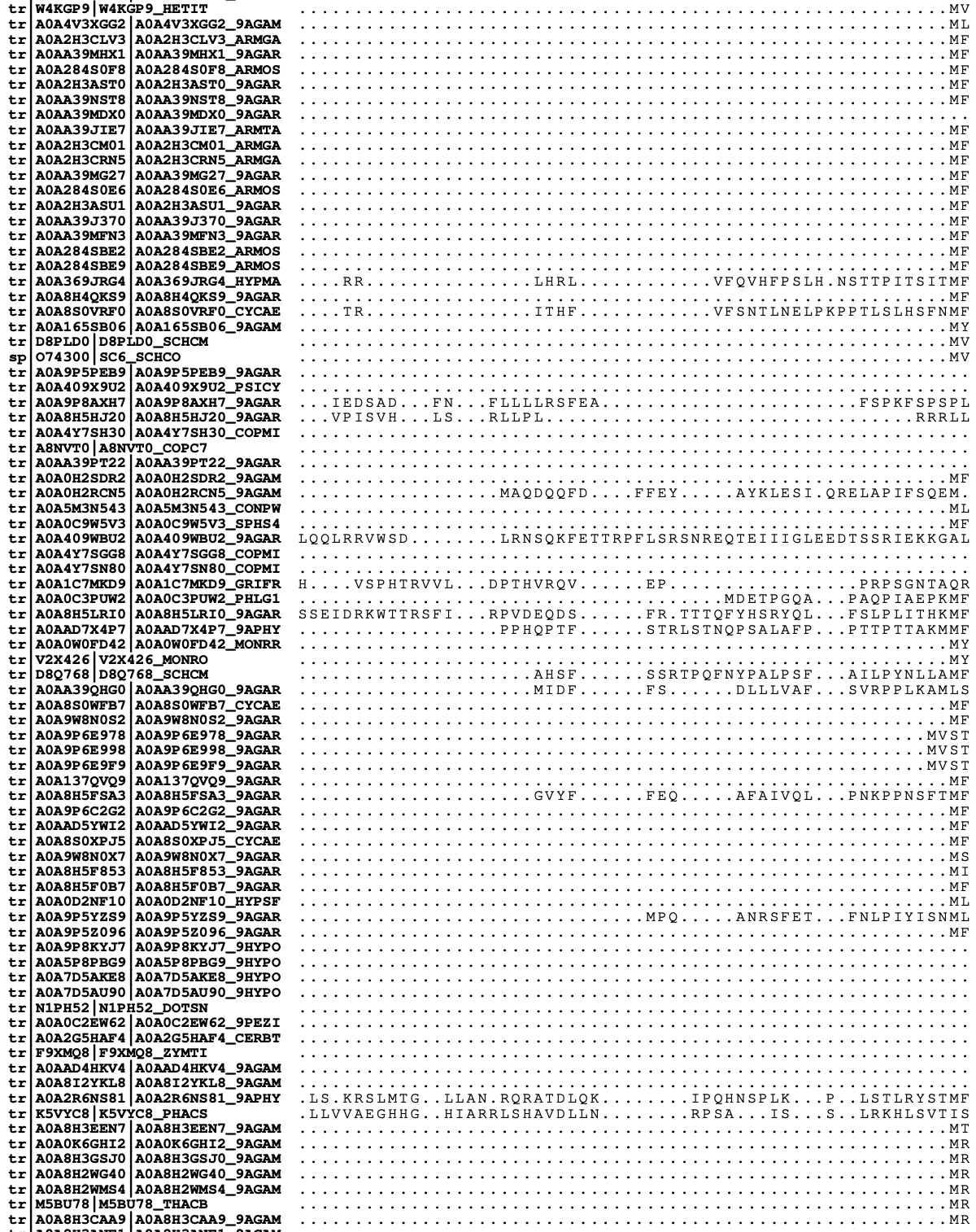
**

**
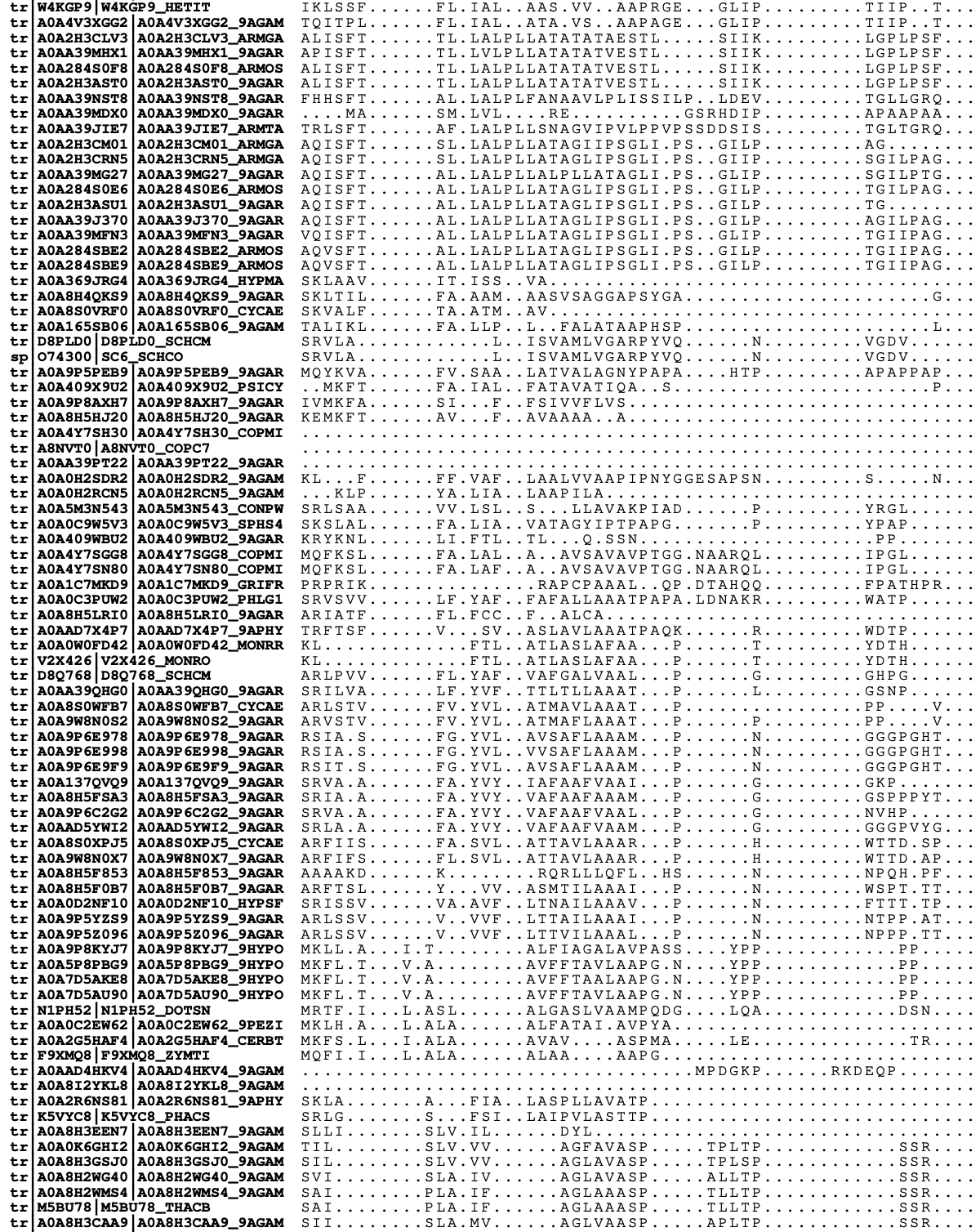
**

**
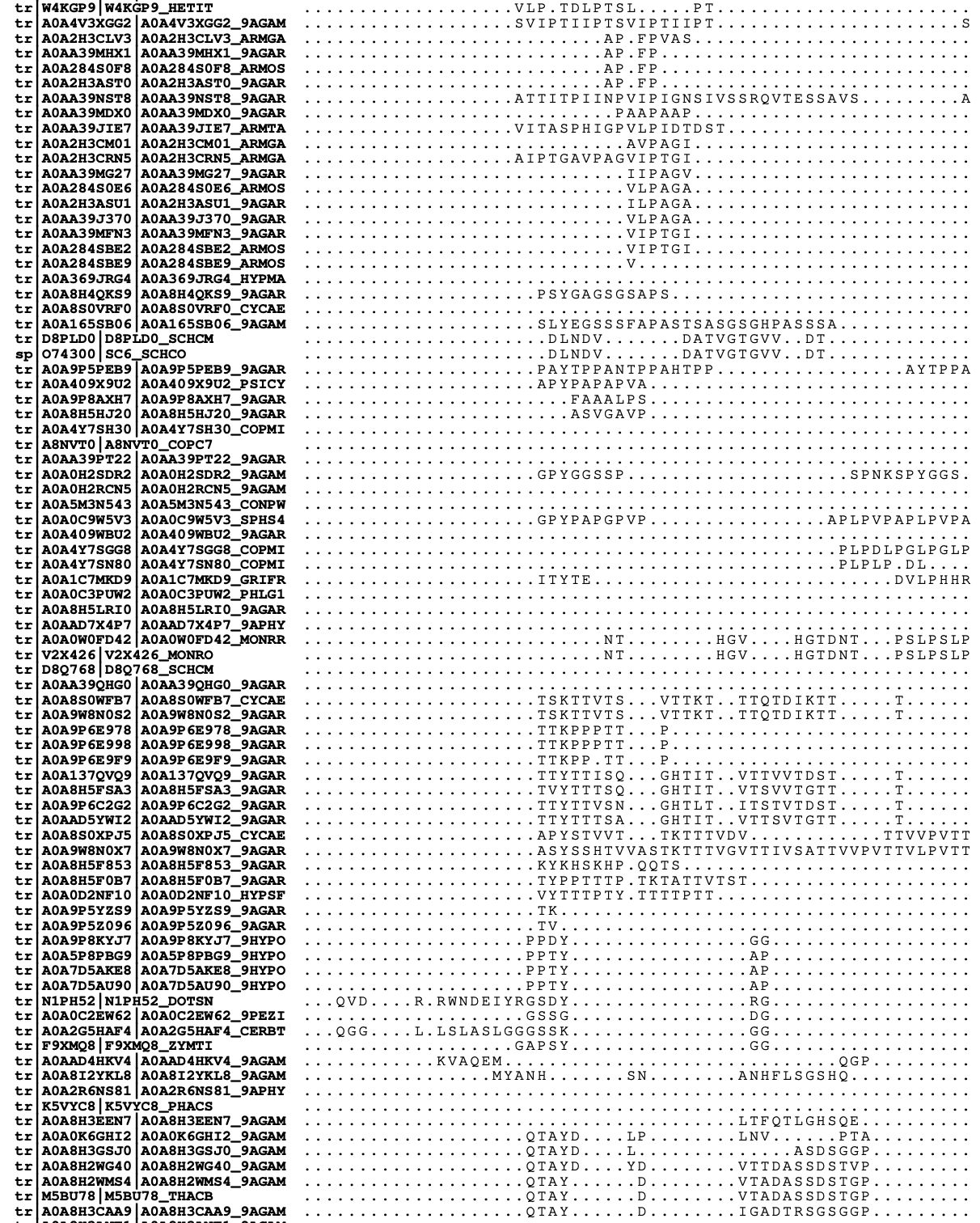
**

**
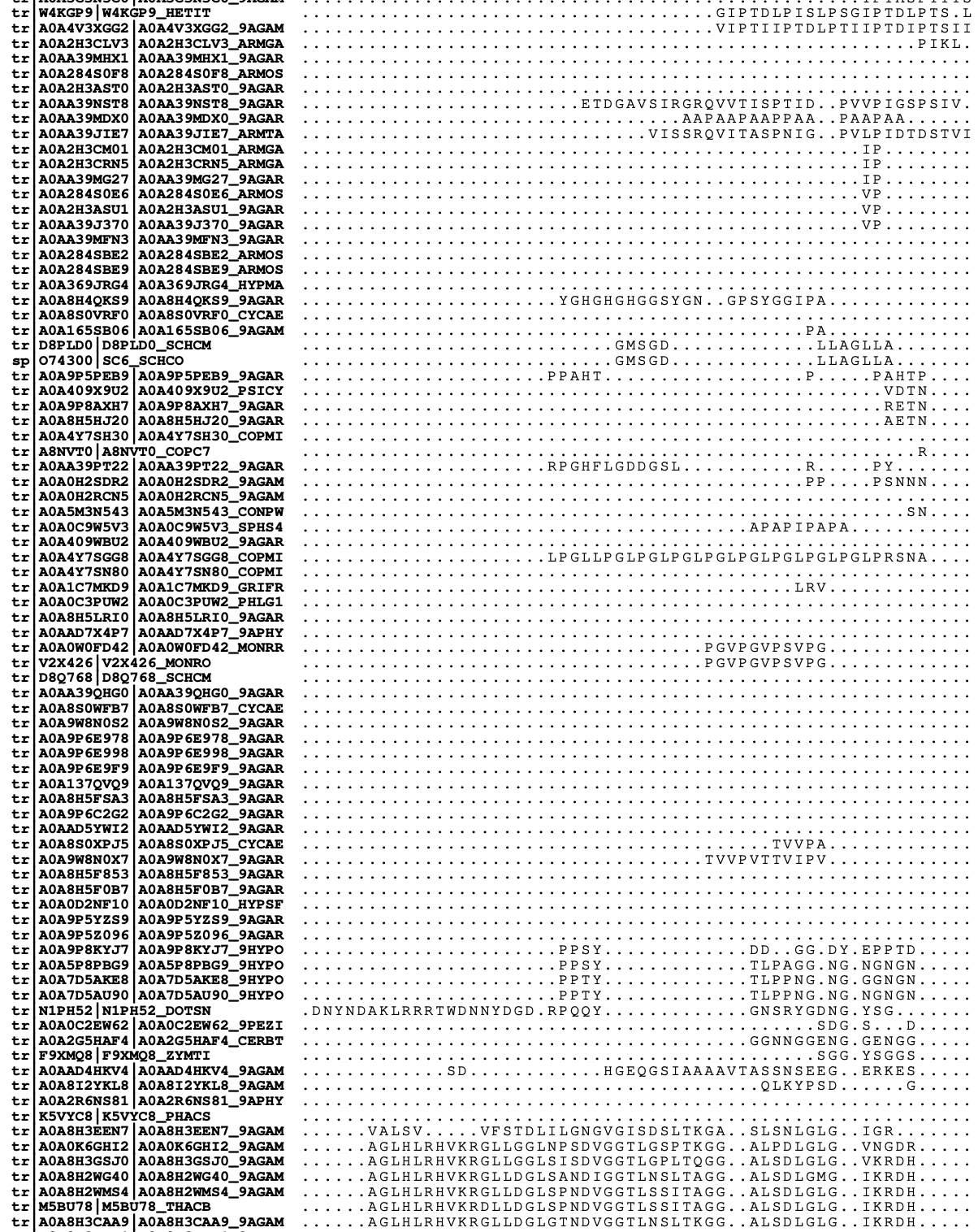
**

**
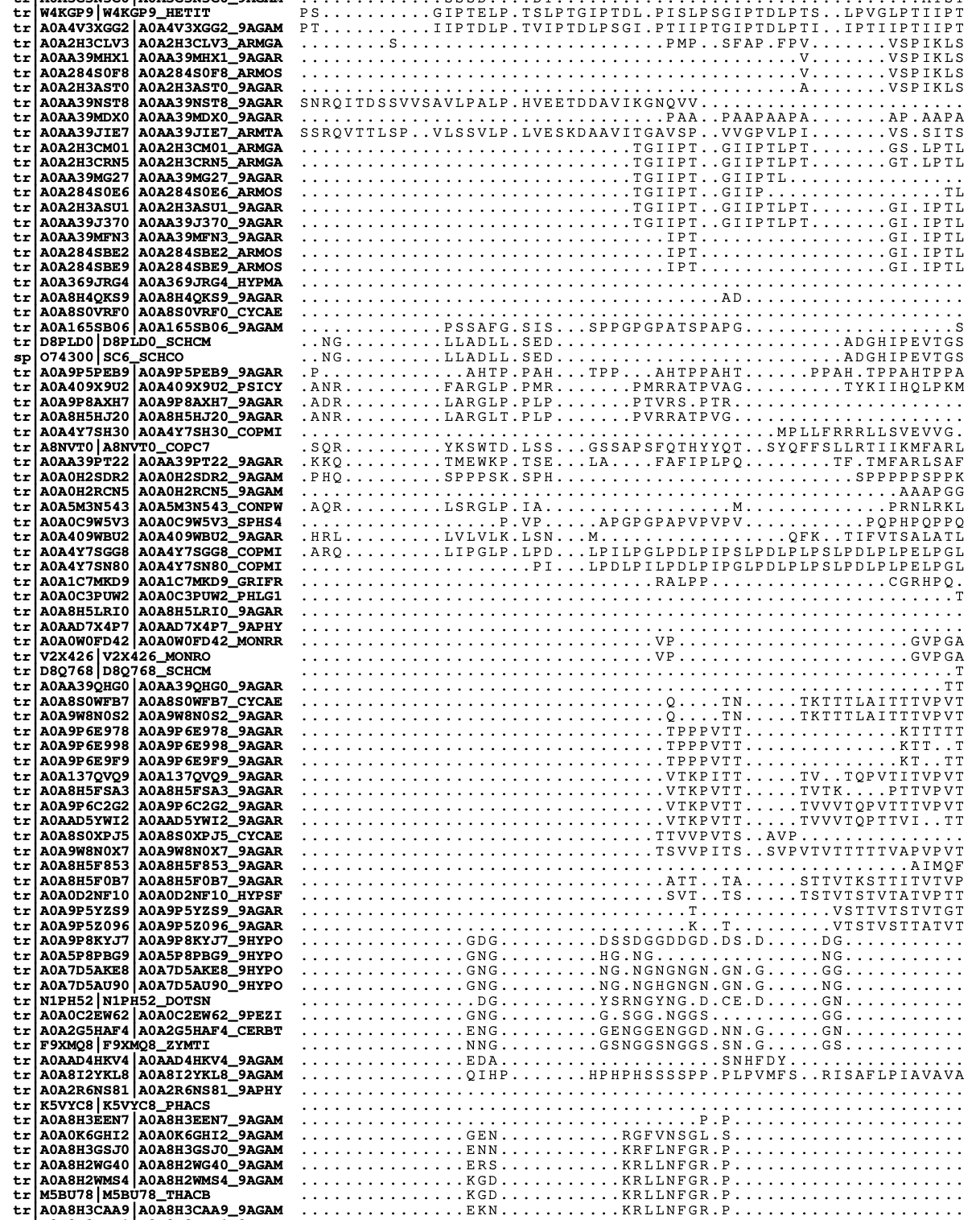
**

**
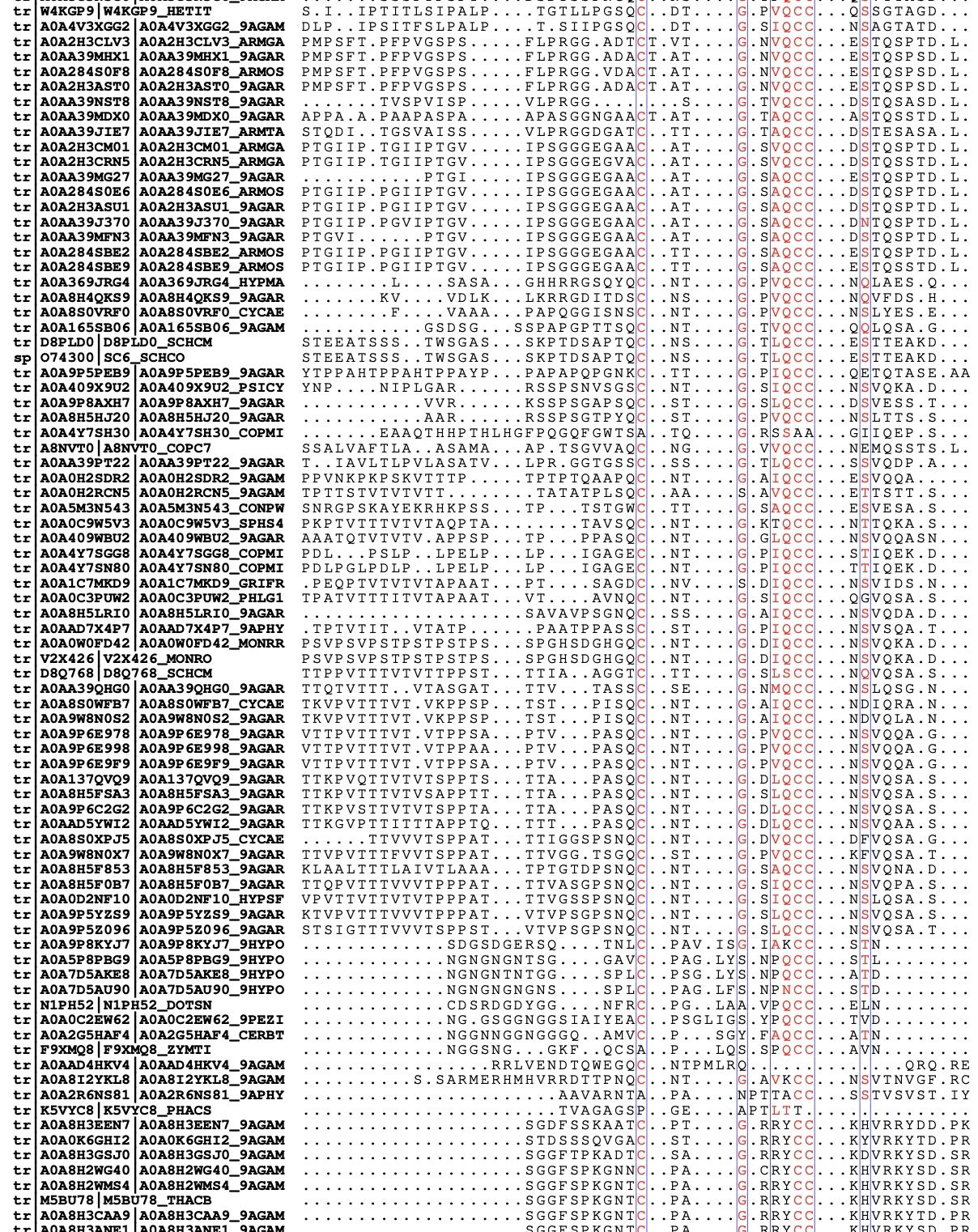
**

**
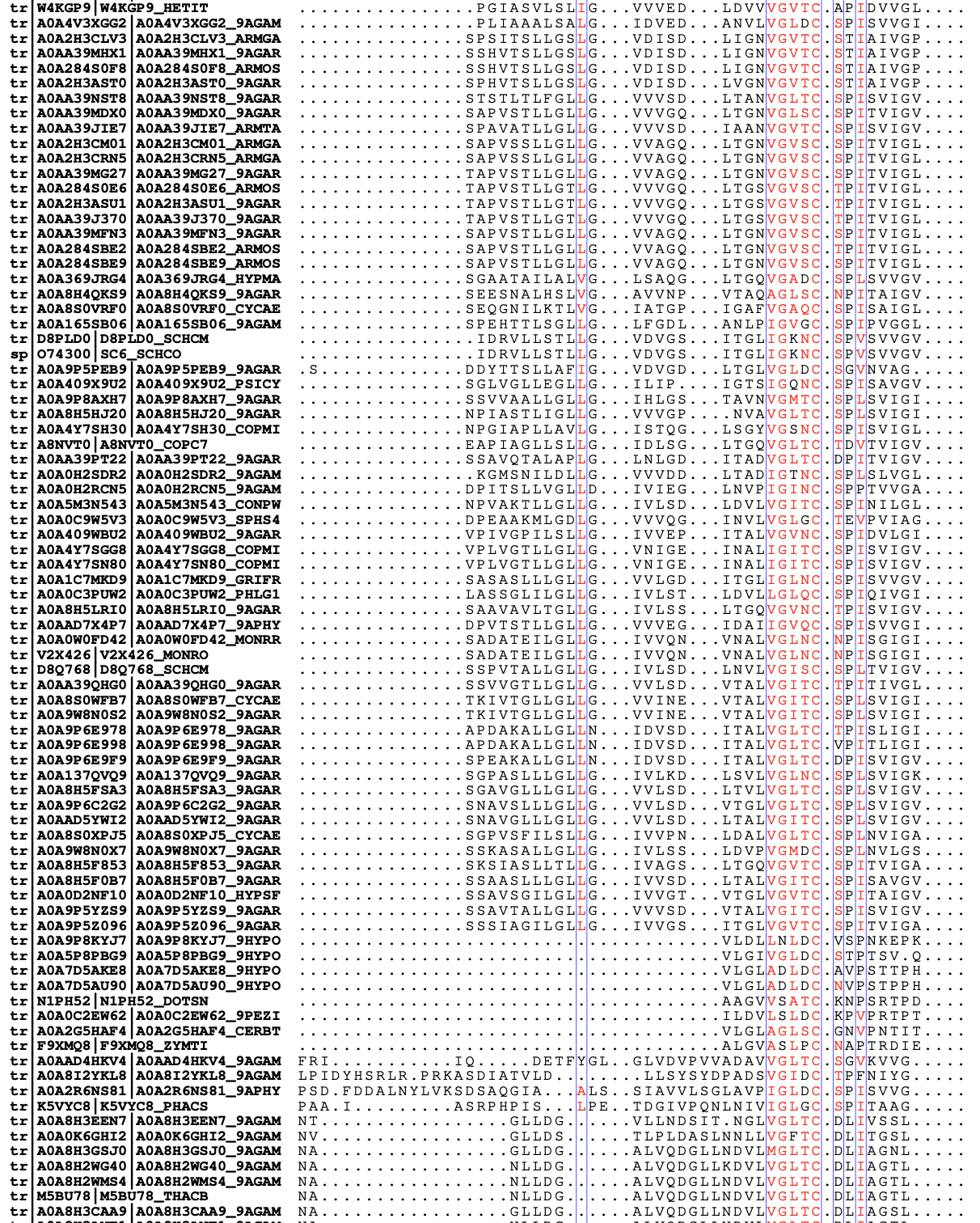
**

**
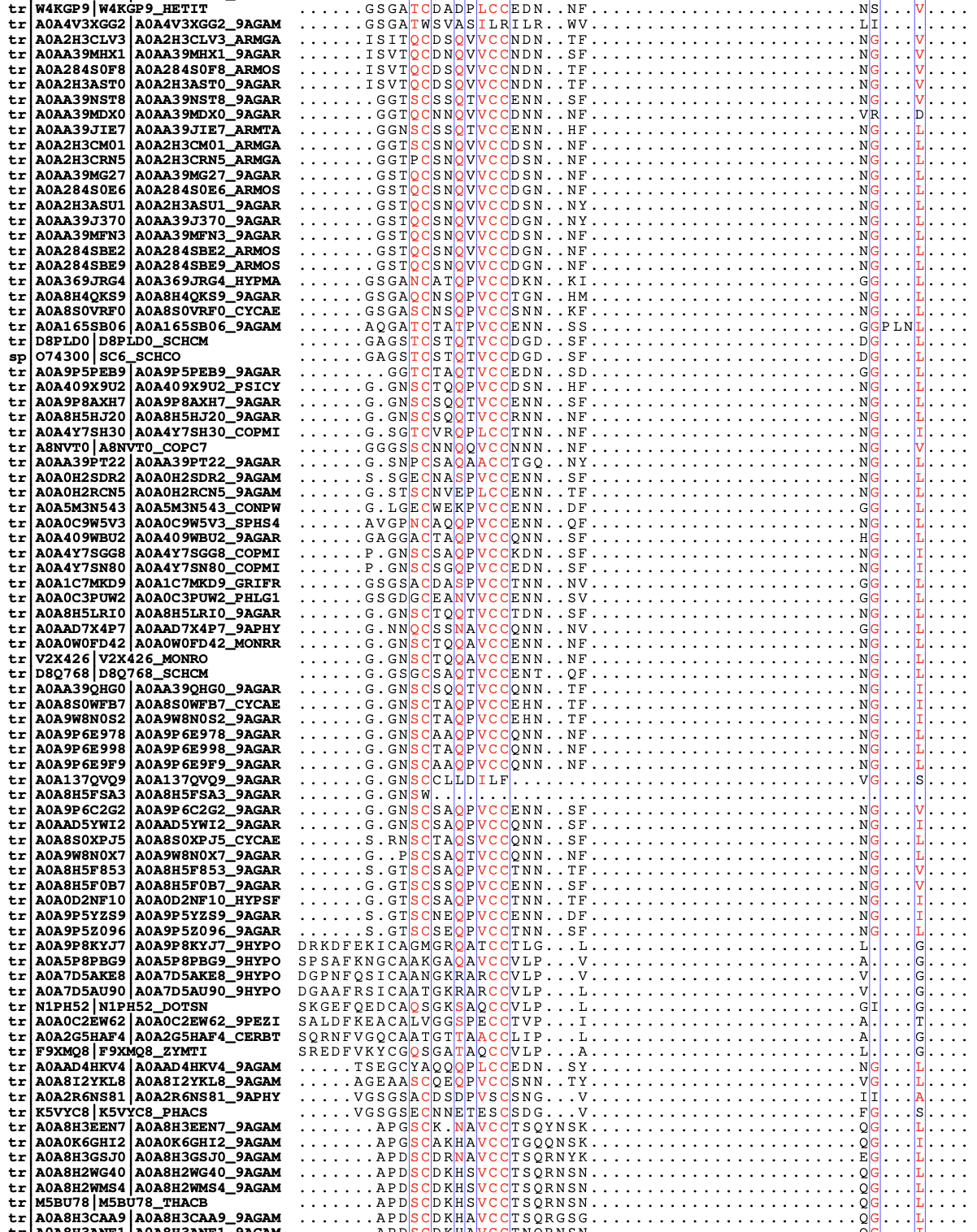
**

**
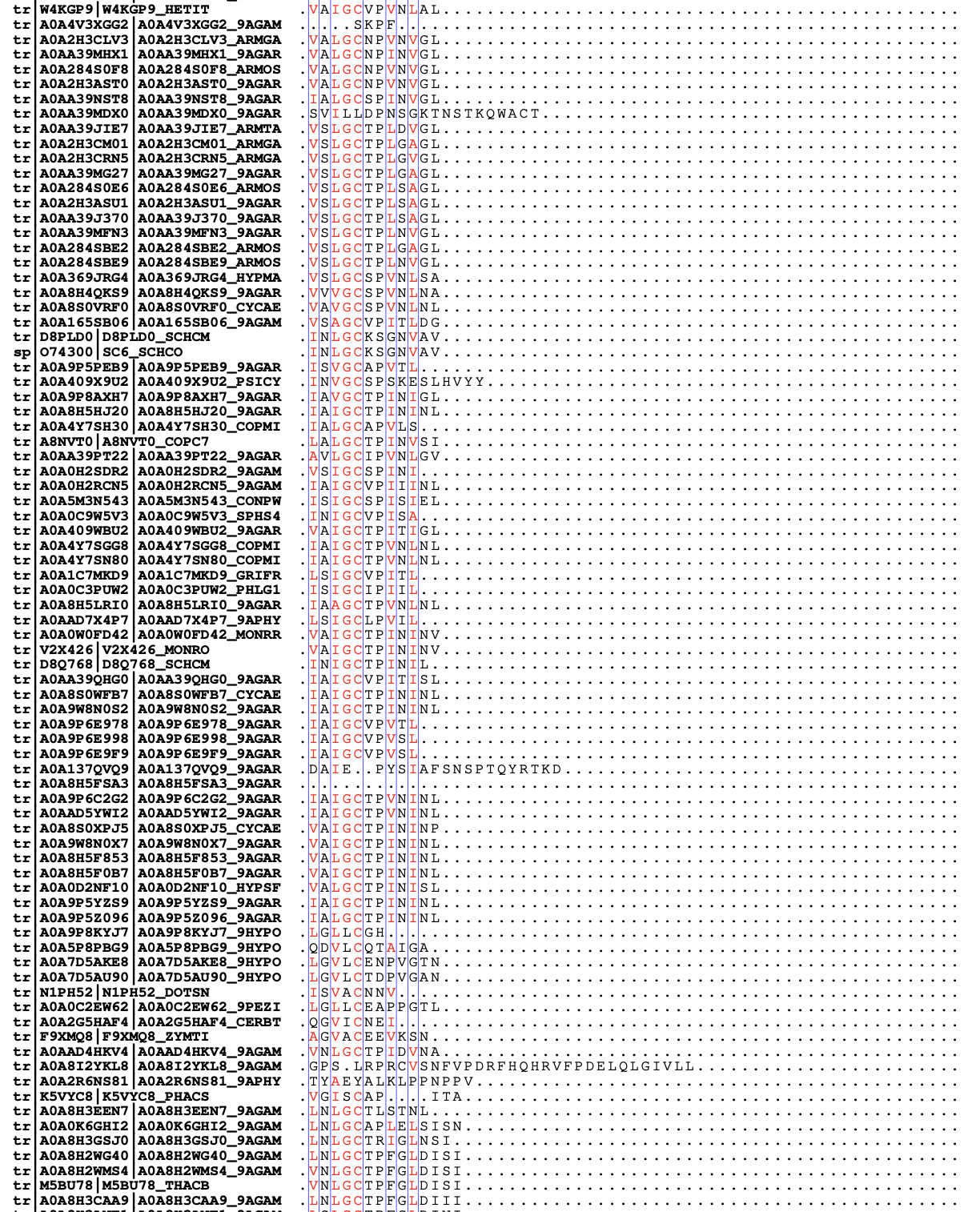
**

**Supplementary Alignment 2.** **Sequence alignment of class II hydrophobins with extended N-terminal tails.** Performed with Clustal Omega 1.2.4 and processed with ESPript 3.


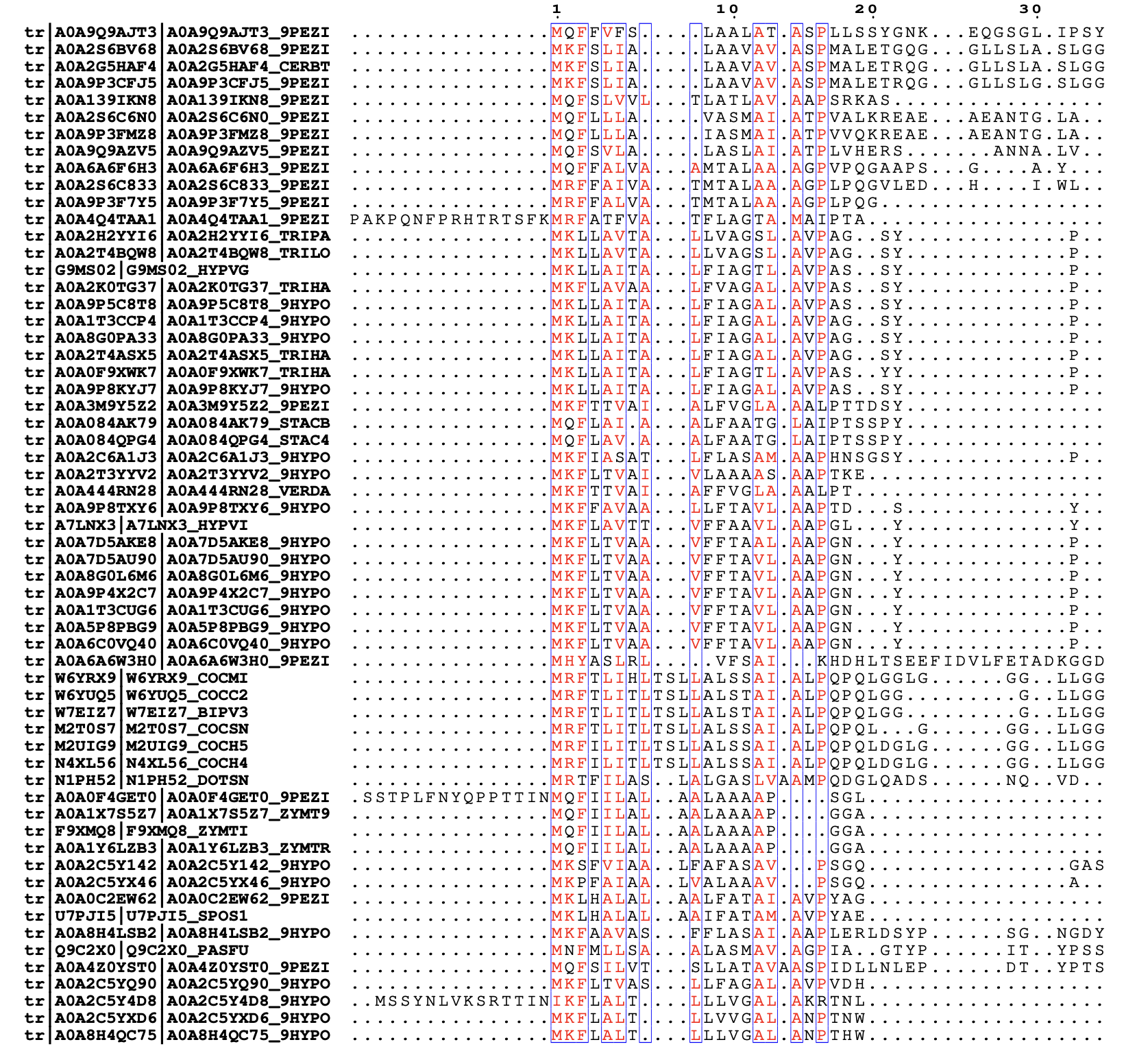


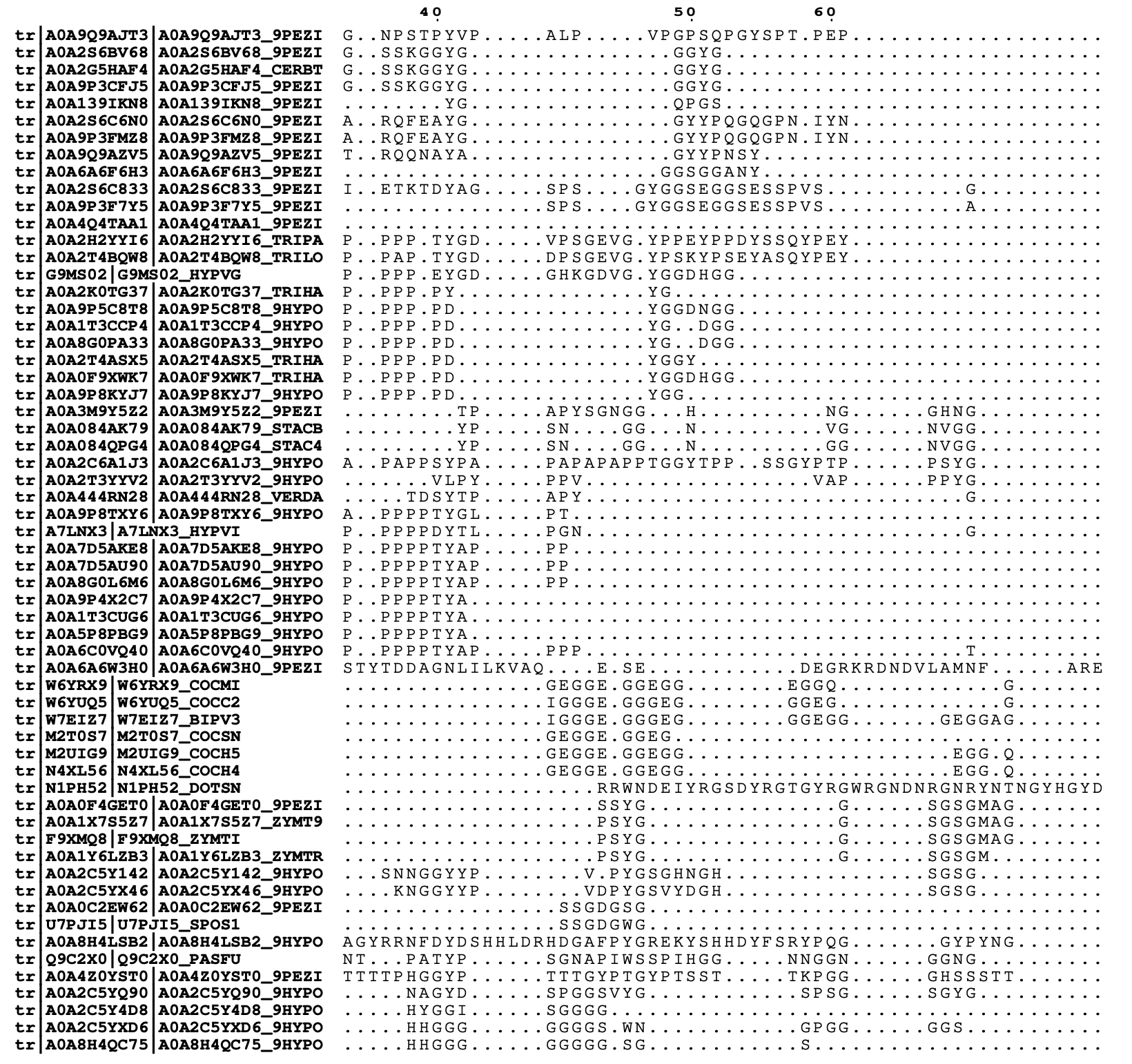


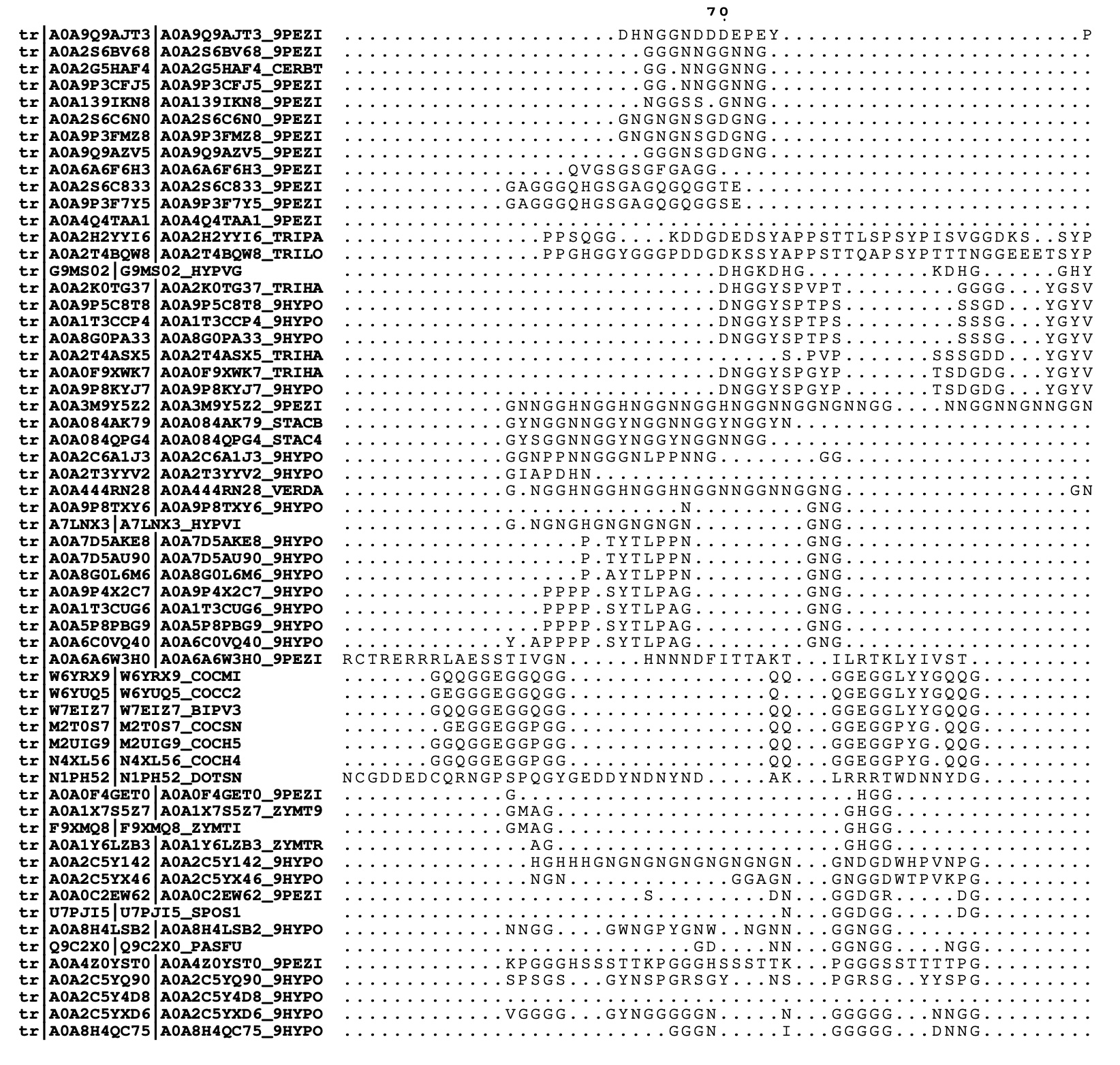


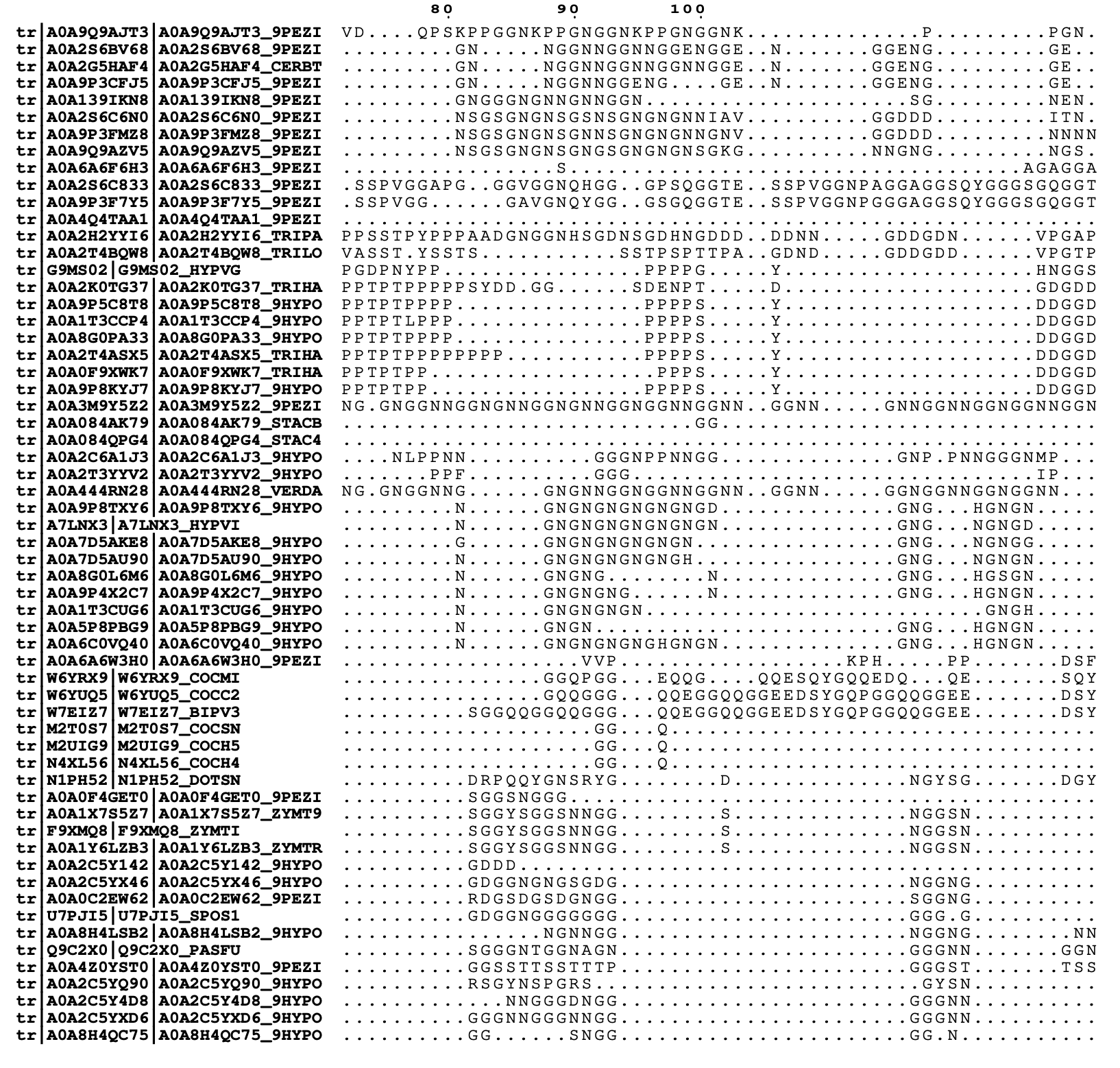


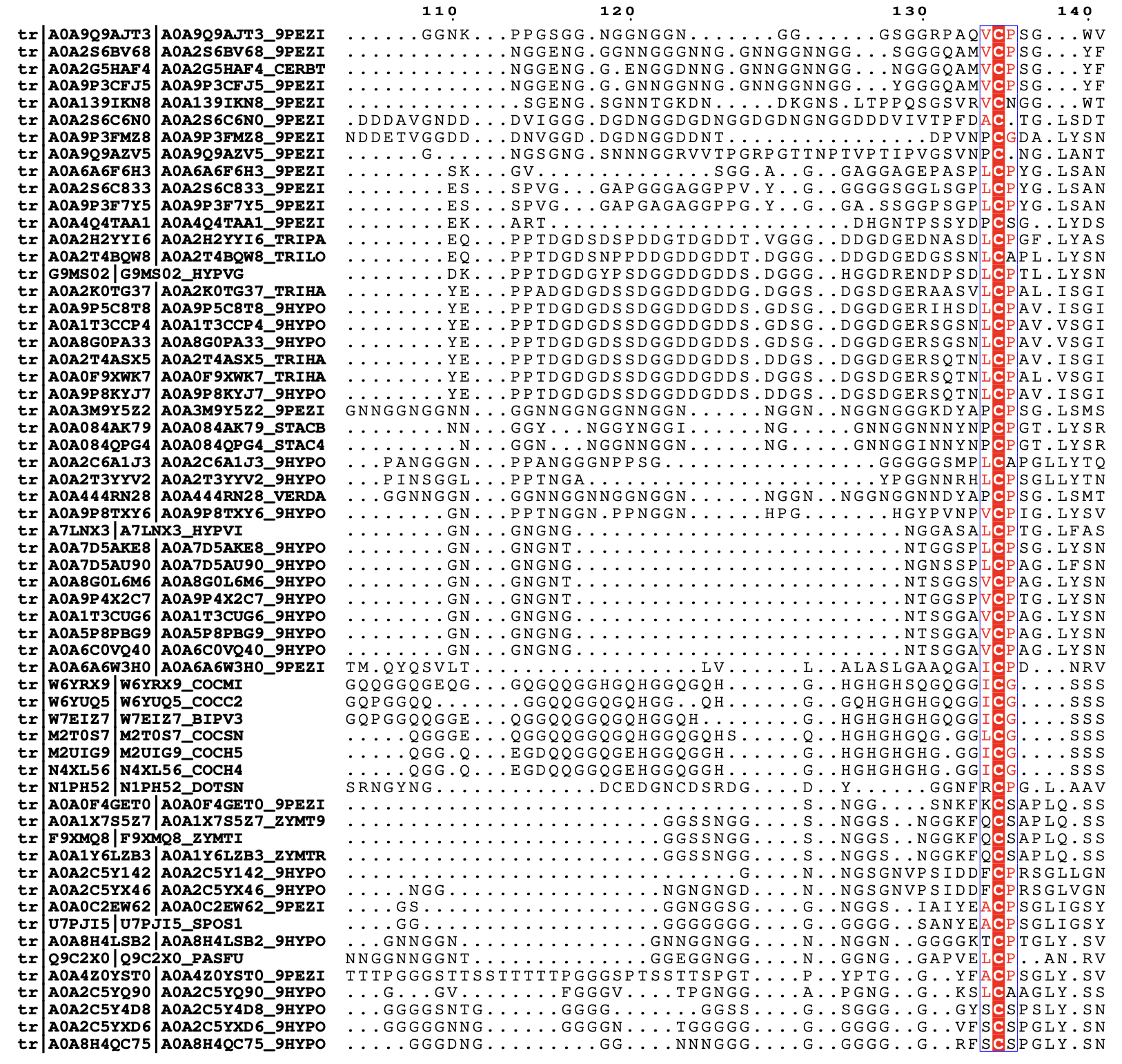

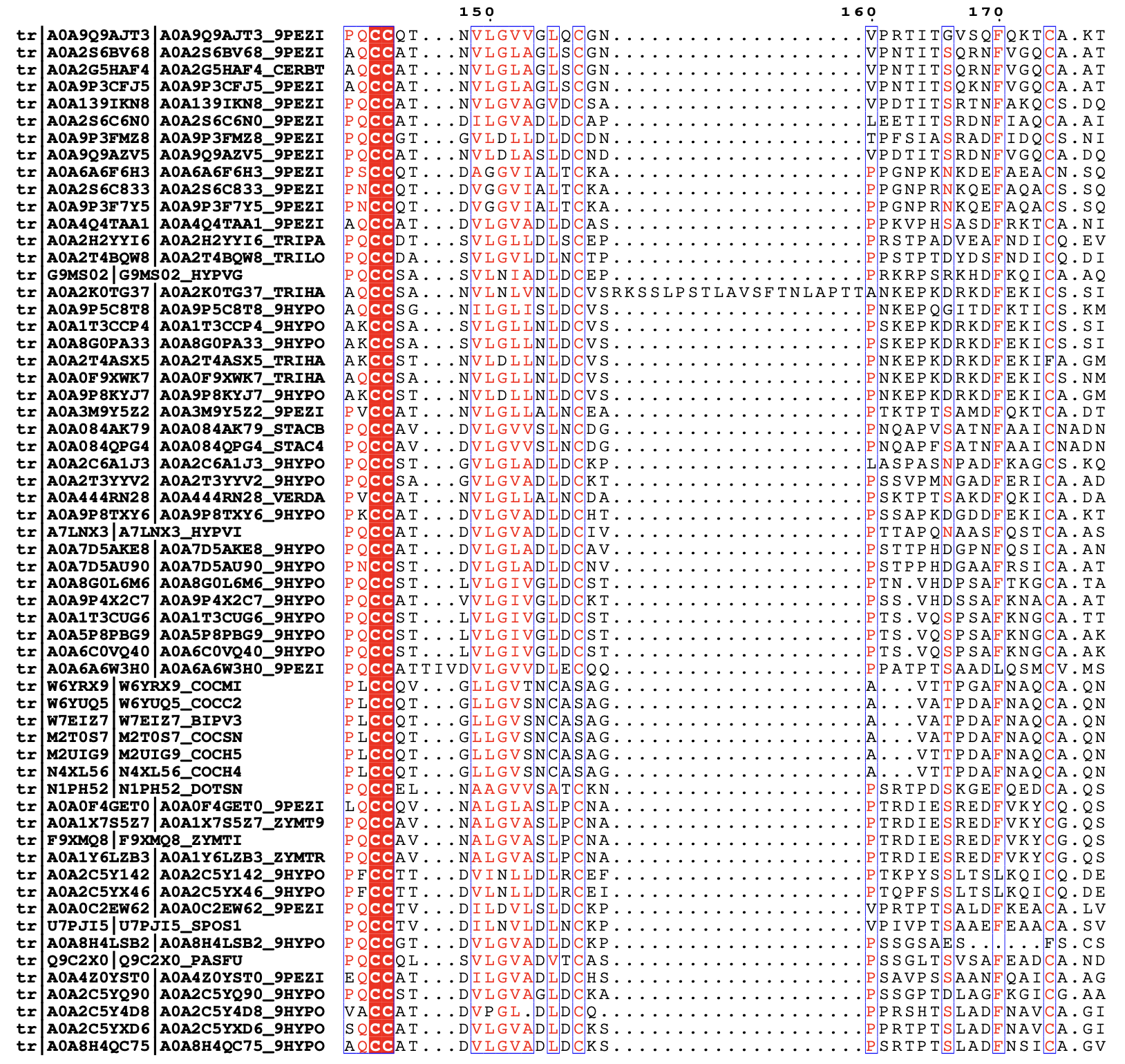

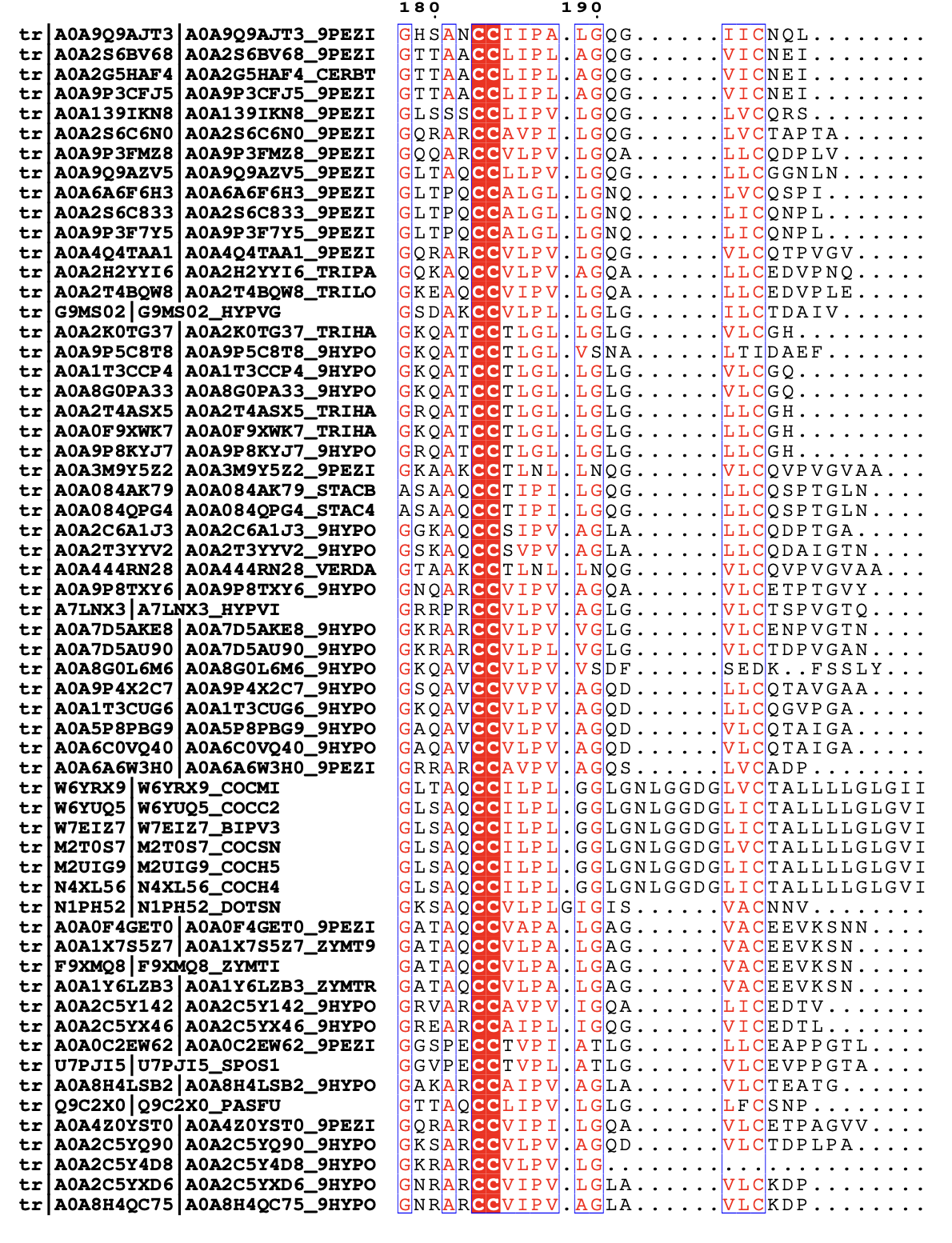

Supplement: Supplementary file 1 — Data S1. Supporting Information. [file PRO-34-e70279-s001.docx]
